# Design and synthesis new indole-based aromatase/iNOS inhibitors with apoptotic antiproliferative activity

**DOI:** 10.3389/fchem.2024.1432920

**Published:** 2024-09-06

**Authors:** Lamya H. Al-Wahaibi, Hesham A. Abou-Zied, Mostafa H. Abdelrahman, Martha M. Morcoss, Laurent Trembleau, Bahaa G. M. Youssif, Stefan Bräse

**Affiliations:** ^1^ Department of Chemistry, College of Sciences, Princess Nourah Bint Abdulrahman University, Riyadh, Saudi Arabia; ^2^ Medicinal Chemistry Department, Faculty of Pharmacy, Deraya University, Minia, Egypt; ^3^ Pharmaceutical Organic Chemistry Department, Faculty of Pharmacy, Al-Azhar University, Assiut, Egypt; ^4^ Department of Pharmaceutical Chemistry, Faculty of Pharmacy, Nahda University, Beni-Suef, Egypt; ^5^ School of Natural and Computing Sciences, University of Aberdeen, Aberdeen, United Kingdom; ^6^ Pharmaceutical Organic Chemistry Department, Faculty of Pharmacy, Assiut University, Assiut, Egypt; ^7^ Institute of Biological and Chemical Systems, IBCS-FMS, Karlsruhe Institute of Technology, Karlsruhe, Germany

**Keywords:** indole, pyrazine, aromatase, nitric oxide, synthase, inhibitors

## Abstract

The present study details the design, synthesis, and bio-evaluation of indoles **3–16** as dual inhibitors of aromatase and inducible nitric oxide synthase (iNOS)with antiproliferative activity. The study evaluates the antiproliferative efficacy of **3–16** against various cancer cell lines, highlighting hybrids **12** and **16** for their exceptional activity with GI_50_ values of 25 nM and 28 nM, respectively. The inhibitory effects of the most active hybrids **5, 7, 12**, and **16**, on both aromatase and iNOS were evaluated. Compounds **12** and **16** were investigated for their apoptotic potential activity, and the results showed that the studied compounds enhance apoptosis by activating caspase-3, 8, and Bax and down-regulating the anti-apoptotic Bcl-2. Molecular docking studies are intricately discussed to confirm most active hybrids’ **12-** and **16**-binding interactions with the aromatase active site. Additionally, our novel study discussed the ADME characteristics of derivatives **8–16**, highlighting their potential as therapeutic agents with reduced toxicity.

## 1 Introduction

Aromatase, a member of the cytochrome P450 family (CYP19), is required for estrogen biosynthesis. CYP19 catalyzes a series of three hydroxylation reactions to transform C19 androgens (androstenedione and testosterone) into aromatic C18 estrogens (estradiol and estrone) (Graham Lorence et al., 1995). It is well established that elevated estrogen levels, both before and after menopause, promote hormone-dependent breast cancer (HDBC) and the metastasis of cancer cells to other parts of the body in women (Zhao et al., 2016). Aromatase inhibition reduces estrogen levels, which proves essential for controlling hormone-sensitive breast cancer. Aromatase is an important enzyme in the production of estrogen, especially after menopause. Aromatase inhibitors have been shown to be effective chemopreventive medicines in HDBC (Riemsma et al., 2010; Maghraby et al., 2023).

**FIGURE 1:**
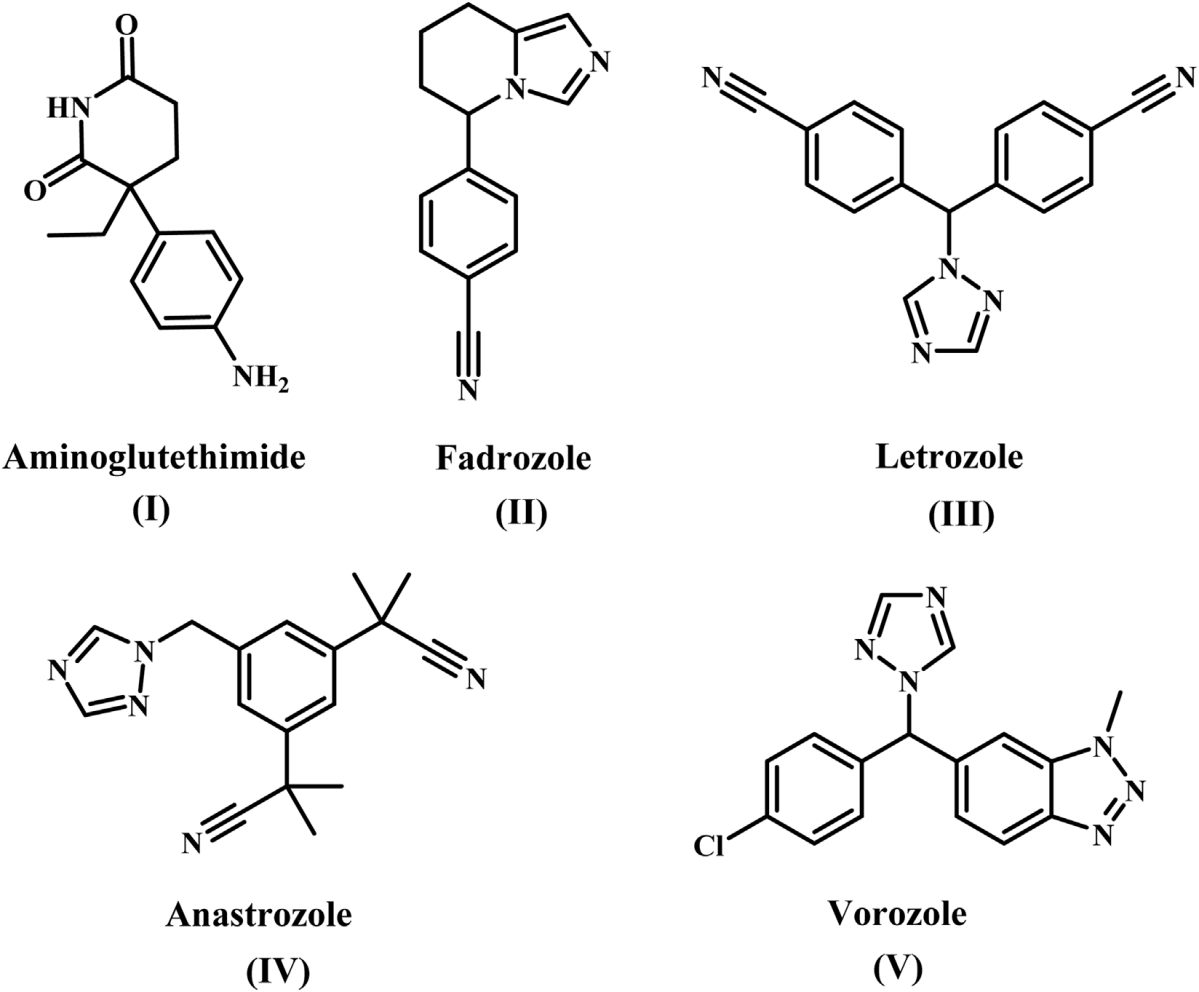
Structure of some nonsteroidal aromatase inhibitors **(I-V)**.

Aromatase inhibitors (AIs) are classified structurally into steroidal and nonsteroidal derivatives, which appear to differ in their ability to interact with the enzyme (Mokbel, 2002; Dixon et al., 2003). The most studied nonsteroidal inhibitor was aminoglutethimide [**AG**, 3-(4-aminophenyl)-3-ethylpiperidine-2,6-dione, **compound I**], Figure 1 (Brodie et al., 1993), 4-substituted anilines (Hartmann et al., 1992; Chen et al., 2021), imidazole antifungals as well as analogs (Karjalainen et al., 2000; Saxena et al., 2015). AG, The first commercially available nonsteroidal inhibitor, was released in 1981 (Santen and Henderson, 1982). However, due to its lack of specificity against other cytochrome P450 enzymes and inherent toxicity, the use of AG has been limited (Foti, 2023). Many research teams’ investigations have discovered several heterocyclic azoles (Recanatini et al., 2002; Rani et al., 2021; Kharb et al., 2020; Avvaru et al., 2018), such as Fadrozole **(II)**, Anastrozole **(III)**, Letrozole **(IV)**, and Vorozole **(V)** (Figure 1). These nonsteroidal aromatase inhibitors exhibited improved selectivity and a significant increase in potency.

**FIGURE 2:**
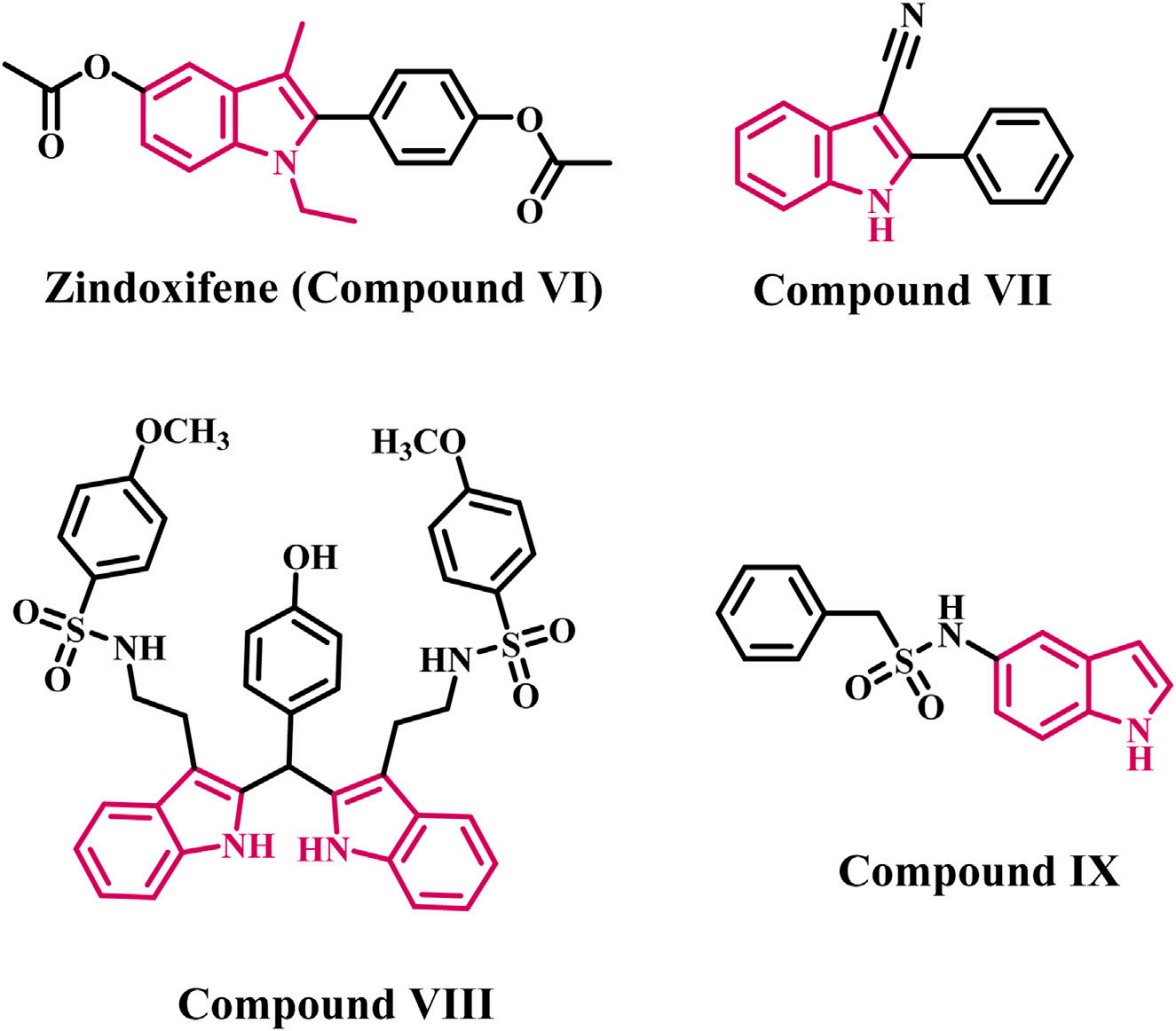
Structures of indole-based aromatase inhibitors **(VI-IX)**.

Indole is a valuable scaffold in medicinal chemistry because it is the building block for many important natural substances, such as serotonin, tryptophan, and tryptamine (Kaushik et al., 2013). Even though indole has a variety of therapeutic applications, the indole nucleus received increased attention in the AI class after the approval of Zindoxifene (**compound VI**, Figure 2), a strong anti-estrogen (Fürstner et al., 1994). Given the importance of indole heterocycles in medicinal chemistry, Prior *et al.* synthesized and tested a series of novel 2-aryl indoles for aromatase inhibition (Prior et al., 2017). With an IC_50_ value of 1.61 μM, **compound VII** (Figure 2) containing a nitrile (CN) group at indole’s C-3 position was the most potent. The CN group lost its potency twice when it was moved to the indole’s C-5 position (IC_50_ = 3.34 μM). According to SAR, analogs with EWG substituted at position C-3 rather than C-5 exhibited excellent aromatase inhibition activity.

Pingaew *et al.* synthesized a series of *bis*- and *tris*-indoles with sulfonamides substituted with EWG (electron withdrawing group) and EDG (electron donating group) to develop effective AI (Pingaew et al., 2018). The *bis*-indole derivative (**compound VIII**, Figure 2) was discovered to be the most potent derivative, with an IC_50_ value of 0.7 µM. The biological results showed that activity decreased when EWG was used instead of the methoxy group. Furthermore, it was discovered that *bis*-indole derivatives were more effective than *tris*-indoles.

Amoroso and colleagues developed indole aryl sulfonamides as effective AIs (Fantacuzzi et al., 2020). The most potent AI was derivative **IX** (Figure 2), which had an indole ring at the C-5 position and an IC_50_ value of 0.16 µM. The potency is lost when aryl sulfonamide is directly substituted with the 3-/6-position of indole. According to biological findings, the attachment position of the indole ring plays an important role in the aromatase inhibition activity of indole aryl sulfonamides.

**FIGURE 3:**
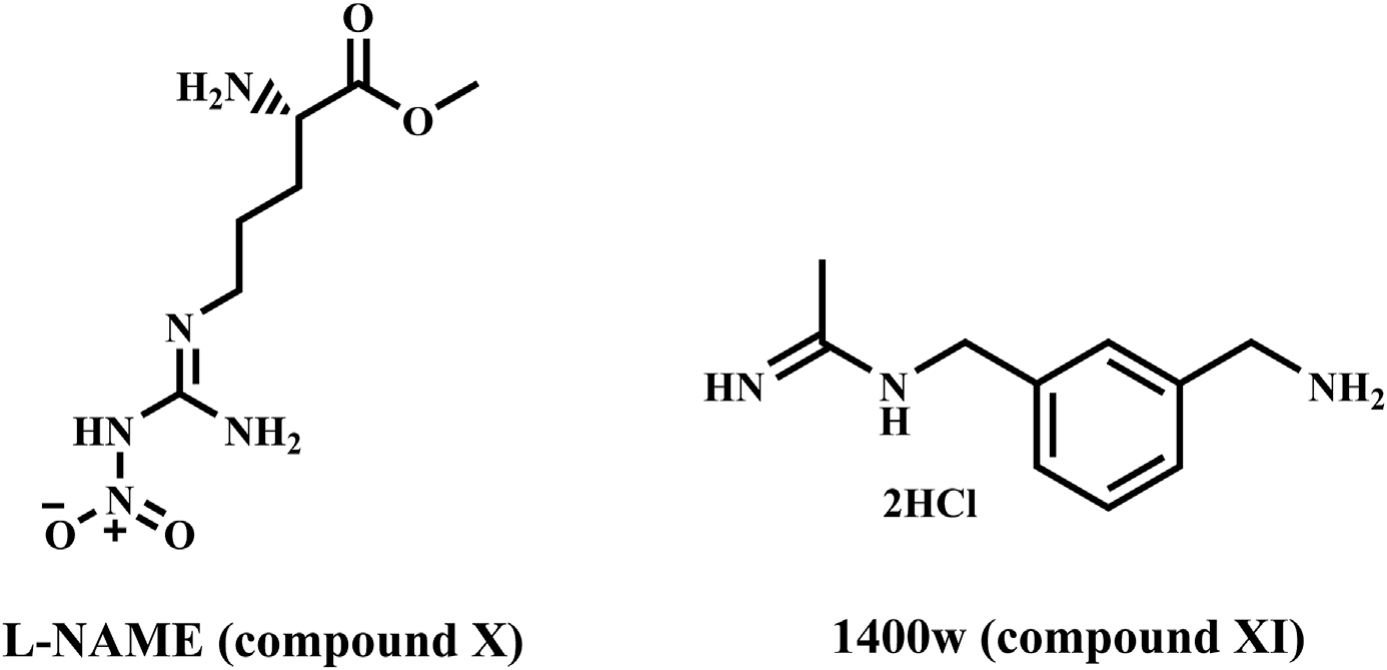
Structures of compounds **X** and **XI**.

On the other hand, nitric oxide (NO) is a small biomolecule that has various effects on tumor biology (Pervin et al., 2007). In general, depending on the tumor microenvironment and concentration, NO can influence cell proliferation, migration, and apoptosis (Burke et al., 2013; Shami et al., 1998). Endothelial (eNOS) and neuronal (nNOS) are two constitutive NOS isoforms; eNOS is crucial for maintaining vascular homeostasis, and its uncoupling is linked to the emergence of cardiovascular disorders (Pourbagher-Shahri et al., 2021). Conversely, the nNOS is primarily linked to learning and memory, smooth muscle relaxation, and synaptic plasticity (Wang et al., 1999). Aside from the constitutive NOS isoforms, an inducible NOS isoform (iNOS) is expressed in response to pro-inflammatory stimuli and is thus vital in the immune system. However, iNOS can be overexpressed in a variety of pathological conditions that result in uncontrolled NO production, including inflammatory bowel disease, rheumatoid arthritis, and cancer; thus, iNOS inhibition could be a therapeutic strategy for these diseases (Cinelli et al., 2020; Maccallini et al., 2020). In particular, iNOS overexpression is thought to be a predictor of poor outcomes in TNBC (Triple Negative Breast Cancer) patients, as it is associated with decreased relapse-free survival (Glynn et al., 2010), and iNOS can modulate many potential therapeutic targets for TNBC (Walsh et al., 2016). TNBC proliferation and migration were suppressed *in vitro* and *in vivo* by iNOS inhibition using L-NAME **(compound X)** and 1400 W **(compound XI)** (phase 1/2 clinical trials, Figure 3) (Granados-Principal et al., 2015). Furthermore, iNOS regulates tumor progression in an inflammatory environment by modulating the EGFR/MAPK pathway (Garrido et al., 2017). As a result, safer iNOS inhibitors with improved pharmacokinetic properties are desired to obtain new valuable therapeutic tools for treating breast cancer.

Additionally, apoptosis (programmed cell death) is a crucial control mechanism that triggers cell death when DNA damage surpasses the capability of the repair processes (Denmeade and Isaacs, 1996). Apoptosis, which is a normal factor of development, helps to control cell number and proliferation. Defects in apoptotic signalling contribute to several human disorders, including cancer. These flaws allow tumor cells to live longer than expected, reducing their reliance on exogenous survival factors and shielding them from oxidative stress and hypoxia, resulting in tumor growth and expansion (Cotter, 2009). These flaws allow for the accumulation of genetic changes that promote angiogenesis, disrupt cell proliferation, interfere with differentiation, and increase invasiveness during tumor formation. Therefore, restoring normal apoptotic equilibrium is a reliable practice for cancer treatment (Plati et al., 2008).

**FIGURE 4:**
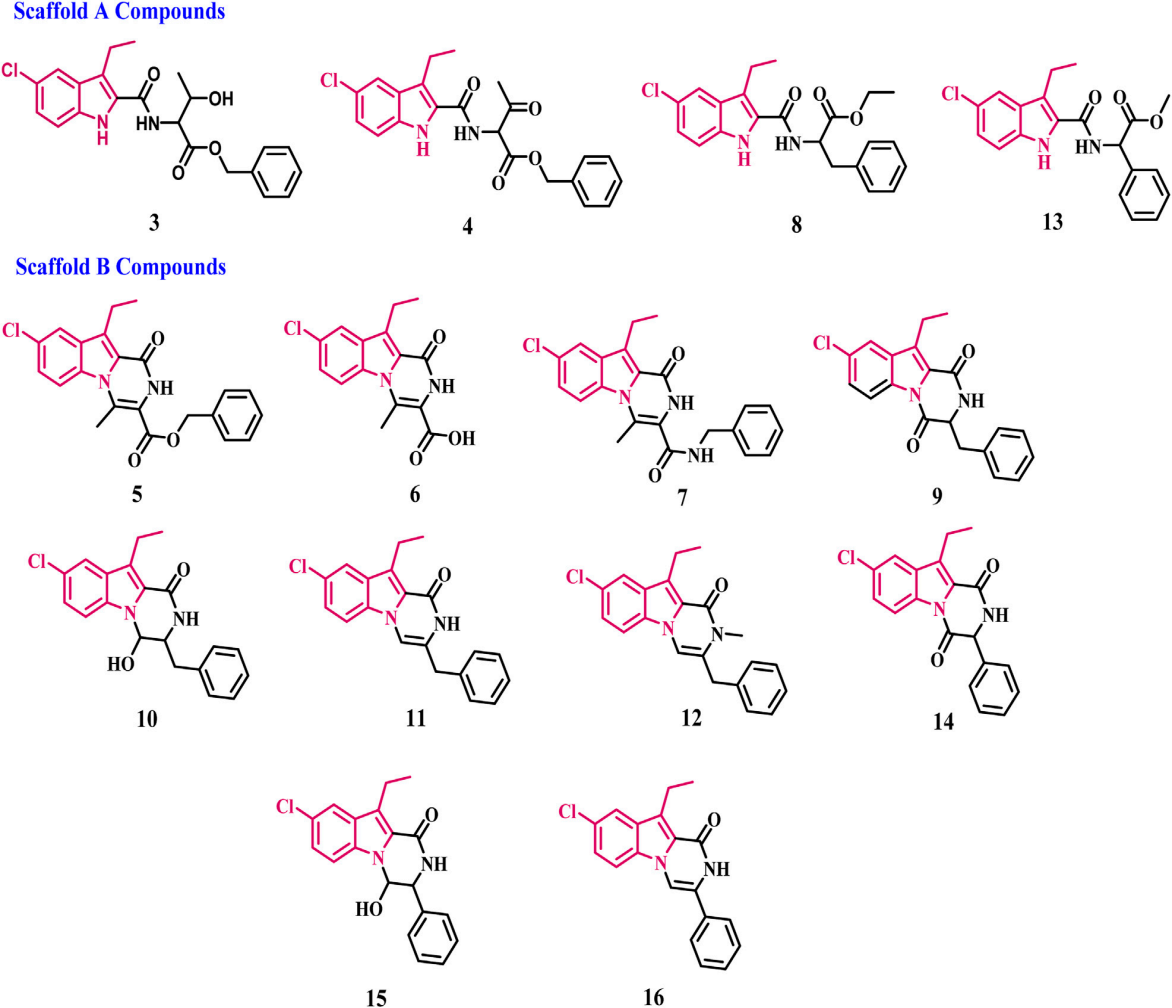
Structures of new compounds **3–16**.

In our ongoing search for anticancer and chemopreventive agents (Al-Wahaibi et al., 2020; Al-Wahaibi et al., 2023a; Al-Wahaibi et al., 2023b; Al-Wahaibi et al., 2023c; Gomaa et al., 2022), we synthesized and tested a series of indole-based scaffold derivatives **(3–16)** against aromatase and iNOS. The newly synthesized compounds fall into two categories (Figure 4): scaffold A compounds that are indole-2-carboxamide derivatives **(3, 4, 8, and 13)** and scaffold B compounds that are pyrazino [1,2-a]indol-1(2*H*)-ones **(5–7, 9, 10–12, and 14–16)**. The newly synthesized compounds will be tested *in vitro* against a panel of four cancer cell lines as antiproliferative agents. All the newly synthesized compounds were then tested for aromatase inhibitory activity. The most potent aromatase inhibitors will be tested for their ability to inhibit iNOS. Moreover, the apoptotic potential of the most active derivatives was evaluated. Finally, docking analysis and ADME experiments were conducted for the most potent derivatives.

## 2 Results and discussion

### 2.1 Chemistry

**SCHEME 1:**
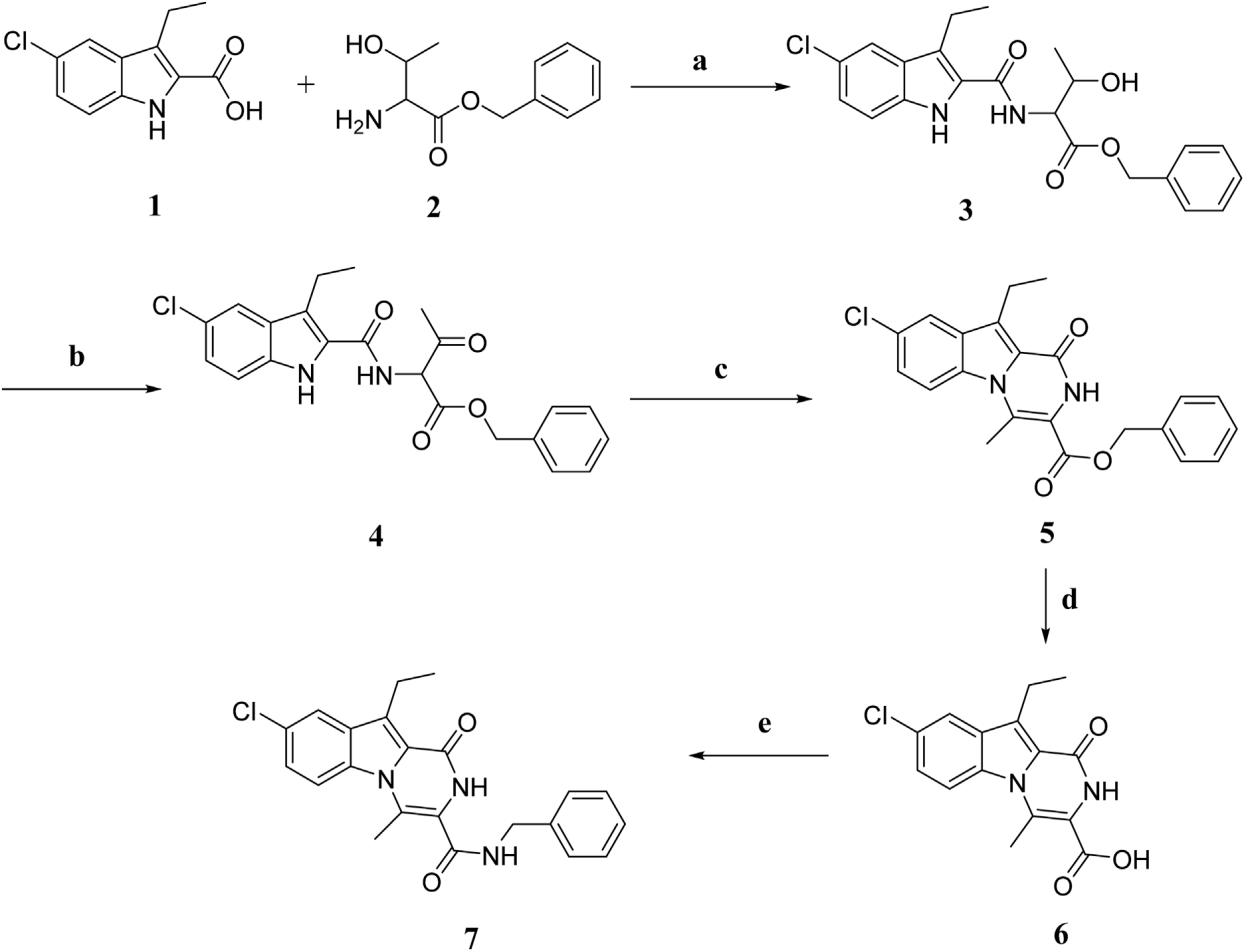
Synthesis of compounds **3–7**. Regents and reaction conditions: **(A)** POB, DIPEA, r. t., overnight, 91%; **(B)** Dess-Martin periodinane, DCM, r. t. 5h; **(C)** PTAS, Toluene, reflux overnight, 82%; **(D)** 5% NaOH, 45°C, overnight, 96%; **(E)** benzylamine, POB, DIPEA, r. t. overnight, 81%.

Scheme 1 depicts the synthesis of the key intermediates and target compounds **3–7**. Compound **1** was reacted with L-threonine benzyl ester **2** in the presence of BOP and DIPEA as coupling reagents, yielding compound **3** as a white solid after chromatography on silica gel with EtOAc/hexane (2:3) as an eluent. The oxidation of compound **3** with Dess-Martin periodinane reagent produced a crude product **4** that was used in the following step without further purification. Refluxing **4** in toluene with PTSA yields crude product purified by flash chromatography using EtOAc and n-hexane to afford **5** in 82% yield as a white solid. The structure of **5** was validated using ^1^H NMR, ^13^C NMR, and HRMS spectroscopy. The ^1^H NMR spectrum of **5** revealed the pyrazine NH characteristic signal in the form of a singlet of one proton at δ 8.41 ppm corresponding to NH and a singlet signal of benzyl methylene protons at δ 5.36 ppm (CH_2_Ph). The spectrum also revealed a singlet signal of 3H at δ 3.19 ppm, corresponding to the CH_3_ group, as well as protons from the ethyl group in the form of a quartet signal (2H) at δ 3.30 ppm and a triplet signal (3H) at δ 1.30 ppm. The ^13^C NMR spectrum of **5** revealed two signals at δ 162 and δ 156 ppm for (ester C=O) and (NH-C=O), respectively, and aromatic carbons. The **5** structure was also validated by HRESI-MS with the [M + H]^+^ ion at m/z 393.1013. Compound **5** was hydrolysed with aqueous NaOH to yield carboxylic acid **6**. Carboxamide **7** was synthesized by coupling carboxylic acid **6** with benzylamine in the presence of DIPEA in DCM using BOP as the coupling reagent.

Compounds **6** and **7**’s structures were confirmed using ^1^H NMR, ^13^C NMR, and HRESI-MS spectroscopy. Compound **7**’s ^1^H NMR spectrum revealed the presence of two singlet signals, one at 10.74 ppm of pyrazine NH and the other at δ 9.05 ppm of NHCH_2_Ph, the characteristic signals of ethyl group in the form of a quartet at δ 3.25 ppm (2H) and triplet at δ 1.25 ppm (3H), and a singlet signal of CH_3_ group at δ 2.73 ppm (3H). Additionally, the spectrum revealed the presence of aromatic proton-specific signals. HRESI-MS m/z of **7** calcd for [M-H]^-^ C_22_H_19_ClN_3_O_2_: 392.1171, found: 392.1168.

**SCHEME 2:**
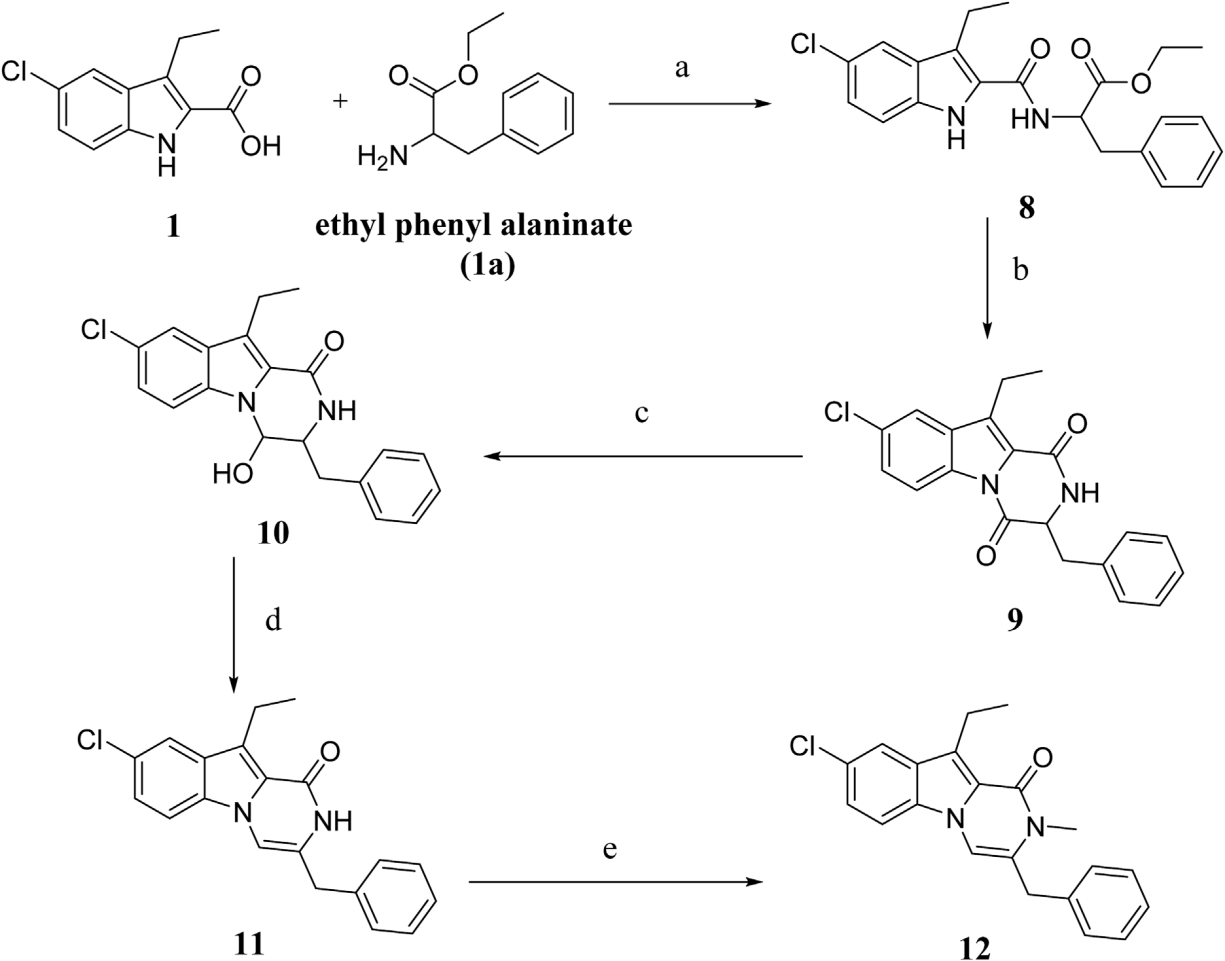
Synthesis of compounds **8–12** Regents and reaction conditions: **(A)** POB, DIPEA, r. t., overnight, 83%; **(B)** PTAS, Toluene, reflux overnight, 75%; **(C)** NaBH_4_, aq. EtOH, r. t. 1h, 93%; **(D)** PTAS, Toluene, reflux 4 h, 60%; **(E)** CH_3_I, NaI, NaH, DMF, r. t. overnight, 88%.

The synthesis of compounds **8–12** is outlined in Scheme 2. Compound **8** was synthesized using compound **1** and ethyl phenylalanine **(compound 1a)** as described in the general procedure for the synthesis of compound **3** (Scheme 1), yielding **8** as an oily product after purification by flash chromatography on silica gel short column using EtOA and n-hexane. Compound **9** is obtained by cyclizing **8** in toluene with PTSA. It is then purified using flash chromatography with EtOAc and n-hexane to yield **9** as a white solid in 75% yield. Various spectroscopic analysis methods were used to confirm the structure of compound **9**. The ^1^H NMR spectrum **9** revealed a pyrazine NH characteristic signal at δ 8.31 ppm and a methine proton multiplet signal at δ 4.81–4.79 ppm (NHCH). The spectrum also revealed two doublet signals of one proton each at δ 3.29 and 3.10 ppm, corresponding to the CH_2_Ph group and ethyl and aromatic protons. The ^13^C NMR spectrum of **9** revealed two signals for (CHC = O) and (NH-C = O), respectively, at δ 165.56 and δ 157.82 ppm and aromatic carbons. Compound **10** was obtained in quantitative yield by reducing **9** with NaBH_4_ in aqueous ethanol. The ^1^H NMR spectrum of **10** revealed the presence of a doublet signal at δ 7.69 (1H) corresponding to CHOH and a singlet signal at δ 4.89 of the hydroxyl group (CHOH). Moreover, the ^13^C NMR revealed the presence of only one carbonyl carbon signal at δ 161.50 of (NH-C=O). HRESI-MS m/z of **10** calcd for [M + H]^+^ C_20_H_20_ClN_2_O_2_: 355.1208, found: 355.1210 validated compound **10**. Compound **12** was synthesized from **10** in two steps: dehydration with PTSA in toluene to yield **11**, then alkylation with MeI in DMF in the presence of NaI to afford **12** in 88% yield as a white solid. The compound **12** ^1^H NMR spectrum revealed a singlet signal at δ 3.86 (2H, PhCH_2_), while the CH_3_ group signal overlapped with that of the ethyl group signal as a multiplet signal at δ 3.26–3.19 (5H, CH_3_, CH_2_CH_3_) and a triplet signal at δ 1.25 (3H, CH_2_CH_3_). HRESI-MS with the [M + H]^+^ ion at m/z 351.1257 also validated the **12** structure.

**SCHEME 3:**
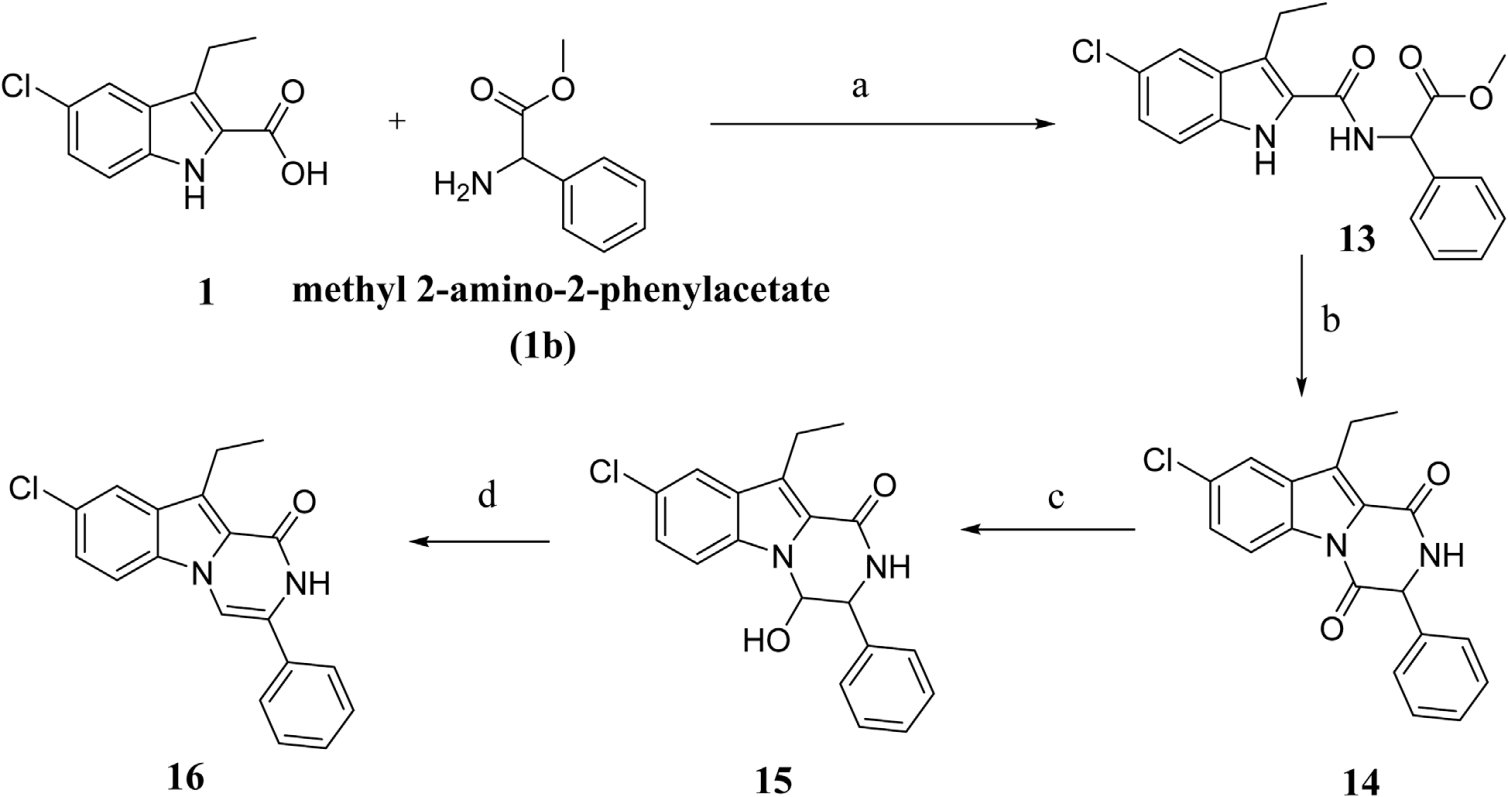
Synthesis of compounds **13–16** Regents and reaction conditions: **(A)** POB, DIPEA, r. t., overnight, 94%; **(B)** PTAS, Toluene, reflux overnight, 85%; **(C)** NaBH_4_, aq. EtOH, r. t. 1h, 89%; **(D)** PTAS, Toluene, reflux 4 h, 88%.

Scheme 3 depicts the synthesis of compounds **13–16**. Compound **13** was synthesized from compound **1** and methyl-2-amino-2-phenylacetate **(compound 1b)** using the typical process for compound **8** synthesis (Scheme 2), producing **13** as a solid product. By cyclizing **13** in toluene with PTSA, compound **14** is obtained as a white solid. Compound **15** was obtained in 89% yield by reducing **14** in aqueous ethanol with NaBH_4_, followed by dehydration with PTSA in toluene to get **16**. Various spectroscopic methods of analyses were employed to confirm the structures of compounds **13–16** (Supplementary Material).

### 2.2 Biology

#### 2.2.1 Cell viability assay

This test investigates the effects of the new compounds **3–16** on normal cell lines to determine their safety. The normal human mammary gland epithelial (MCF-10 A) cell line was used to test the viability of the tested compounds. After 4 days of incubation on MCF-10 A cells within 50 µM of each investigated compound, cell viability was determined using the MTT assay (Gomaa et al., 2020; Marzouk et al., 2020). Table 1 outcomes demonstrate that none of the compounds examined were cytotoxic, and all compounds showed more than 89% cell viability at 50 µM.

**TABLE 1:** Cell viability assay and antiproliferative action of compounds **3–16**.

Comp	Cell viability %	Antiproliferative activity IC_50_ ± SEM (nM)				
		A-549	MCF-7	Panc-1	HT-29	Average IC_50_ (GI_50_)
3	91	57 ± 5	62 ± 5	58 ± 5	60 ± 5	59
4	89	59 ± 5	65 ± 6	60 ± 5	64 ± 6	62
5	90	31 ± 3	34 ± 3	32 ± 3	32 ± 3	32
6	92	65 ± 6	68 ± 6	64 ± 6	66 ± 6	66
7	88	28 ± 2	30 ± 3	28 ± 2	29 ± 2	29
8	92	37 ± 3	40 ± 3	37 ± 3	38 ± 3	38
9	91	48 ± 4	50 ± 4	47 ± 4	50 ± 5	49
10	89	53 ± 5	56 ± 5	54 ± 5	54 ± 5	54
11	92	40 ± 4	43 ± 4	40 ± 4	42 ± 4	41
12	89	23 ± 2	25 ± 2	24 ± 2	25 ± 2	24
13	91	34 ± 3	36 ± 3	35 ± 3	34 ± 3	35
14	90	44 ± 4	46 ± 4	44 ± 4	46 ± 4	45
15	92	69 ± 6	76 ± 7	72 ± 7	74 ± 7	72
16	91	25 ± 2	28 ± 2	25 ± 2	25 ± 2	26
Erlotinib	ND	30 ± 3	40 ± 3	30 ± 3	30 ± 3	33

#### 2.2.2 Antiproliferative assay

The MTT assay was used to investigate the antiproliferative activity of new compounds **3–16** *versus* four human cancer cell lines using erlotinib as a control: colon (HT-29), pancreatic (Panc-1), lung (A-549), and breast (MCF-7) cancer cell lines (Youssif et al., 2022; Mahmoud et al., 2022). Table 1 displays the median inhibitory concentration (IC_50_) and GI_50_ (average IC_50_) against the four cancer cell lines.

Generally, the investigated compounds **3–16** had significant antiproliferative activity with GI_50_ values ranging from 24 nM to 72 nM *versus* the four cancer cell lines evaluated, compared to the standard Erlotinib, which had a GI_50_ value of 33 nM. Compounds **5**, **7**, **8**, **12**, **13**, and **16** were the most potent six derivatives, with GI_50_ values ranging from 24 nM to 38 nM, making compounds **5**, **7**, **12**, and **16** (GI_50_ = 32, 29, 24, and 26, respectively) more potent than Erlotinib (GI_50_ = 33 nM), and even more potent than Erlotinib against breast (MCF-7) cancer cell lines. Compound **12** (R = 3-benzyl) was the most potent of the newly synthesized derivatives **3–16**, with a GI_50_ value of 24 nM, 1.4-fold higher than the reference Erlotinib (GI_50_ = 33 nM). Based on the results, the type of substitution detected on the nitrogen atom at position two of the pyrazino [1,2-a]indol-1(2*H*)-one moiety appears to be crucial for activity. Compound **11** (R = 3-benzyl), which has the same backbone as compound **12** but with a free NH group at position **2**, displayed a GI_50_ of 41 nM (1.7-fold less potent than **12**), demonstrating that the N-methyl group at position two is important for the antiproliferative activity.

Moreover, the 3-position substitution of the pyrazino [1,2-a]indol-1(2*H*)-one moiety is critical for activity. Compounds **7** (R = 3-benzylcarboxamide) and **16** (R = 3-phenyl) had GI_50_ values of 29 nM and 26 nM, respectively, indicating they were at least 1.4-fold more effective than compound **11**. These data indicate that the third position substitution significantly affects the antiproliferative activity of these compounds, with the highest activity shown in compounds with a benzyl substitution, followed by carboxamide and phenyl substitutions.

Another area of interest is the difference in efficacy between open-ring compounds and their closed-ring counterparts. Open-ring compounds **8** and **13** showed promising antiproliferative effects with GI_50_ values of 38 nM and 35 nM, respectively, being equipotent to the reference Erlotinib (GI_50_ = 33 nM), whereas compounds **3** and **4** showed weak activity with GI_50_ values of 59 nM and 62 nM, respectively. Based on these findings, it is possible to conclude that the kind of functional groups and the type of substituents dictate the nature of these compounds’ activity. Finally, Compounds **6**, **10**, and **15** exhibited low potency, as seen by their GI_50_ values of 66, 54, and 72 nM, respectively. This suggests that having a high level of hydrophilicity does not confer any advantage for antiproliferative activity.

#### 2.2.3 Assay for aromatase inhibitory action

Using ketoconazole and letrozole as reference medications, compounds **5**, **7**, **8**, **12**, **13**, and **16** were examined for their ability to inhibit aromatase, a possible target for their antiproliferative effect (Maghraby et al., 2023). Table 2 displays the results as IC_50_ values. The examined compounds **5**, **7**, **8**, **12**, **13**, and **16** displayed significant aromatase inhibitory effect with IC_50_ values ranging from 10 nM to 33 nM, in comparison to the reference drugs ketoconazole (IC_50_ > 100 nM) and letrozole (IC_50_ = 2 nM). The assay’s results align with the findings of the antiproliferative assay, which demonstrated that compounds **7**, **12**, and **16**, the most potent antiproliferative derivatives, were also the most potent aromatase inhibitors, with IC_50_ values of 15 nM, 10 nM, and 12 nM, respectively.

**TABLE 2:** Anti-aromatase activity of compounds **5**, **7**, **8**, **12**, **13**, and **16**.

Comp	IC_50_ ± SEM (nM)
5	20 ± 2
7	15 ± 1
8	33 ± 3
12	10 ± 1
13	28 ± 2
16	12 ± 1
Ketoconazole	>100
Letrozole	2 ± 0.20

Once again, compound **12** was the most potent aromatase inhibitor with an IC_50_ value of 10 ± 1 nM, at least 10-fold more potent than the standard Ketoconazole but 5-fold less potent than the Letrozole. Compound **16** was the second most active aromatase inhibitor, with an IC_50_ value of 12 ± 1 nM, like compound **12**. Finally, compounds **5**, **8**, and **13** demonstrated good inhibitory action with IC_50_ values of 20 ± 2, 33 ± 3, and 28 ± 2 nM, respectively. These findings indicate that antiproliferative and anti-aromatase activity are linked and that any parameter that affects one has the same effect on the other.

#### 2.2.4 Nitric oxide synthase inhibition

The L-citrulline assay method with fluorometric detection was used to test compounds **5**, **7**, **12**, and **16** (most potent anti-aromatase derivatives) for their ability to inhibit iNOS (Carrión et al., 2023). The investigated compounds were tested at 1 µM against the iNOS and 10 µM against the eNOS to determine their isoform selectivity. Because of its critical role in cardiovasculature, inhibiting the constitutive enzyme eNOS should be avoided. Table 3 shows the results, which were expressed as enzyme percent inhibition compared to 1400 W as the positive control (100% inhibition at 10 µM).

**TABLE 3:** Enzyme percent inhibition of compounds **5**, **7**, **12**, and **16** against iNOS and eNOS.

Comp	Inhibition (%)	
	iNOS	eNOS
5	62 ± 2	49 ± 2
7	73 ± 2	32 ± 2
12	87 ± 3	--
16	81 ± 3	--

Results revealed that compounds **7**, **12**, and **16** were effective iNOS inhibitors, with enzyme percent inhibition values of 73, 87, and 81, respectively. Compound **7** had moderate inhibitory action against eNOS isoform, whereas compounds **12** and **16** were completely inactive against eNOS. Moreover, compound **5** was active against eNOS with a percent % inhibition value of 49%, even though it maintained a good inhibition of iNOS (62%), Table 3.

As a result, compounds **12** and **16** were chosen for IC_50_ evaluation (Table 4). Interestingly, derivative **12** had an IC_50_ value against iNOS of 0.075 µM, compared to reference 1,400 W (0.082 µM), as well as excellent isoform selectivity against eNOS (>670). Additionally, despite being less potent than reference 1400W, compound **16** demonstrated its efficacy against iNOS (IC_50_ = 0.098 µM) while being inactive against eNOS. Based on their inhibition potency, derivatives **12** and **16** were thought to be possible aromatase inhibitors with iNOS inhibitory action.

**TABLE 4:** IC_50_ values of compounds **12** and **16** against iNOS and eNOS.

Comp	IC_50_ (µM)		eNOS/iNOS selectivity
	iNOS	eNOS	
12	0.075 ± 0.002	>50	>670
16	0.098 ± 0.003	>50	>510
1400 W	0.082 ± 0.002	>50	>610

#### 2.2.5 Apoptosis assays

Compounds **12** and **16**, the most potent derivatives in all *in vitro* investigations, were examined for their ability to initiate the apoptosis cascade and demonstrate proapoptotic activity.

##### 2.2.5.1 Caspase-3 and caspase-8 expression levels

When cells get specific signal instructions, they undergo apoptosis, which results in several significant changes. Caspases, considered the main workers in apoptosis, are activated early in the process, cleaving critical cellular components such as nuclear proteins like DNA repair enzymes or structural proteins found in the cytoskeleton that are required for optimal cellular function. Caspases can activate DNases, which break down nuclear DNA (Chauhan et al., 2001; Kroemer and Reed, 2000). Compounds **12** and **16** were tested as caspase-3/8 activators against the human epithelial (A-594) cancer cell line (Youssif et al., 2019), with the results presented in Table 5.

**TABLE 5:** Apoptotic markers assays of compounds **12** and **16**.

Compd. No.	Caspase-3		Caspase-8		Bax		Bcl-2	
	Conc (Pg/ml)	Fold change	Conc (ng/ml)	Fold change	Conc (Pg/ml)	Fold change	Conc (ng/ml)	Fold reduction
12	580 ± 5	9.0	2.60 ± 0.20	29	368 ± 3	41	0.65	8
16	530 ± 5	8.0	2.25 ± 0.18	25	315 ± 3	35	0.85	6
Staurosporine	465 ± 4	7.0	1.85 ± 0.15	21	288 ± 2	32	1.00	5
Control	65	1.0	0.09	1	9	1	5.00	1

**FIGURE 5:**
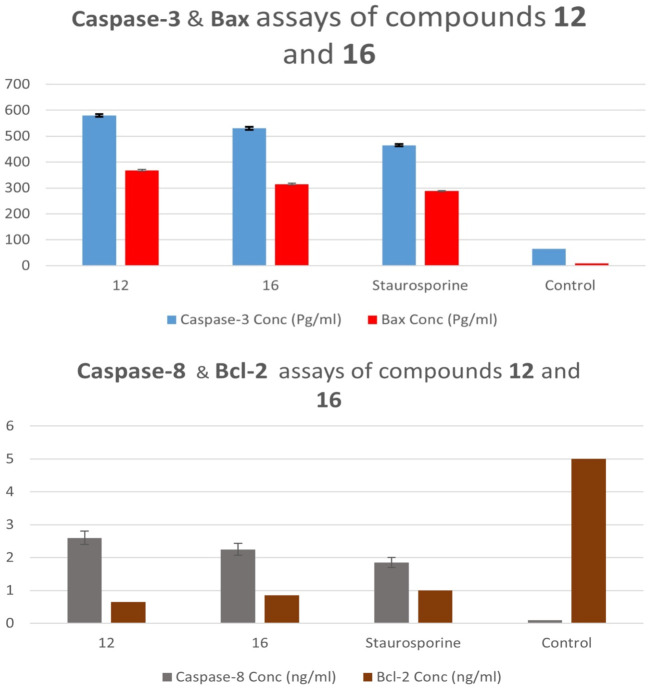
Caspase 3, 8, Bax, and Bcl-2 expression levels of compounds **12** and **16**.

In comparison to the untreated control cells, compound **12** treatment at its IC_50_ concentration increased the expression levels of active caspase-3 and caspase-8 in A-594 cells by 9 and 29 folds, respectively (Table 5; Figure 5). Compound **16** treatment causes induction of caspase-3 and caspase-8 levels to be 8 and 25 times greater than those of untreated control cells. In all cases, compounds **12** and **16** were more effective caspase-3 and 8 activators than the control Staurosporine.

##### 2.2.5.2 Proapoptotic BAX and anti-apoptotic Bcl-2 expression levels

Tumor cells can develop resistance to apoptosis through mutation or downregulation of proapoptotic proteins (e.g., Bax and Bak) or over-expression of anti-apoptotic proteins (e.g., Bcl-2 and Bcl-xL), which promote cell survival and are considered a hallmark in more than half of all cancers. This makes cancer cells more resistant to apoptotic stimuli and conventional cytotoxic anticancer drugs (Miyashita et al., 1994).

In the current study, treating lung (A-549) cancer cell lines with compounds **12** and **16** at IC_50_ values increased pro-apoptotic Bax expression levels by 41 and 35 folds, respectively, while decreasing anti-apoptotic Bcl-2 expression levels by approximately 8 and 6 folds, Table 5 and Figure 5. As a result, Compounds **12** and **16** significantly increased the Bax/Bcl-2 ratio compared to the untreated control cells.

### 2.3 Docking study into aromatase

This research conducted a computational docking analysis to explore the binding interactions of the most active derivatives **7, 12**, and **16** with the human placental aromatase cytochrome P450 (CYP19A1). Aromatase, a crucial enzyme in estrogen biosynthesis, belongs to the cytochrome P450 family and features a heme group at its catalytic centre, essential for its enzymatic activity. The active site is located within a deep hydrophobic pocket, surrounded by key residues such as Met374, Arg115, Ala306, Val370, and Val373, which are critical for substrate binding and catalysis. These residues not only stabilize the binding of substrates and inhibitors but also play a significant role in the enzyme’s selectivity and function. The careful arrangement of these residues around the heme group ensures precise catalytic activity, making aromatase a pivotal target in breast cancer therapy. The Discovery Studio software was used for this study, combined with the crystallographic structure of human placental aromatase cytochrome P450, which was co-crystallized with testosterone (PDB: 5JKW) (Ibrahim et al., 2020; Shaykoon et al., 2020; Bhat et al., 2022).

**FIGURE 6:**
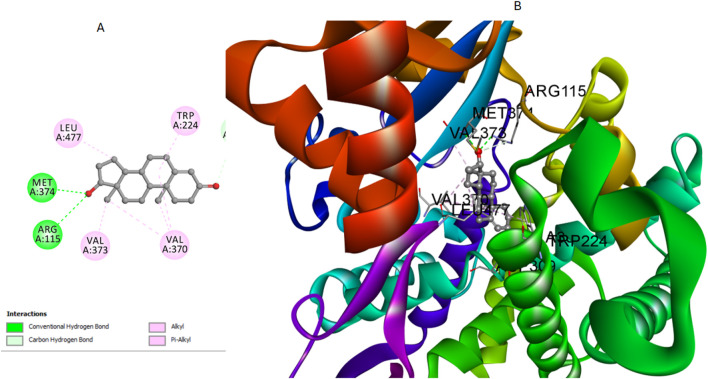
Cartoon and surface representations of the aromatase binding pocket docked with testosterone (PDB: 5JKW). The model shows the hydrogen bond interaction between the hydroxyl group of testosterones and the amino acids Met374 and Arg115, with additional interactions with Ala306, Val370, and Val373.

The molecular systems underwent energy minimization using the OPLS-AA force field, a method essential for ensuring conformational stability (Bernacki et al., 2005). Before starting the docking experiment, careful preparation of the protein structure was carried out to enhance accuracy. This included protonation steps, which significantly improved the reliability of the following docking analysis. The effectiveness of the docking process was validated by re-docking the co-crystallized testosterone into the active site of the aromatase enzyme, which yielded an S score of −8.43 kcal/mol. The S score is a measure used to assess the binding strength between compounds and their target protein, with lower scores indicating stronger binding affinities. Notably, this result was marked by a crucial hydrogen bond interaction between the hydroxyl group of testosterone and the amino acids Met374 and Arg115. Additionally, there were multiple interactions with Ala306, Val370, and Val373, highlighting their importance in ligand stabilization (Figure 6).

To further illustrate the binding interactions, cartoon and surface representations of the aromatase binding pocket are provided, highlighting the spatial arrangement of these critical residues and their interaction with the docked ligands. These visual models help in understanding how the structural features of the protein contribute to its interaction with different ligands (Figure 6).

The analysis of docking scores revealed that hybrid **12**, which had the highest *in vitro* aromatase activity, showed a score of −7.86 kcal/mol. In contrast, hybrid **7**, with the lowest *in vitro* aromatase activity, registered a score of −6.95 kcal/mol. Additionally, the docking score for compound **16,** when tested against aromatase, was determined to be −7.55 kcal/mol.

**FIGURE 7:**
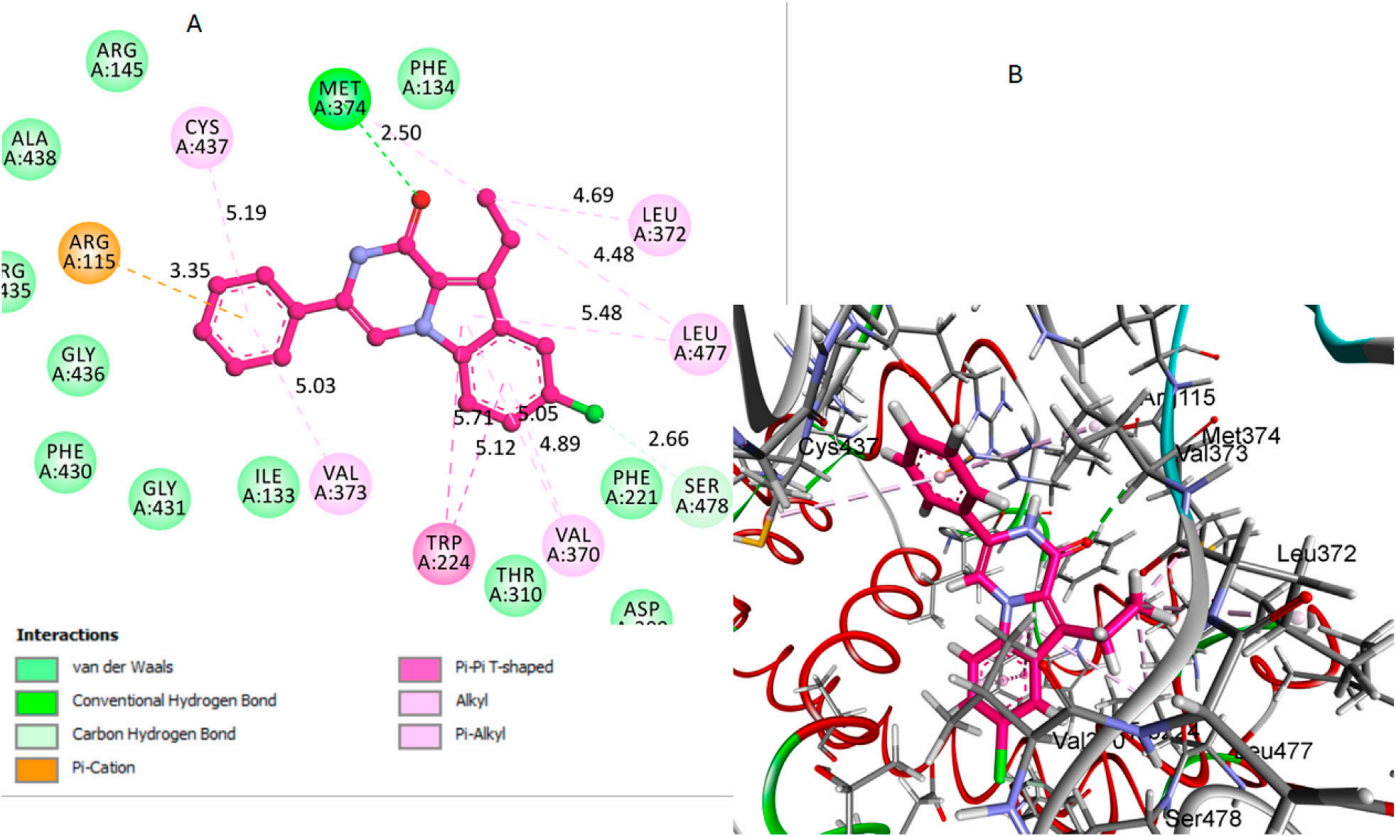
Docking representation models of compound **12** within the binding site of aromatase (PDB: 5JKW) **(A)** 2D-docked model of compound **12 (B)** 3D-docked model of compound **12**. The carbonyl oxygen of pyrazine forms a hydrogen bond with Met374, and aromatic rings engage in hydrophobic interactions with Val370, Leu372, and Leu477.

Regarding the interactions between the compounds and the aromatase enzyme, compound **12** demonstrated significant interactions. Hydrogen bonds are fundamental in stabilizing the ligand within the active site of the enzyme, and their strength is largely dependent on both the bond distance and the bond angle. The carbonyl oxygen in its pyrazine ring acted as a hydrogen bond acceptor, forming a bond with crucial Met374 residue. The hydrogen bond distance between the carbonyl oxygen of compound 12 and the hydrogen of the Met374 side chain was measured at 2.50 Å. The bond angle formed was calculated to be approximately 167°, indicating a nearly linear and therefore strong hydrogen bond, which is optimal for interaction stability. Additionally, the aromatic rings of compound 12 were involved in several hydrophobic interactions with key residues in the binding pocket. Notably, these interactions included pi-pi T-shaped interactions with Phe134 and alkyl/pi-alkyl interactions with Val370, Leu372, and Leu477. These non-covalent interactions collectively enhance the stability and binding affinity of compound 12 within the aromatase active site, Figure 7. The combination of these different interactions played a key role in the strong binding affinity of compound **12** within the active site of the aromatase enzyme.

**FIGURE 8:**
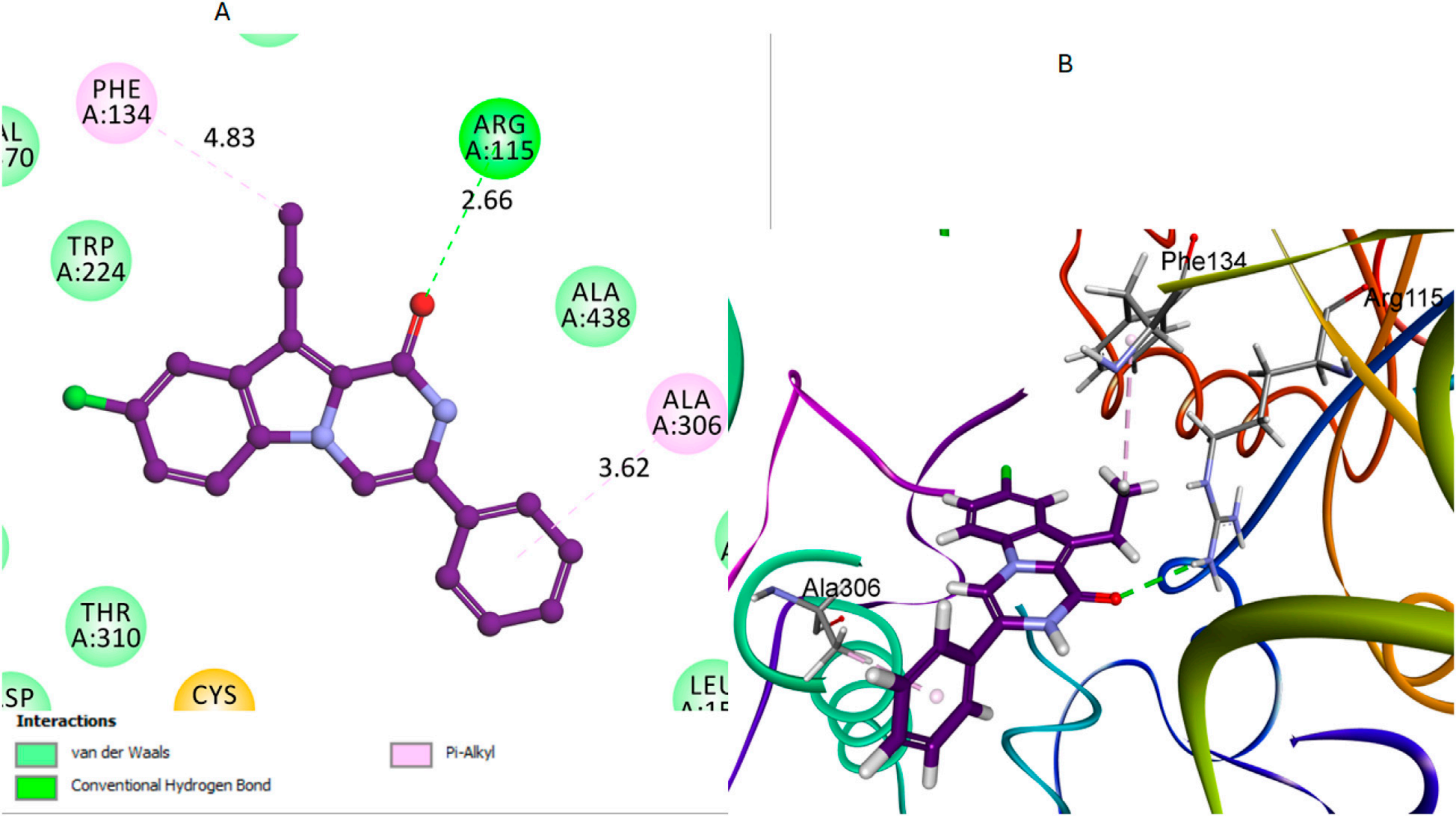
Docking representation models of compound **16** within the binding site of aromatase (PDB: 5JKW) **(A)** 2D-docked model of compound 16 **(B)** 3D-docked model of compound **16**. The carbonyl oxygen of pyrazine forms a hydrogen bond with Arg115, and the aliphatic methyl group interacts with Phe134.

Compound **16**, like compound **12**, showed significant interactions with the aromatase enzyme. In compound **16**, the carbonyl oxygen of the pyrazine moiety acted as a hydrogen bond acceptor with the Arg115 residue. This hydrogen bond plays a crucial role in anchoring compound 16 within the active site. The hydrogen bond distance between the carbonyl oxygen of compound 16 and the hydrogen of the Arg115 side chain was measured at 2.66 Å. The bond angle formed was approximately 170°, which indicates a strong and nearly optimal hydrogen bond for stabilizing the compound within the active site. Additionally, its aliphatic methyl group was involved in a pi-alkyl bond interaction with the Phe134 residue, as illustrated in Figure 8. These interactions will likely play a crucial role in stabilizing the compound within the binding pocket of the aromatase enzyme.

**FIGURE 9:**
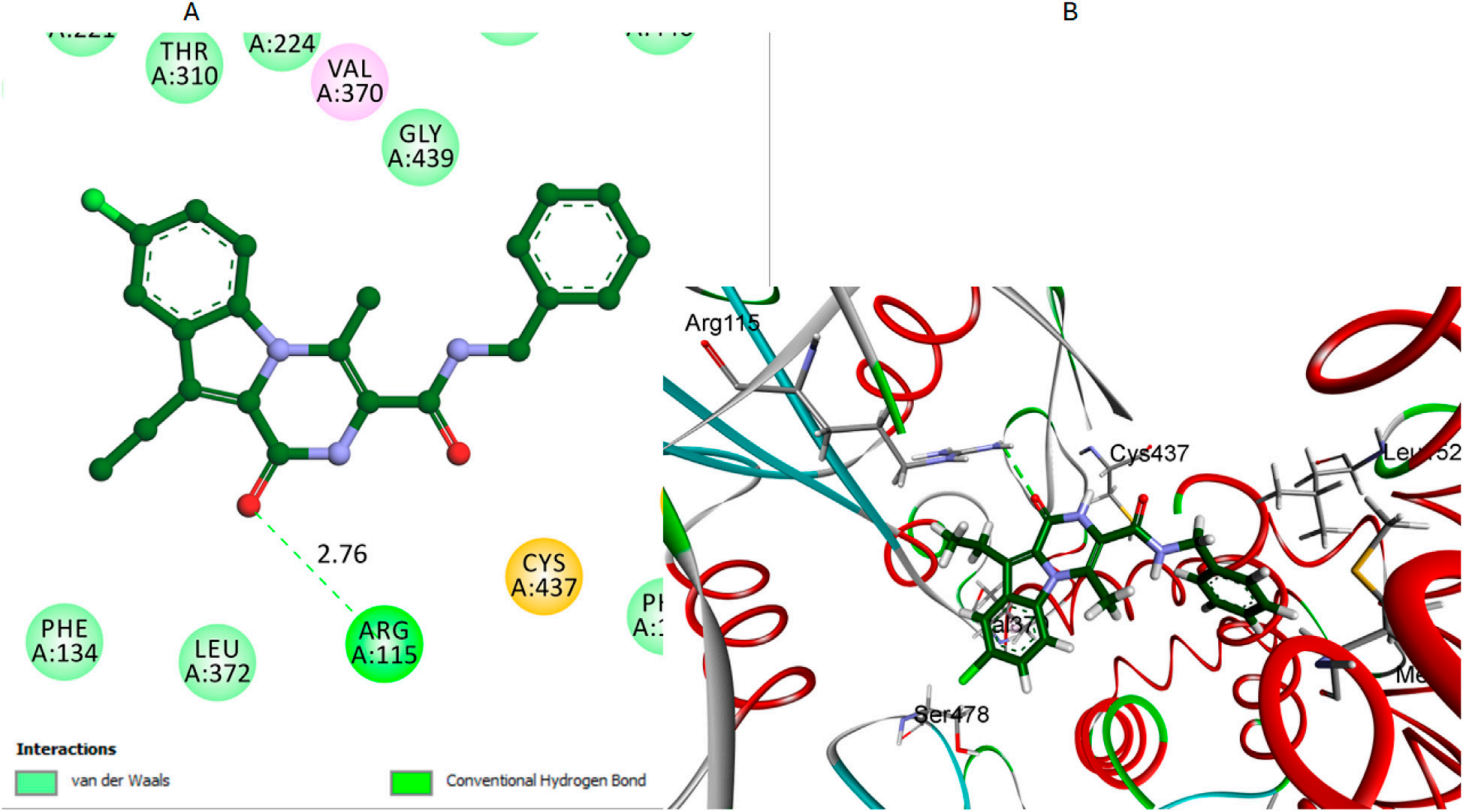
Docking representation models of compound **7** within the binding site of aromatase (PDB: 5JKW) **(A)** 2D-docked model of compound **7 (B)** 3D-docked model of compound **7**. The carbonyl oxygen of pyrazine establishes a hydrogen bond with Arg115.

Similarly, pyrazoline oxygen of compound **7** establishes a hydrogen bond with the crucial residue Arg115 when it interacts with the aromatase enzyme, Figure 9. This hydrogen bond is significant as it represents the primary interaction stabilizing compound 7 within the active site. The hydrogen bond distance between the carbonyl oxygen of compound 7 and the hydrogen of the Arg115 side chain was measured at 2.76 Å. The bond angle was approximately 160°, which supports a moderately strong hydrogen bond for stabilizing the compound within the active site. However, this is the only significant interaction observed for compound **7**. The length of its amide bridge may not be optimal for a snug fit in the aromatase active site. Compound **7** also lacks the extensive hydrophobic interactions in compounds **12** and **16**.

In conclusion, these results offer a basic insight into the potential inhibitory effects of the examined compounds and establish a foundation for further experimental validation and optimization in drug discovery. The detailed elucidation of these molecular interactions is crucial in directing the rational design of new therapeutic agents, particularly those targeting cancer-related pathways.

### 2.4 ADMET studies

**FIGURE 10:**
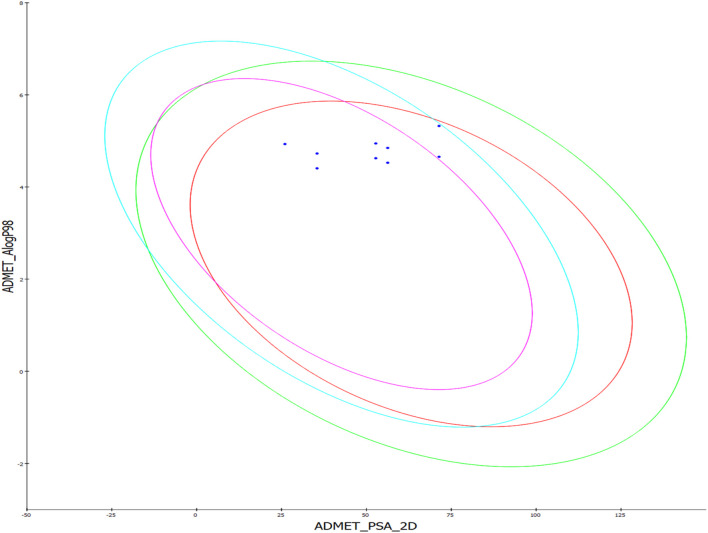
The predicted ADME study of hybrids **8–16**. Comprehensive profiles of absorption, distribution, metabolism, and excretion properties are depicted, including plasma protein binding, blood-brain barrier permeability, CYP2D6 inhibition, solubility, and lipophilicity (AlogP98).

This supplementary section presents a comprehensive overview of the ADME (Absorption, Distribution, Metabolism, Excretion) studies for the newly synthesized compounds, specifically compounds **8–16**. These studies are essential to evaluate the compounds’ pharmacokinetics and potential therapeutic efficacy (Al-Wahaibi et al., 2023d). The absorption studies show that all compounds have good absorption levels but vary in their Polar Surface Area (PSA), influencing their solubility and permeability. In terms of distribution, all compounds exhibit high plasma protein binding (>90%), which is crucial for their distribution in the bloodstream and free concentration. Additionally, the Blood-Brain Barrier (BBB) permeability ranges from very high to medium, indicating potential central nervous system activity for some compounds. The metabolism analysis reveals that several compounds (**10–16**) are inhibitors of the CYP2D6 enzyme, which could lead to significant drug-drug interactions and affect drug metabolism. The excretion studies indicate that most compounds have low solubility levels, potentially impacting their excretion rates. The Lipophilicity (AlogP98) values, ranging from moderate to high, suggest implications for membrane permeability and possible bioaccumulation Table 6. Overall, these ADME profiles offer valuable insights into the pharmacokinetic behaviors of the compounds. The high plasma protein binding and variation in BBB permeability necessitate carefully evaluating their therapeutic window and CNS effects (Figure 10).

**TABLE 6:** Comprehensive prediction of synthesized hybrids’ absorption, distribution, metabolism, excretion, and toxicity (ADME) profiles **8–16**.

Comp. Id	PSA	PPB^a^	Absorption level^b^	CYP2D6 prediction^c^	BBB level^d^	Solubility level^e^	AlogP98
8	71.397	Yes	0	No	1	1	5.325
9	52.76	Yes	0	No	1	1	4.945
10	56.274	Yes	0	Yes	1	2	4.848
11	35.459	Yes	0	Yes	0	1	4.726
12	26.001	Yes	0	Yes	0	1	4.932
13	71.397	Yes	0	Yes	1	2	4.655
14	52.76	Yes	0	Yes	1	1	4.624
15	56.274	Yes	0	Yes	1	2	4.526
16	35.459	Yes	0	Yes	1	1	4.405

^**a**^ PPB, plasma protein binding, No means less than 90%, Yes means > 90%.

^b^ Absorption level, 0 = good, 1 = moderate, 2 = poor, 3 = very poor.

^c^ CYP2D6, cytochrome P2D6, Yes = inhibitor, No = non inhibitor.

^d^ BBB, level, blood–brain barrier level, 0 = very high, 1 = high, 2 = medium, 3 = low, 4 = very low.

^e^ Solubility level, 1 = very low, 2 = low, 3 = good, 4 = optimal.

The inhibition of CYP2D6 by several compounds highlights the need to assess potential drug interactions further. The solubility and lipophilicity profiles underscore the importance of optimization in future drug formulation strategies. This data is instrumental in guiding these compounds’ clinical development and safety assessments, paving the way for their potential therapeutic application.

### 2.5 Structure activity relationship (SAR)

1. Open-ring (**Scaffold A**) compounds, namely, compounds **8** and **13**, exhibited significant antiproliferative effects, comparable to the reference Erlotinib. However, compounds 3 and 4 displayed limited activity, indicating that the nature of functional groups and substituents determine the landscape of these compounds’ therapeutic effects.
2. For **Scaffold B** (closed-ring) compounds, the antiproliferative effects is determined by the following factors:
a) The carbonyl oxygen in pyrazine ring (position-1) is essential for activity. It acted as a hydrogen bond acceptor, forming a bond with crucial amino acid Arg115.
b) The type of substitution detected on the nitrogen atom at position two of the pyrazino [1,2-a]indol-1(2*H*)-one moiety appears to be crucial for activity demonstrating that the N-methyl group (R_1_ = Me) at position two is important for the antiproliferative activity.
c) The third position substitution significantly affects the antiproliferative activity of these compounds, with the highest activity shown in compounds with a benzyl substitution (R_2_ = CH_2_Ph), followed by carboxamide (R_2_ = CONHCH_2_PH) and phenyl (R_2_ = Ph) substitutions
d) A high level of hydrophilicity (R_2_ = COOH and/or R_3_ = OH) is not favoured for antiproliferative action.

## 3 Conclusion

In the present work, we report the design and synthesis of new indole-based derivatives able to inhibit the aromatase enzyme and/or iNOS isozyme. Compounds **12** and **16** were found to be the most intriguing since they are strong aromatase inhibitors with IC_50_ values of 10 and 12 nM, respectively. Moreover, they are potent and selective iNOS inhibitors (IC_50_ = 0.075 and 0.098 µM, respectively), and they were potential antiproliferative agents against the MCF-7 breast cancer cell line (IC_50_ = 25 and 28 nM, respectively). Notably, at 50 µM concentrations, **12** and **16** demonstrated no effect on non-tumor cells MCF-10A, indicating the promise tumor-cell selectivity of these derivatives. The molecular docking studies have successfully demonstrated the specific binding interactions of compounds **12** and **16**, with the aromatase active site. This detailed analysis is pivotal in understanding their mechanism of action in breast cancer therapy. Furthermore, the comprehensive assessment of the absorption, distribution, metabolism, and excretion (ADME) properties of these hybrids underscores their potential as therapeutic agents. These insights, particularly their reduced toxicity profiles, are instrumental in their candidacy for effective breast cancer treatments. Based on the findings, **12** and **16** are promising prospects for the development of new agents for cancer therapy. Moreover, additional investigation into the mechanism of action, *in vivo* carcinogenic animal models, and lead optimization remains ongoing in our lab.

## 4 Experimental

### 4.1 Chemistry

**General Details:** See Supplementary Appendix SA1.

Synthesis of Benzyl 2-(5-chloro-3-ethyl-1*H*-indol-2-carboxamido)-3-hydroxybutanoate (3).

To a stirred solution of compound **1** (0.8 g, 3.59 mmol, 1eq) in DMF (30 mL), BOP (2.07 g, 4.67 mmol, 1.3 eq), L-threonine benzyl ester **2** (1.06 g, 4.31 mmol, 1.2 eq) were added and reaction mixture was cooled to 0°C before dropwise addition of DIPEA (1.87 mL, 10.77 mmol, 3 eq). After stirring overnight at rt, the reaction mixture was diluted with EtOAc and successively washed with H_2_O. The organic layer was washed with 5% aqueous HCl, saturated NaHCO_3_, and finally with brine. The organic phase was dried over MgSO_4,_ and the solvent was removed under reduced pressure to yield 1.35 g (91%) of **3** as a white solid after chromatographed on silica gel using EtOAc/hexane (2:3) as an eluent. HRESI-MS m/z calcd for [M + H]^+^ C_22_H_24_ClN_2_O_4_: 415.1419, found: 415.1418.

#### 4.1.1 Synthesis of benzyl 2-(5-chloro-3-ethyl-1*h*-indol-2-carboxamido)-3-oxobutanoate (4)

To a solution of compound **3** (0.34 g, 0.82 mmol) in DCM (30 mL), Dess-Martin periodinane (0.45 g, 1.00 mmol) was added with stirring at 0°C for 30 min, then the temperature was warmed to rt for 5 h. A saturated solution of Na_2_S_2_O_3_ (10 mL) and NaHCO_3_ (10 mL) was added to the reaction mixture by stirring at rt for 15 min. The organic layer was separated, dried over MgSO_4,_ and evaporated under reduced pressure to yield a crude product **4,** which was used for the next step without further purification.

#### 4.1.2 Synthesis of benzyl 8-chloro-10-ethyl-4-methyl-1-oxo-1,2-dihydropyrazino [1,2-a]indole-3-carboxylate (5)

A mixture of **4** (0.85 mmol, 1equiv) and PTSA (0.33 g, 1.7 mmol, 2 eq) in toluene (40 mL) was refluxed with stirring overnight. After concentration of the solvent *in vacuo*, the residue was extracted with EtOAc, washed with a saturated solution of NaHCO_3_ brine, dried over MgSO_4_, and concentrated under reduced pressure to yield a crude product which purified by flash chromatography using EtOAc, hexanes (1:4) to yield **5** (0.3 g, 82%) as white solid. Mp 180–182°C. ^1^H NMR (400 MHz, Chloroform-*d*) δ 8.41 (s, 1H, pyrazine NH), 7.98 (d, *J* = 9.2 Hz, 1H, Ar-H), 7.77 (d, *J* = 2.1 Hz, 1H, Ar-H), 7.46–7.30 (m, 6H, Ar-H), 5.36 (s, 2H, PhCH_2_), 3.30 (q, *J* = 7.5 Hz, 2H, CH_2_CH_3_), 3.19 (s, 3H, CH_3_), 1.30 (t, *J* = 7.5 Hz, 3H, CH_2_CH_3_). ^13^C NMR (101 MHz, Chloroform-*d*) δ 161.87 (ester C=O), 155.99 (C=O), 134.77, 132.35, 130.85, 129.18, 128.86, 128.82, 128.69, 128.66, 125.52, 125.27, 124.73, 120.18, 116.72, 110.65, 67.88, 17.54, 16.63, 15.52. HRESI-MS m/z calcd for [M-H]^-^ C_22_H_18_ClN_2_O_3_: 393.1011, found: 393.1013.

#### 4.1.3 Synthesis of 8-chloro-10-ethyl-4-methyl-1-oxo-1,2-dihydropyrazino [1,2-a]indole-3-carboxylic acid (6)

To a solution of **5** (0.25 g, 0.64 mmol) in ethanol (15 mL), 5% NaOH (4 mL) was added. The reaction mixture was kept at 40°C while stirring overnight. The residue after removal of ethanol was taken into water and acidified with 5% HCl. The formed precipitate was filtered, washed with water, and dried under vacuum to afford **6** (0.18, 96%) as a white solid. Mp 270–272°C. ^1^H NMR (400 MHz, DMSO-*d*_6_) δ 9.52 (s, 1H, pyrazine NH), 8.22 (d, *J* = 9.3 Hz, 1H, Ar-H), 7.86 (s, 1H, Ar-H), 7.30 (d, *J* = 9.2 Hz, 1H, Ar-H), 3.25 (q, *J* = 7.5 Hz, 2H, CH_2_CH_3_), 2.07 (s, 3H, CH_3_), 1.18 (t, *J* = 7.5, 3H, CH_2_CH_3_). ^13^C NMR (101 MHz, DMSO-*d*_6_) δ 164.05 (COOH), 155.67, 131.76, 129.81, 126.57, 125.99, 124.13, 121.45, 120.81, 119.45, 117.99, 117.87, 17.27, 16.47, 15.78.

#### 4.1.4 Synthesis of N-benzyl-8-chloro-10-ethyl-4-methyl-1-oxo-1,2-dihydropyrazino [1,2-a]indole-3-carboxamide (7)

To a stirred solution of **6** (0.12 g, 0.39 mmol, 1eq) in DMF (20 mL), BOP (0.22 g, 0.51 mmol, 1.3 eq), benzylamine (0.05 mL, 0.47 mmol, 1.2 eq) was added and the reaction mixture was cooled to 0°C before dropwise addition of DIPEA (0.1 mL, 0.78 mmol, 2 eq). After stirring overnight at rt, the reaction mixture was diluted with EtOAc and successively washed with H_2_O. The organic layer was washed with 5% aqueous HCl, saturated NaHCO_3_, and finally with brine. The organic phase was dried over MgSO_4,_ and the solvent was removed under reduced pressure to yield the crude product, which was purified by short column chromatography on silica gel using EtOAc as an eluent to provide **7** as g a white solid (0.13 g, 81%). Mp 290–292°C. ^1^H NMR (400 MHz, DMSO-*d*_6_) δ 10.74 (s, 1H, pyrazine NH), 9.05 (t, *J* = 5.9 Hz, 1H, NHCH_2_), 8.16 (d, *J* = 9.2 Hz, 1H, Ar-H), 7.91 (d, *J* = 2.2 Hz, 1H, Ar-H), 7.43–7.23 (m, 6H, Ar-H), 4.44 (d, *J* = 5.8 Hz, 2H, NHCH_2_), 3.25 (q, *J* = 7.4 Hz, 2H, CH_2_CH_3_), 2.73 (s, 3H, CH_3_), 1.20 (t, *J* = 7.4 Hz, 3H, CH_2_CH_3_). ^13^C NMR (101 MHz, DMSO-*d*_6_) δ 163.01 (amide C=O), 157.03 (C=O), 139.14, 131.55, 129.44, 128.73, 128.05, 127.40, 126.83, 125.03, 124.70, 121.82, 119.68, 118.51, 117.48, 117.40, 43.33, 17.30, 16.67, 16.44. HRESI-MS m/z calcd for [M-H]^-^ C_22_H_19_ClN_3_O_2_: 392.1171, found: 392.1168.

#### 4.1.5 Synthesis of ethyl 2-(5-chloro-3-ethyl-1*h*-indole-2-carboxamido)-3-phenylpropanoate (8)

This compound was prepared as described in the general procedure for synthesizing compound **3** using compound **1** and ethyl phenylalanine. Yield % 83. Oil, b. p 178–180°C. ^1^H NMR (400 MHz, Chloroform-*d*) δ 9.93 (s, 1H, indole NH), 7.55 (d, *J* = 2.1 Hz, 1H, Ar-H), 7.36–7.25 (m, 4H, Ar-H), 7.23–7.14 (m, 3H, Ar-H), 6.70 (d, *J* = 7.2 Hz, 1H, amide NH), 5.18–5.09 (m, 1H, NHCH), 4.25 (q, *J* = 7.1 Hz, 2H, OCH_2_CH_3_), 3.41–3.25 (m, 2H, PhCH_2_), 2.86–2.71 (m, 2H, CH_2_CH_3_), 1.29 (t, *J* = 7.2 Hz, 3H, OCH_2_CH_3_), 1.13 (t, *J* = 7.6 Hz, 3H, CH_2_CH_3_). ^13^C NMR (101 MHz, Chloroform-*d*) δ 171.52 (ester C=O), 161.82 (C=O), 135.59, 133.94, 129.36, 128.73, 128.68, 127.33, 127.11, 125.42, 125.07, 119.42, 119.33, 113.12, 61.90, 53.74, 37.76, 18.11, 15.21, 14.14. HRESI-MS m/z calcd for [M + H]^+^ C_22_H_24_ClN_2_O_3_: 399.1470, found: 399.1472.

#### 4.1.6 Synthesis of 3-benzyl-8-chloro-10-ethyl-2,3-dihydropyrazino [1,2-a] indole-1,4-dione (9)

This compound was prepared as described in the general procedure for synthesizing compound **5** using compound **4.** Yield % 75, mp 196–198°C. ^1^H NMR (400 MHz, DMSO-*d*_6_) δ 8.61 (d, *J* = 2.5 Hz, 1H, Ar-H), 8.31 (d, *J* = 8.7 Hz, 1H, NH), 7.77 (d, *J* = 2.2 Hz, 1H, Ar-H), 7.51 (dd, *J* = 8.8, 2.1 Hz, 1H, Ar-H), 7.02 (s, 5H, Ar-H), 4.81–4.79 (m, 1H, NHCH), 3.29 (dd, *J* = 13.6, 3.6 Hz, 1H, PhCHa), 3.10 (dd, *J* = 13.6, 5.0 Hz, 1H, PhCHb), 3.04–2.81 (m, 2H, CH_2_CH_3_), 0.98 (t, *J* = 7.4 Hz, 3H, CH_2_CH_3_). ^13^C NMR (101 MHz, DMSO-*d*_6_) δ 165.56 (CHC = O), 157.82 (C=O), 135.19, 131.68, 130.94, 130.00, 129.73, 129.55, 128.37, 127.98, 127.26, 124.00, 120.42, 117.66, 57.98, 16.68, 14.92. HRESI-MS m/z calcd for [M + H]^+^ C_20_H_18_ClN_2_O_2_: 353.1051, found: 353.1052.

#### 4.1.7 Synthesis of 3-benzyl-8-chloro-10-ethyl-4-hydroxy-3,4-dihydropyrazino [1,2-a] indol-1(2*H*)-one (10)

To a stirred solution of **10** (1 g, 2.80 mmol, 1 equiv) in ethanol (50 mL), a solution of NaBH_4_ (0.21 g, 2 equiv) in water (15 mL) was added at 0°C. The cooling bath was removed, and the reaction mixture was stirred for 1 h at rt. After the ethanol concentration *in vacuo*, the residue was extracted with EtOAc, washed with brine, dried over MgSO_4_, and concentrated under reduced pressure to afford **11** as a white solid. Yield % 93, mp 160–162°C. ^1^H NMR (400 MHz, DMSO-*d*_6_) δ 11.33 (s, 1H, NH), 7.69 (d, *J* = 8.3 Hz, 1H, CHOH), 7.61 (d, *J* = 2.1 Hz, 1H, Ar-H), 7.38 (d, *J* = 8.7 Hz, 1H, Ar-H), 7.30–7.20 (m, 4H, Ar-H), 7.15 (dd, *J* = 8.4, 2.2 Hz, 2H, Ar-H), 4.89 (s, 1H, OH), 4.24–4.14 (m, 1H, NHCH), 2.99–2.85 (m, 3H, CH_2_CH_3_, PhCH_2_a), 2.79 (dd, *J* = 13.8, 8.4 Hz, 1H, PhCH_2_b), 1.05 (t, *J* = 7.4 Hz, 3H, CH_2_CH_3_). ^13^C NMR (101 MHz, DMSO-*d*_6_) δ 161.50 (C=O), 139.54, 134.03, 129.51, 128.86, 128.61, 128.56, 126.42, 124.10, 124.01, 120.89, 119.22, 113.98, 62.88, 53.06, 37.07, 17.54, 16.02. HRESI-MS m/z calcd for [M + H]^+^ C_20_H_20_ClN_2_O_2_: 355.1208, found: 355.1210.

#### 4.1.8 3-Benzyl-8-chloro-10-ethylpyrazino[1,2-a]indol-1(2*H*)-one (11)

A mixture of **11** (0.05 g, 0.14 mmol, 1equiv) and PTSA (0.05 g, 2 equiv) in toluene (10 mL) was refluxed for 4 h. After removing the solvent *in vacuo*, the residue was extracted with EtOAc, washed with saturated solution of NaHCO_3_, brine, dried over MgSO_4_, and concentrated under reduced pressure to yield a crude product which was purified by flash chromatography using EtOAc/hexanes (1:4) as eluent to provide **12** as a brown solid. Yield % 60, mp 261–263°C. ^1^H NMR (400 MHz, DMSO-*d*_6_) δ 10.67 (s, 1H, pyrazine NH), 7.94 (d, *J* = 8.9 Hz, 1H, Ar-H), 7.85 (d, *J* = 2.0 Hz, 1H, Ar-H), 7.71 (d, *J* = 1.7 Hz, 1H, Ar-H), 7.41–7.27 (m, 5H, Ar-H), 7.26–7.19 (m, 1H, Ar-H), 3.69 (s, 2H, PhCH_2_), 3.18 (q, *J* = 7.4 Hz, 2H, CH_2_CH_3_), 1.18 (t, *J* = 7.4 Hz, 4H, CH_2_CH_3_). ^13^C NMR (101 MHz, DMSO-*d*_6_) δ 158.57 (C=O), 138.74, 129.50, 129.03, 128.83, 127.62, 127.01, 126.46, 124.84, 124.32, 123.31, 119.77, 119.66, 113.66, 103.81, 35.82, 17.36, 16.67. HRESI-MS m/z calcd for [M + H]^+^ C_20_H_18_ClN_2_O: 337.1102, found: 337.1103.

#### 4.1.9 3-Benzyl-8-chloro-10-ethyl-2-methylpyrazino[1,2-a]indol-1(2*H*)-one (13)

A solution of 12 (1 equiv) in DMF (0.2 M) was added dropwise at 0°C to a suspension of NaH (1.5 equiv, 60% dispersion in mineral oil) in DMF (0.2 M). After stirring for 0.5 h, the resulting mixture was treated dropwise with a MeI (1.2 equiv). The cooling bath was removed, and the mixture was stirred overnight at rt. The reaction mixture was diluted with EtOAc and successively washed twice with water. The EtOAc layer was washed with brine, dried over MgSO_4,_ and concentrated under reduced pressure to yield 13 as a white solid after column chromatography using EtOAc/hexane (2:3) as an eluent. Yield % 88, mp 161–163°C. ^1^H NMR (400 MHz, Chloroform-*d*) δ 7.70 (s, 1H, Ar-H), 7.38 (d, *J* = 9.0 Hz, 1H, Ar-H), 7.33–7.15 (m, 6H, Ar-H), 6.90 (s, 1H, Ar-H), 3.86 (s, 2H, PhCH_2_), 3.26–3.19 (m, 5H, CH_3_, CH_2_CH_3_), 1.25 (t, *J* = 7.6 Hz, 3H, CH_2_CH_3_). ^13^C NMR (101 MHz, Chloroform-*d*) δ 158.81 (C=O), 136.66, 129.00, 128.81, 128.22, 128.16, 127.25, 124.51, 124.29, 122.62, 120.82, 119.93, 111.38, 105.40, 37.11, 29.26, 17.68, 16.06. HRESI-MS m/z calcd for [M + H]^+^ C_21_H_20_ClN_2_O: 351.1259, found: 351.1257.

#### 4.1.10 Methyl 2-(5-chloro-3-ethyl-1*h*-indole-2-carboxamido)-2-phenylacetate (13)

This compound was prepared as described in the general procedure for synthesizing compound **3** using compound **1** and methyl-2-amino-2-phenylacetate. Yield % 94, mp 210–212°C. ^1^H NMR (400 MHz, Chloroform-*d*) δ 9.77 (s, 1H, indole NH), 7.59 (d, *J* = 2.0 Hz, 1H, Ar-H), 7.49–7.44 (m, 2H, Ar-H), 7.44–7.34 (m, 4H, Ar-H), 7.13 (dd, *J* = 8.7, 2.0 Hz, 1H, Ar-H), 7.01 (d, *J* = 8.7 Hz, 1H, Ar-H, amide NH), 5.79 (d, *J* = 6.4 Hz,1H, PhCH), 3.78 (s, 3H, OCH_3_), 3.06 (q, *J* = 7.6 Hz, 2H, CH_2_CH_3_), 1.40 (t, *J* = 7.6 Hz, 3H, CH_2_CH_3_). ^13^C NMR (101 MHz, Chloroform-*d*) δ 171.37 (**ester C=O**), 161.42 (C=O), 136.46, 133.89, 129.09, 128.69, 127.13, 126.96, 125.48, 125.16, 119.55, 119.28, 113.13, 56.86, 53.16, 18.50, 15.43. RESI-MS m/z calcd for [M + H]^+^ C_20_H_20_ClN_2_O_3_: 371.1157, found: 371.1156.

#### 4.1.11 8-Chloro-10-ethyl-3-phenyl-2,3-dihydropyrazino [1,2-a] indole-1,4-dione (14)

This compound was prepared as described in the general procedure for synthesizing compound **5** using compound **13.** Yield % 85, mp 202–204°C. ^1^H NMR (400 MHz, Chloroform-*d*) δ 8.33 (d, *J* = 8.8 Hz, 1H, Ar-H), 7.68 (d, *J* = 2.0 Hz, 1H, Ar-H), 7.49–7.35 (m, 6H, Ar-H), 6.35 (s, 1H, NH), 5.46 (d, *J* = 2.2 Hz, 1H, PhCH), 3.24 (q, *J* = 7.4 Hz, 2H, CH_2_CH_3_), 1.33 (t, *J* = 7.5 Hz, 3H, CH_2_CH_3_). ^13^C NMR (101 MHz, Chloroform-*d*) δ 162.97, 158.14, 136.68, 133.61, 132.64, 131.07, 130.90, 129.33, 129.26, 128.65, 127.07, 122.30, 120.27, 117.85, 61.72, 17.51, 14.37. HRESI-MS m/z calcd for [M-H]^-^ C_19_H_14_ClN_2_O_2_: 337.0749, found: 337.0753.

#### 4.1.12 8-Chloro-10-ethyl-4-hydroxy-3-phenyl-3,4-dihydropyrazino [1,2-a] indol-1(2*H*)-one (15)

This compound was prepared as described in the general procedure for synthesizing compound **10** using compound **14**. Yield % 89, mp 213–215°C. ^1^H NMR (400 MHz, DMSO-*d*_6_) δ 8.54 (d, *J* = 4.7 Hz, 1H, NH), 7.70 (d, *J* = 2.1 Hz, 1H, Ar-H), 7.51 (d, *J* = 8.9 Hz, 1H, Ar-H), 7.29–7.12 (m, 6H, Ar-H), 6.98 (d, *J* = 5.7 Hz, 1H, CHOH), 6.02 (d, *J* = 5.8 Hz, 1H, OH), 4.86–4.79 (m, 1H, PhCH), 3.11 (q, *J* = 7.5 Hz, 2H, CH_2_CH_3_), 1.19 (t, *J* = 7.4 Hz, 3H, CH_2_CH_3_). ^13^C NMR (101 MHz, DMSO-*d*_6_) δ 160.55 (C=O), 140.14, 133.89, 129.00, 128.39, 128.04, 126.53, 125.22, 124.83, 124.43, 123.68, 119.70, 112.95, 76.41, 61.00, 17.35, 15.94. HRESI-MS m/z calcd for [M-H]^-^ C_19_H_16_ClN_2_O_2_: 339.0906, found: 339.0904.

#### 4.1.13 8-Chloro-10-ethyl-3-phenylpyrazino [1,2-a]indol-1(2*H*)-one (16)

This compound was prepared as described in the general procedure for synthesizing compound **11** using compound **15**. Yield % 88, mp 263–265°C. ^1^H NMR (400 MHz, DMSO-*d*_6_) δ 10.89 (s, 1H, pyrazine NH), 8.20 (d, *J* = 8.9 Hz, 1H, Ar-H), 8.15 (d, *J* = 1.6 Hz, 1H, Ar-H), 7.89 (d, *J* = 2.1 Hz, 1H, Ar-H), 7.80–7.72 (m, 2H, Ar-H), 7.50–7.33 (m, 4H, Ar-H), 3.23 (q, *J* = 7.4 Hz, 2H, CH_2_CH_3_), 1.23 (t, *J* = 7.4 Hz, 3H, CH_2_CH_3_). ^13^C NMR (101 MHz, DMSO-*d*_6_) δ 158.65 (C=O), 132.50, 130.11, 129.10, 128.75, 128.01, 126.91, 126.23, 124.96, 124.51, 123.17, 120.30, 119.80, 114.29, 104.24, 17.42, 16.62. HRESI-MS m/z calcd for [M + H]^+^ C_19_H_16_ClN_2_O: 323.0946, found: 323.0947.

### 4.2 Biology

#### 4.2.1 Assay of cell viability effect

The normal human mammary gland epithelial (MCF-10A) cell line was used to test the viability of the tested compounds. After 4 days of incubation on MCF-10A cells within 50 µM of each investigated compound, cell viability was determined using the MTT assay (Gomaa et al., 2020; Marzouk et al., 2020). Refer to Supplementary Appendix SA1 (supp. File) for more details.

#### 4.2.2 Antiproliferative assay

The MTT assay was used to investigate the antiproliferative activity of **3–16** *versus* four human cancer cell lines using erlotinib as a control (Youssif et al., 2022; Mahmoud et al., 2022). See Supplementary Appendix SA1 for more details.

#### 4.2.3 Aromatase inhibitory assay

Compounds **5**, **7**, **8**, **12**, **13**, and **16** were tested for their capacity to inhibit aromatase, a potential target for their antiproliferative activity, using ketoconazole and letrozole as reference drugs (Maghraby et al., 2023). See Supplementary Appendix SA1 for more information.

#### 4.2.4 Assay of nitric oxide synthase inhibition

The L-citrulline assay method with fluorometric detection was used to test compounds **5**, **7**, **12**, and **16** (most potent anti-aromatase derivatives) for their ability to inhibit iNOS (Carrión et al., 2023). Refer to Supplementary Appendix SA1 for more details.

### 4.3 *In silico* studies

#### 4.3.1 Docking study

In our molecular docking study, we employed BIOVIA Discovery Studio 2021 software (version 21.1.0.20.298) (Pawar and Rohane, 2021). We utilized the Protein Preparation Wizard to prepare the selected proteins for docking analysis. Following protein preparation, we meticulously mapped the ligands onto a three-dimensional model and conducted energy minimization using LigPrep. We generated a receptor grid tailored to the selected binding site to optimize potential binding interactions using the Receptor Grid Generation Tool. Subsequently, the Glide tool was employed to comprehensively assess both docking scores and the diverse binding modes exhibited by the ligands. This rigorous approach facilitated a thorough exploration of the binding affinities and interaction patterns between the ligands and the selected proteins, enhancing our understanding of their potential as inhibitors in breast cancer therapy.

#### 4.3.2 *In silico* ADMET analysis

In our study, ADMET (Absorption, Distribution, Metabolism, Excretion, and Toxicity) studies were conducted using BIOVIA I Discovery Studio 2021 (Mohammed Ali et al., 2023). The chemical structures of all compounds were imported, and ADMET descriptors were predicted using integrated models. These models encompassed assessments based on Lipinski’s Rule of Five and evaluations of absorption, distribution, metabolism, excretion, and toxicity. The resulting data were meticulously analysed to determine the drug-likeness and safety profiles of the compounds under investigation.

## Data Availability

The original contributions presented in the study are included in the article/Supplementary Material, further inquiries can be directed to the corresponding authors.
