# Supplementary material for: Design and synthesis new indole-based aromatase/iNOS inhibitors with apoptotic antiproliferative activity
Source: Front Chem. 2024 Sep 6;12:1432920. doi: 10.3389/fchem.2024.1432920 (PMC11414412; doi:10.3389/fchem.2024.1432920)
Supplement: Supplementary file 1 [file DataSheet1.PDF]

Design and synthesis new indole-based aromatase/iNOS inhibitors with apoptotic antiproliferative activity

Lamya H. Al-Wahaibi[1], Hesham A. Abou-Zied[2], Mostafa H. Abdelrahman[3], Laurent Trembleau[4]*, Bahaa G. M. Youssif[5]*, S. Bräse[6]*

[1]Department of Chemistry, College of Sciences, Princess Nourah bint Abdulrahman University, Riyadh 11671, Saudi Arabia; [2]Medicinal Chemistry Department, Faculty of Pharmacy, Deraya University, Minia, Egypt; [3]Pharmaceutical Organic Chemistry Department, Faculty of Pharmacy, Al-Azhar University, Assiut Branch, Assiut 71524, Egypt; [4]School of Natural and Computing Sciences, University of Aberdeen, Meston Building, Aberdeen, AB24 3UE, United Kingdom. [5]Pharmaceutical Organic Chemistry Department, Faculty of Pharmacy, Assiut University, Assiut 71526, Egypt; [6]Institute of Biological and Chemical Systems, IBCS-FMS, Karlsruhe Institute of Technology, 76131 Karlsruhe, Germany.

**To whom correspondence should be addressed:*

Bahaa G. M. Youssif, Ph.D. Pharmaceutical Organic Chemistry Department, Faculty of Pharmacy, Assiut University, Assiut 71526, Egypt.

Tel.: (002)-01098294419

E-mail address: bgyoussif2@gmail.com

S. Bräse

Institute of Biological and Chemical Systems, IBCS-FMS, Karlsruhe Institute of Technology, 76131 Karlsruhe, Germany. E-mail: braese@kit.edu

Laurent Trembleau

Ph.D. University of Aberdeen, Chemistry Department, The SyMBioSIS Group, Meston Building, Meston Walk, Aberdeen, AB24 3UE, United Kingdom.

Tel.: +44-(0)12242922

E-mail address: l.trembleau@abdn.ac.uk

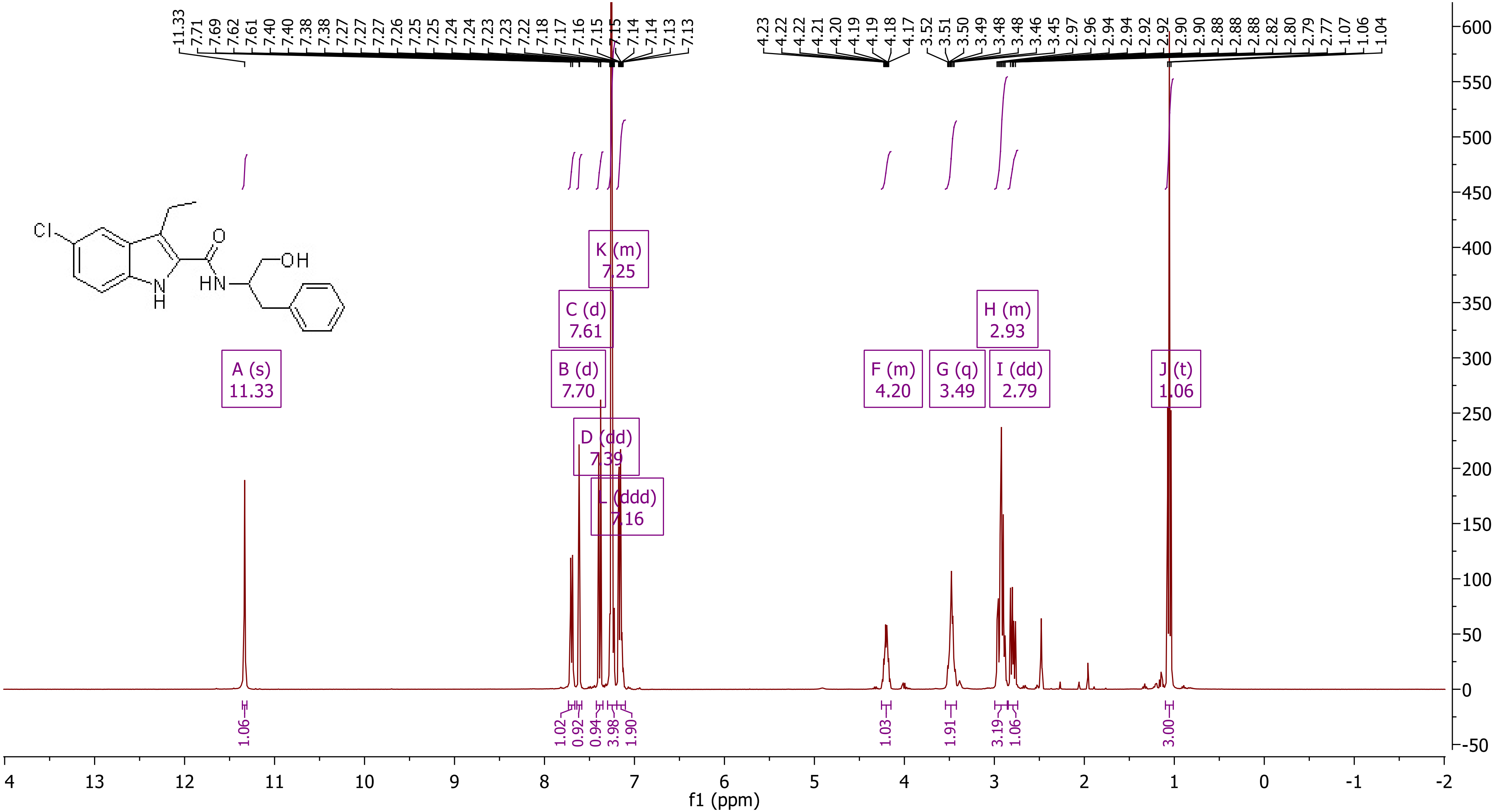

^{1}H NMR (400 MHz, DMSO-d_6) δ 11.33 (s, 1H), 7.70 (d, J = 8.3 Hz, 1H), 7.61 (d, J = 2.0 Hz, 1H), 7.39 (d, J = 8.7 Hz, 1H), 7.30 – 7.20 (m, 4H), 7.18 - 7.12 (m, 2H), 4.25 – 4.15 (m, 1H), 3.52 - 3.45 (m, 2H), 3.00 – 2.86 (m, 3H), 2.79 (dd, J = 13.8, 8.4 Hz, 1H), 1.06 (t, J = 7.4 Hz, 3H).

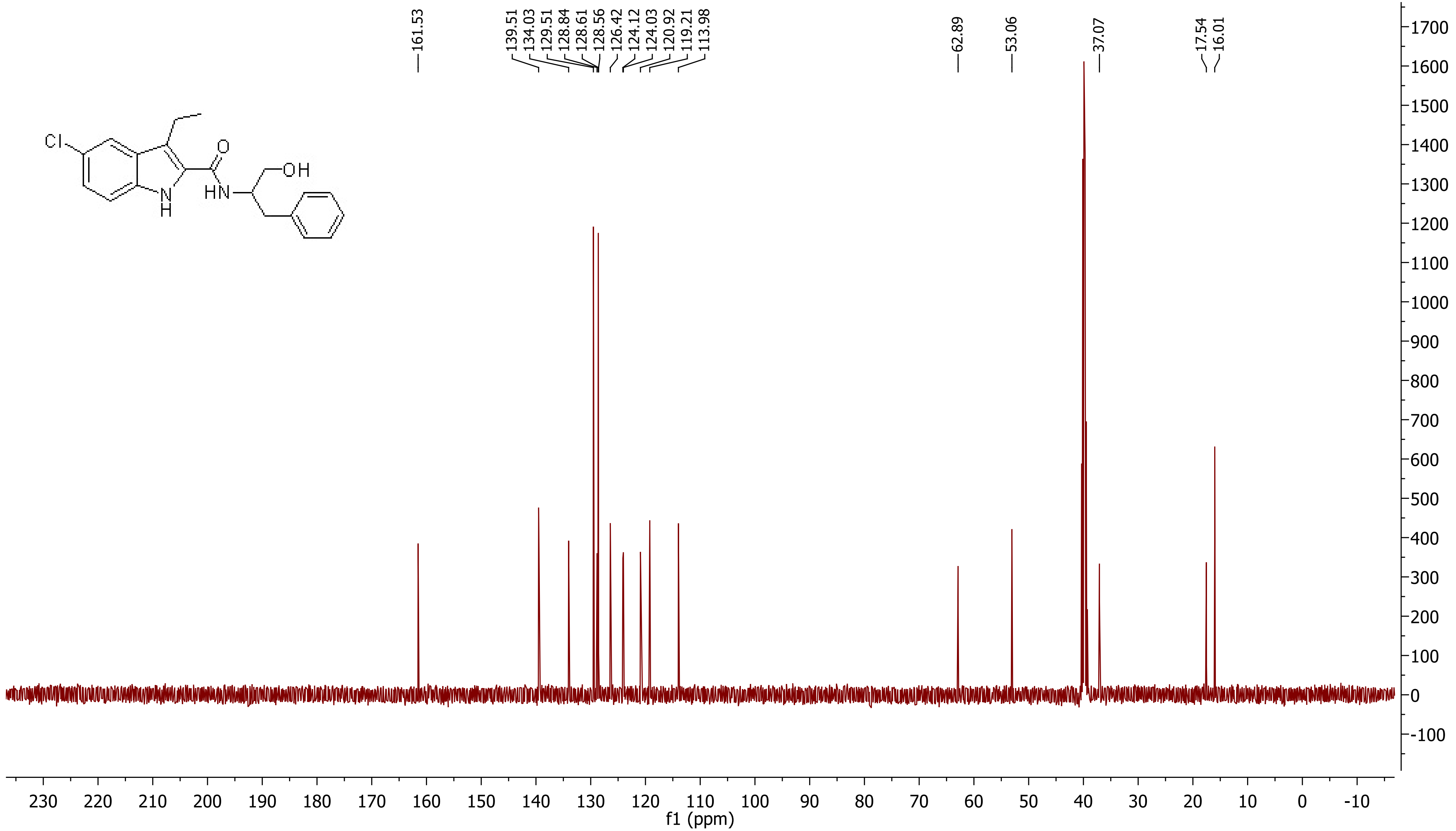

^{13}C NMR (101 MHz, dmso) δ 161.53, 139.51, 134.03, 129.51, 128.84, 128.61, 128.56, 126.42, 124.12, 124.03, 120.92, 119.21, 113.98, 62.89, 53.06, 37.07, 17.54, 16.01.

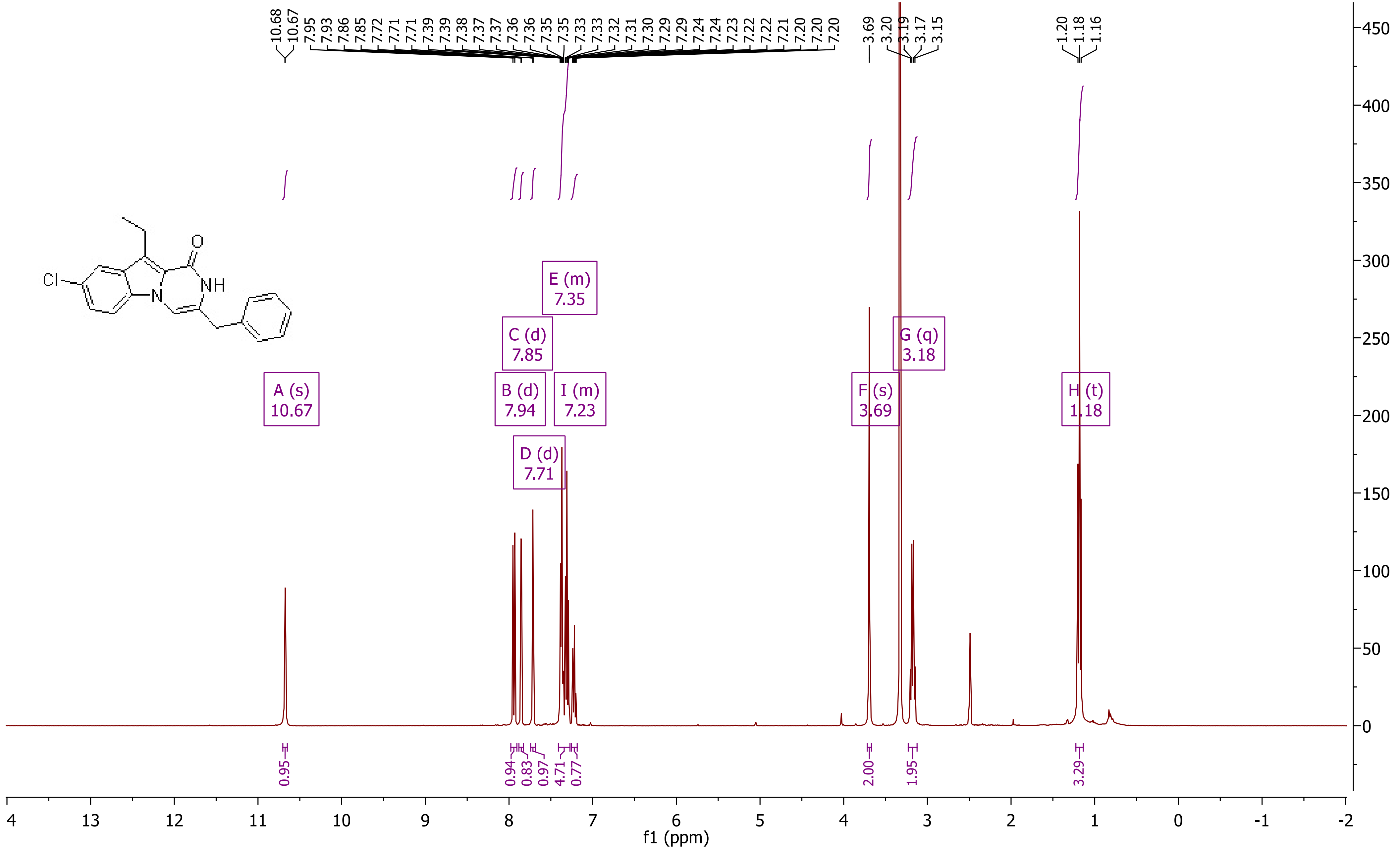

^{1}H NMR (400 MHz, DMSO-d_6) δ 10.67 (s, 1H), 7.94 (d, J = 8.9 Hz, 1H), 7.85 (d, J = 2.0 Hz, 1H), 7.71 (d, J = 1.7 Hz, 1H), 7.41 – 7.27 (m, 5H), 7.26 – 7.19 (m, 1H), 3.69 (s, 2H), 3.18 (q, J = 7.4 Hz, 2H), 1.18 (t, J = 7.4 Hz, 3H).

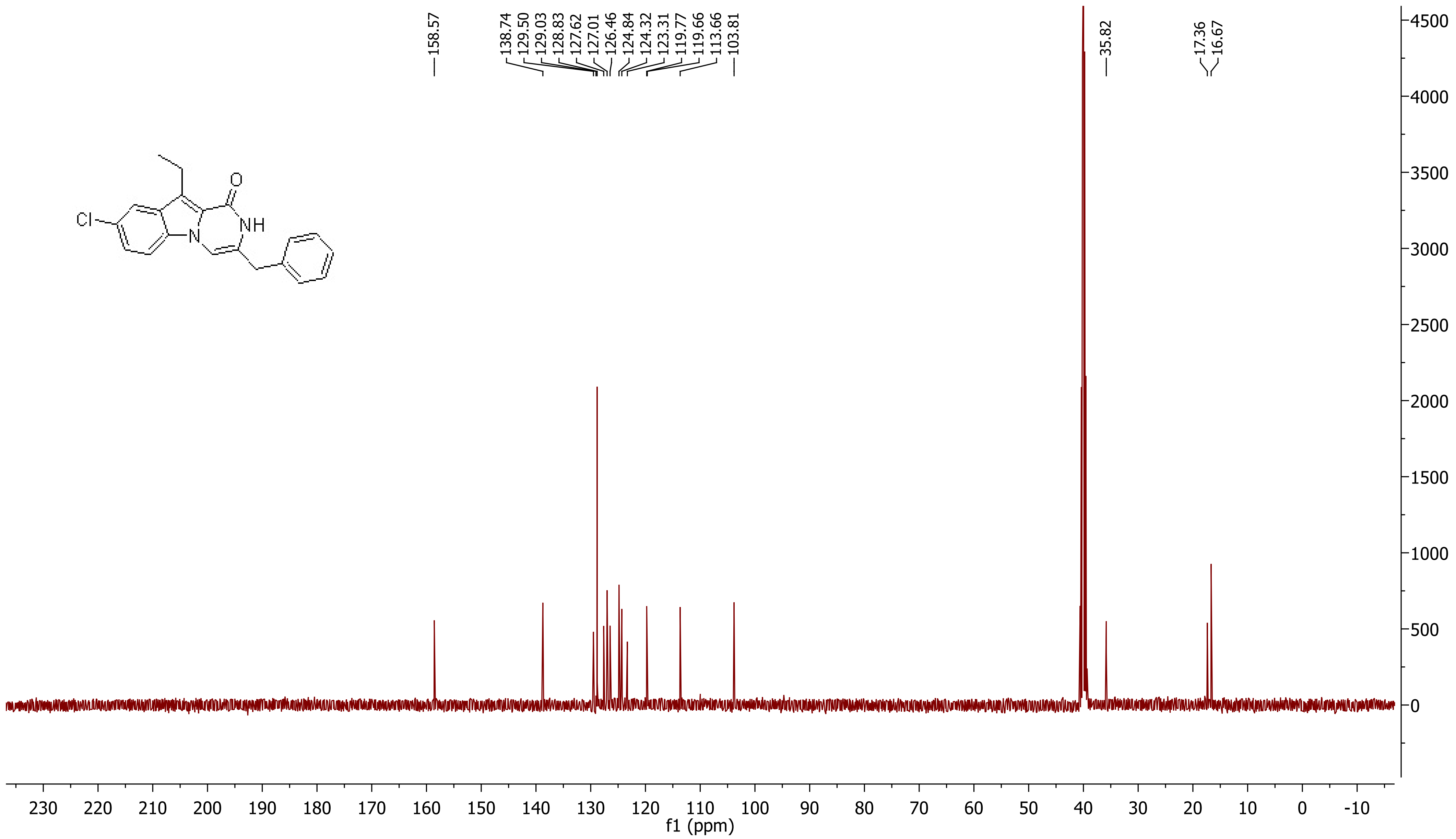

^{13}C NMR (101 MHz, dmso) δ 158.57, 138.74, 129.50, 129.03, 128.83, 127.62, 127.01, 126.46, 124.84, 124.32, 123.31, 119.77, 119.66, 113.66, 103.81, 35.82, 17.36, 16.67.

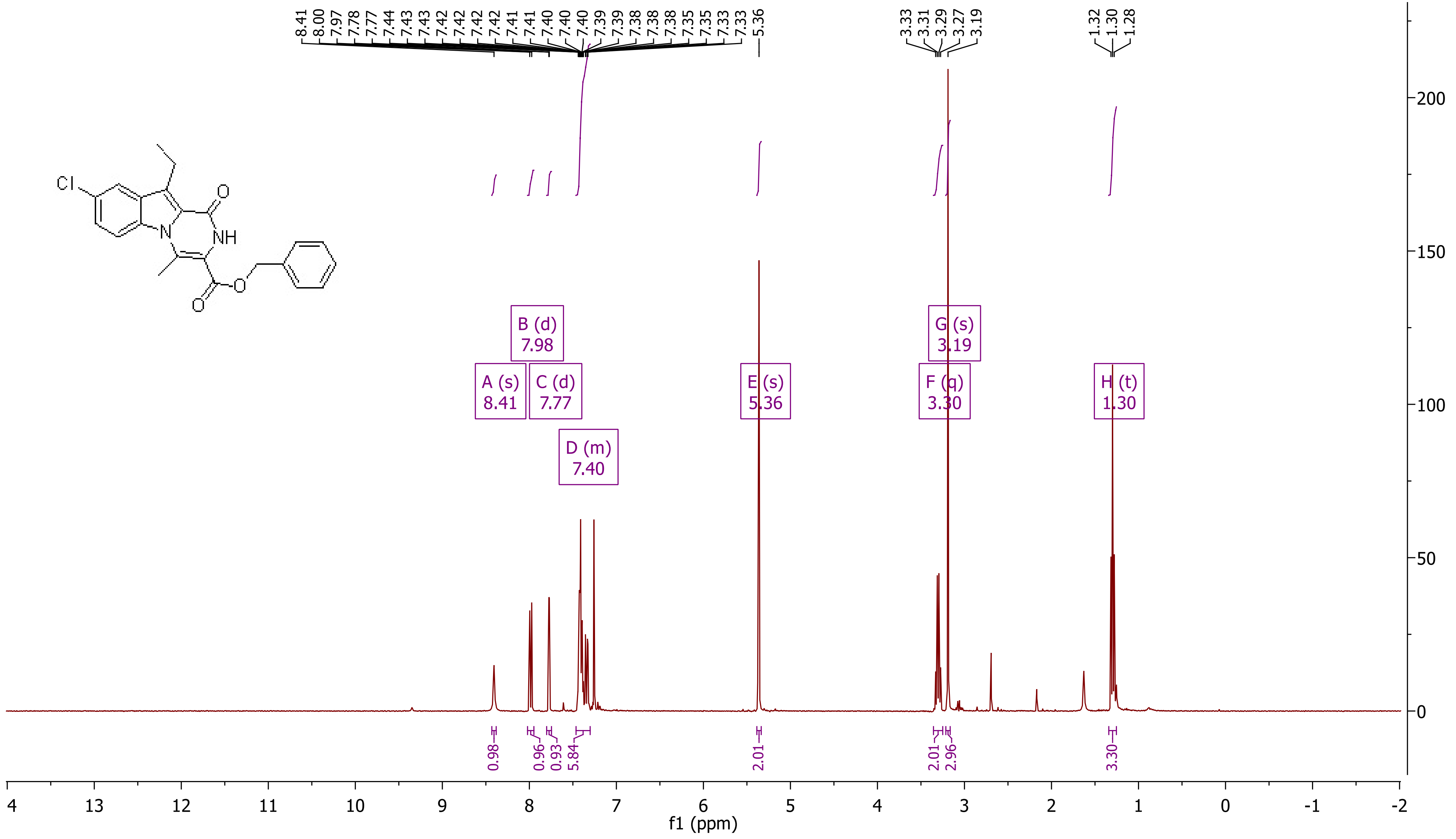

^{1}H NMR (400 MHz, Chloroform-*d*) δ 8.41 (s, 1H), 7.98 (d, *J* = 9.2 Hz, 1H), 7.77 (d, *J* = 2.1 Hz, 1H), 7.46 – 7.30 (m, 6H), 5.36 (s, 2H), 3.30 (q, *J* = 7.5 Hz, 2H), 3.19 (s, 3H), 1.30 (t, *J* = 7.5 Hz, 3H).

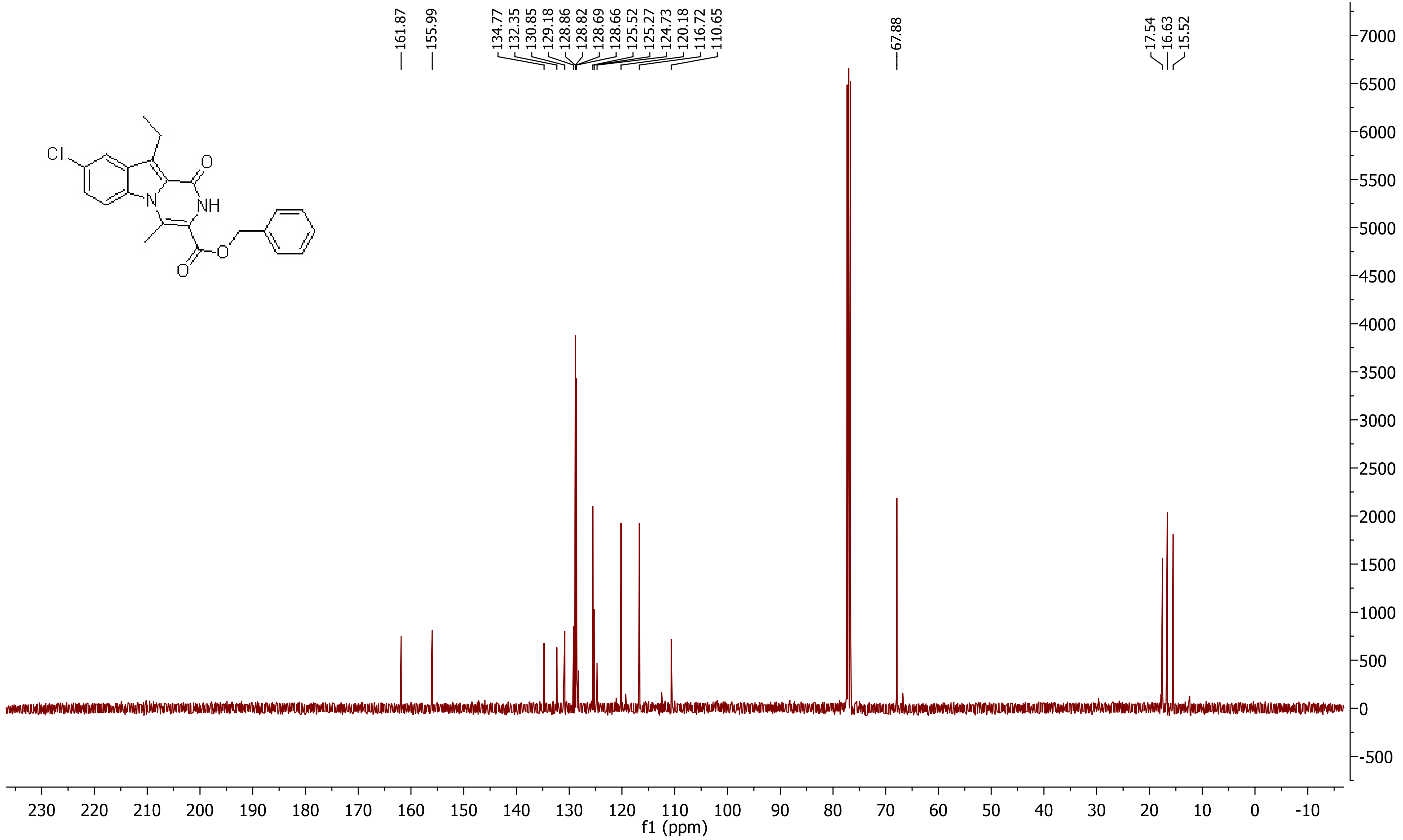

^{13}C NMR (101 MHz, cdcl$_3$) δ 161.87, 155.99, 134.77, 132.35, 130.85, 129.18, 128.86, 128.82, 128.69, 128.66, 125.52, 125.27, 124.73, 120.18, 116.72, 110.65, 67.88, 17.54, 16.63, 15.52.

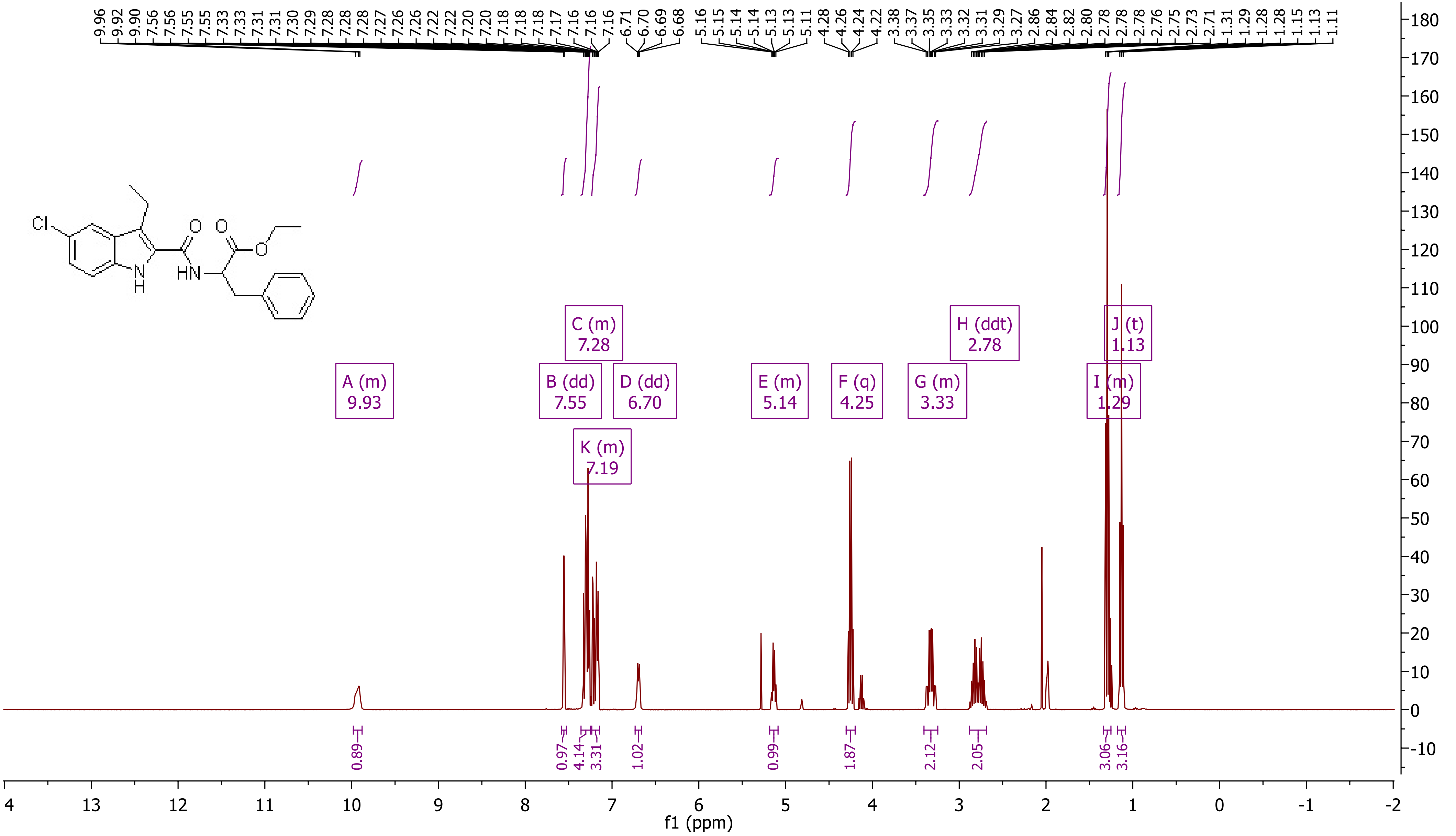

^{1}H NMR (400 MHz, Chloroform-*d*) δ 9.93 (s, 1H), 7.55 (d, *J* = 2.1 Hz, 1H), 7.36 – 7.25 (m, 4H), 7.23 – 7.14 (m, 3H), 6.70 (d, *J* = 7.2 Hz, 1H), 5.18 – 5.09 (m, 1H), 4.25 (q, *J* = 7.1 Hz, 2H), 3.41 – 3.25 (m, 2H), 2.86 - 2.71 (m, 2H), 1.29 (t, *J* = 7.2 Hz, 3H), 1.13 (t, *J* = 7.6 Hz, 3H).

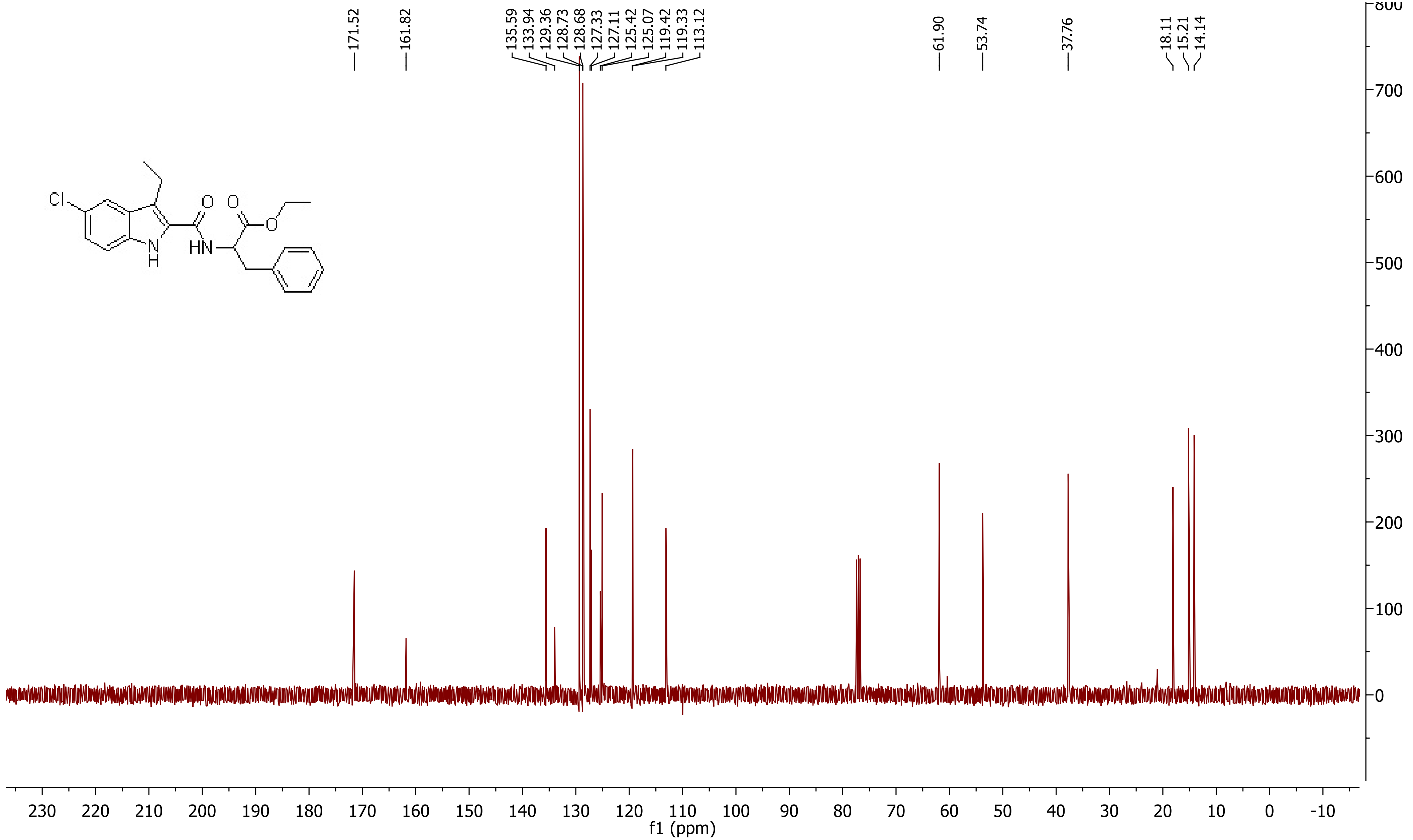

^{13}C NMR (101 MHz, cdcl$_3$) δ 171.52, 161.82, 135.59, 133.94, 129.36, 128.73, 128.68, 127.33, 127.11, 125.42, 125.07, 119.42, 119.33, 113.12, 61.90, 53.74, 37.76, 18.11, 15.21, 14.14.

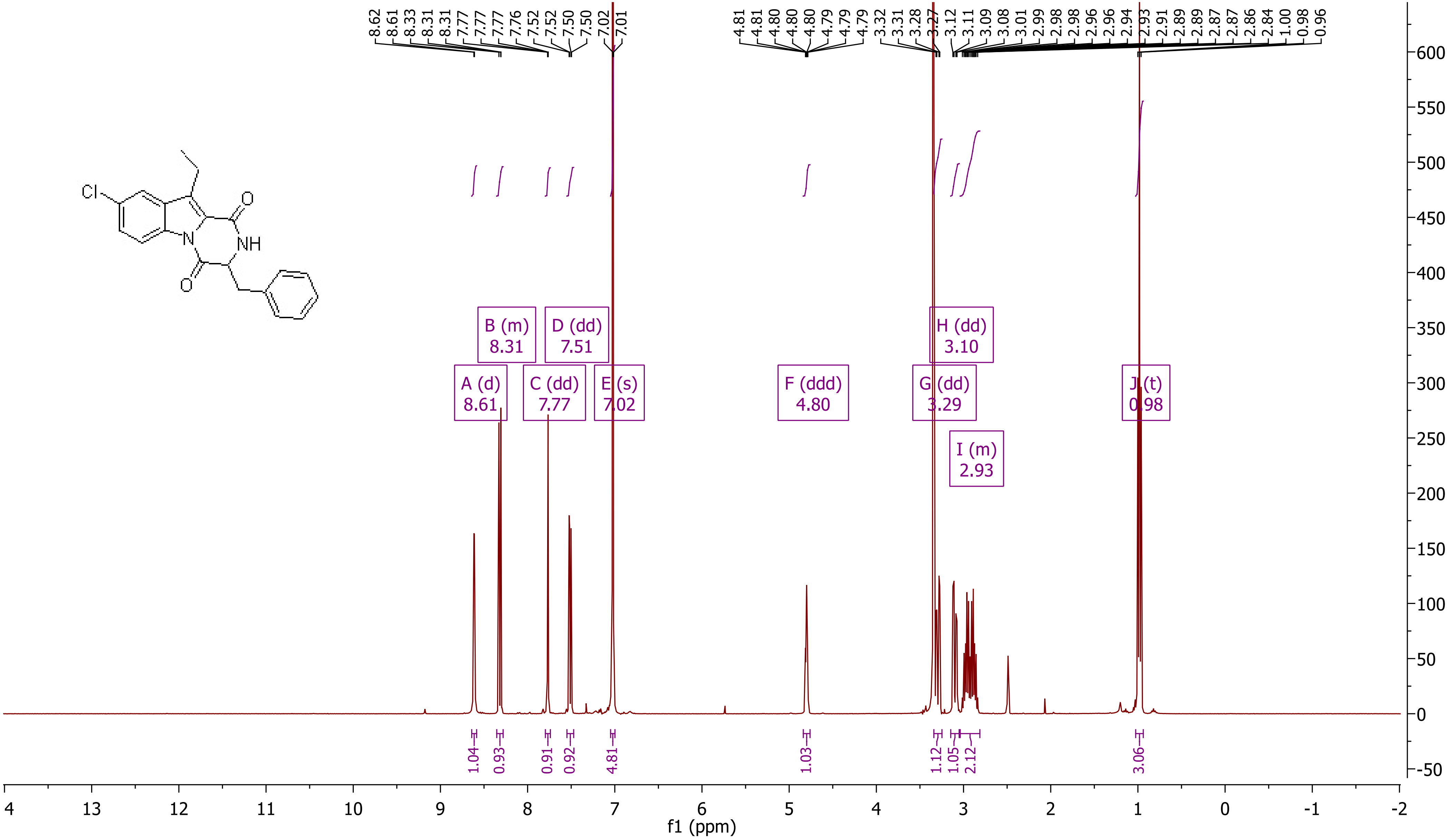

^{1}H NMR (400 MHz, DMSO-d_6) δ 8.61 (d, J = 2.5 Hz, 1H), 8.31 (d, J = 8.7 Hz, 1H), 7.77 (d, J = 2.2 Hz, 1H), 7.51 (dd, J = 8.8, 2.1 Hz, 1H), 7.02 (s, 5H), 4.81 - 4.79 (m, 1H), 3.29 (dd, J = 13.6, 3.6 Hz, 1H), 3.10 (dd, J = 13.6, 5.0 Hz, 1H), 3.04 – 2.81 (m, 2H), 0.98 (t, J = 7.4 Hz, 3H).

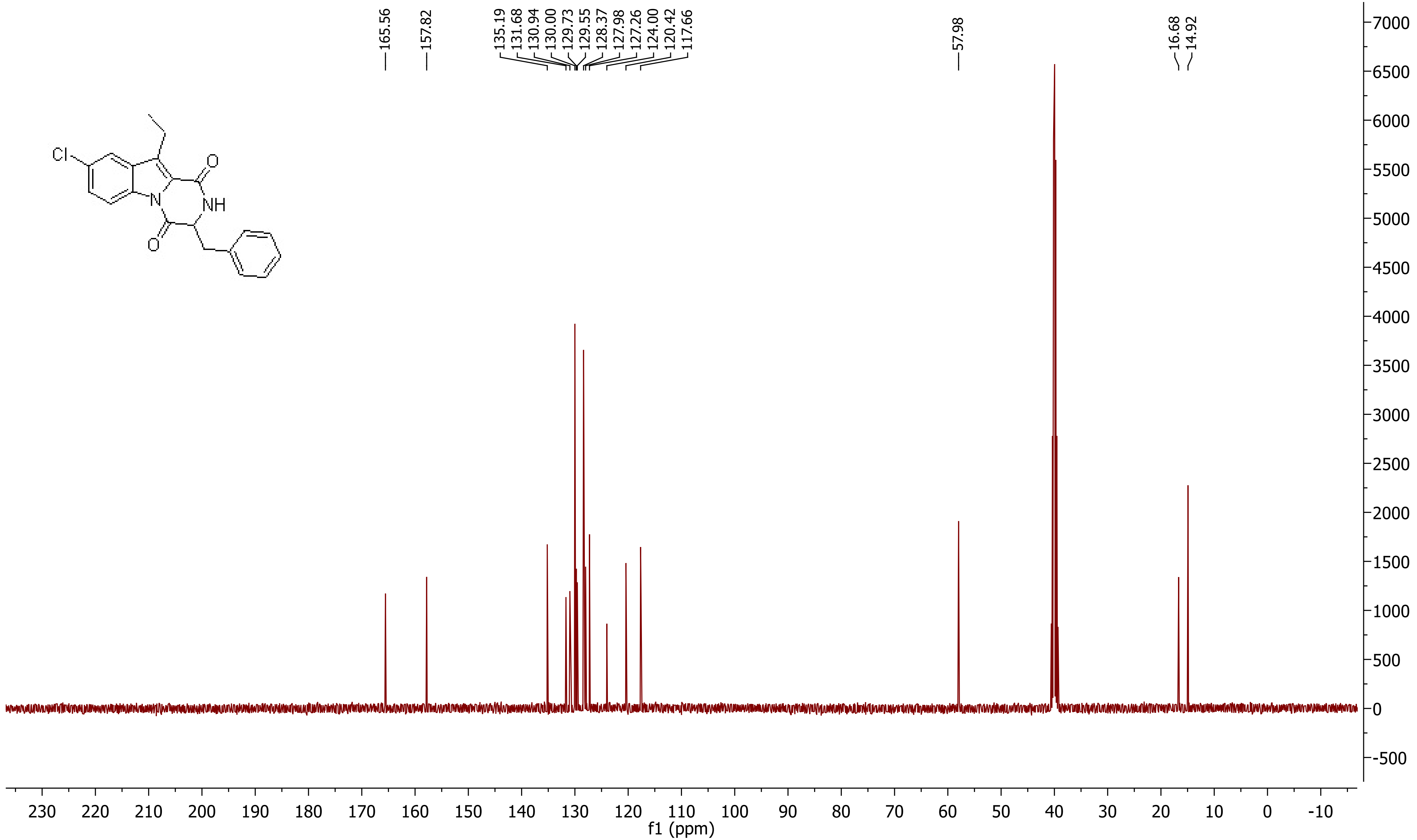

^{13}C NMR (101 MHz, dmso) δ 165.56, 157.82, 135.19, 131.68, 130.94, 130.00, 129.73, 129.55, 128.37, 127.98, 127.26, 124.00, 120.42, 117.66, 57.98, 16.68, 14.92.

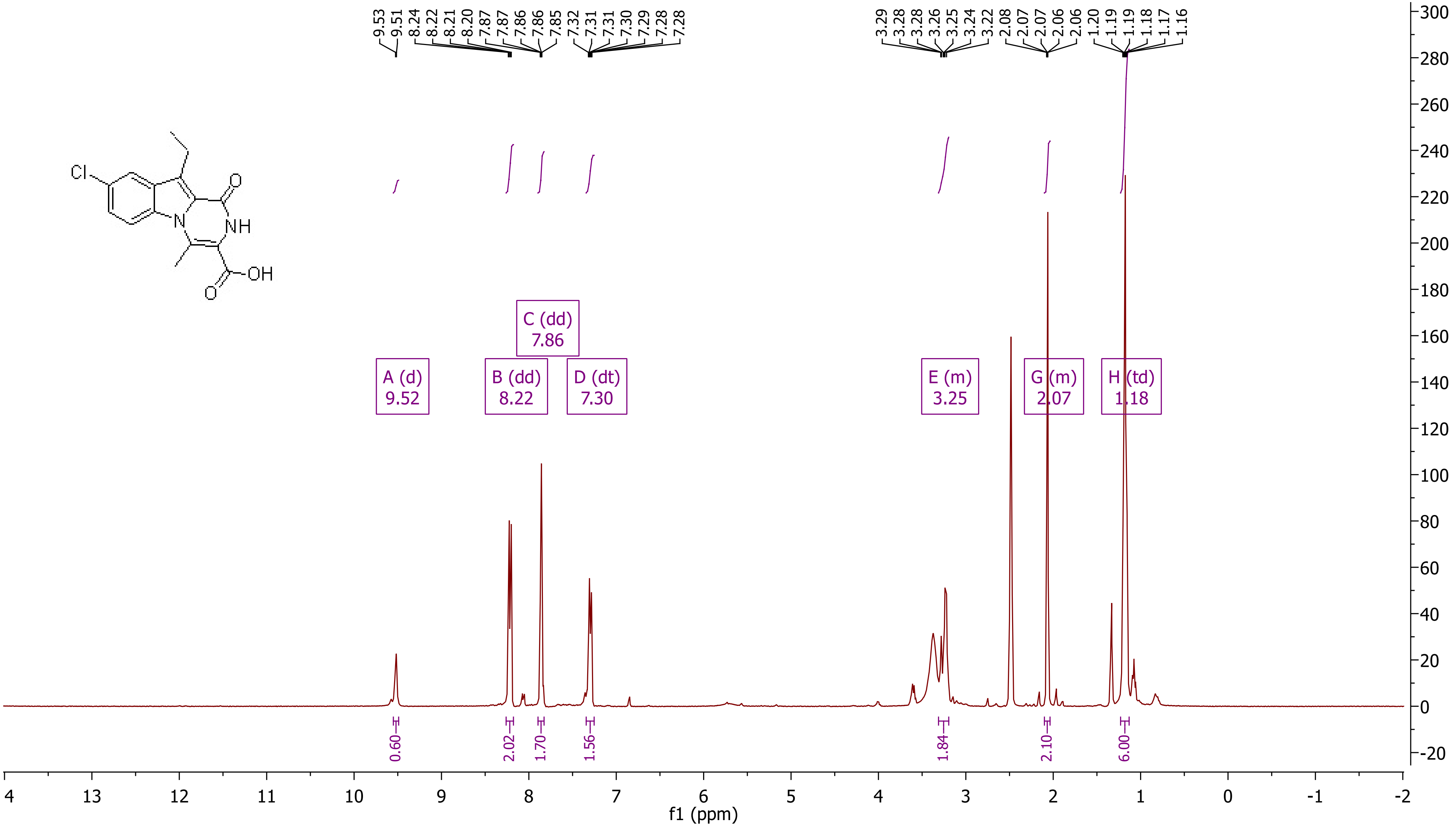

^{1}H NMR (400 MHz, DMSO-d_6) δ 9.52 (s, 1H), 8.22 (d, J = 9.3 Hz, 1H), 7.86 (s, 1H), 7.30 (d, J = 9.2 Hz, 1H), 3.25 (q, J = 7.5 Hz, 2H), 2.07 (s, 3H), 1.18 (t, J = 7.5, 3H).

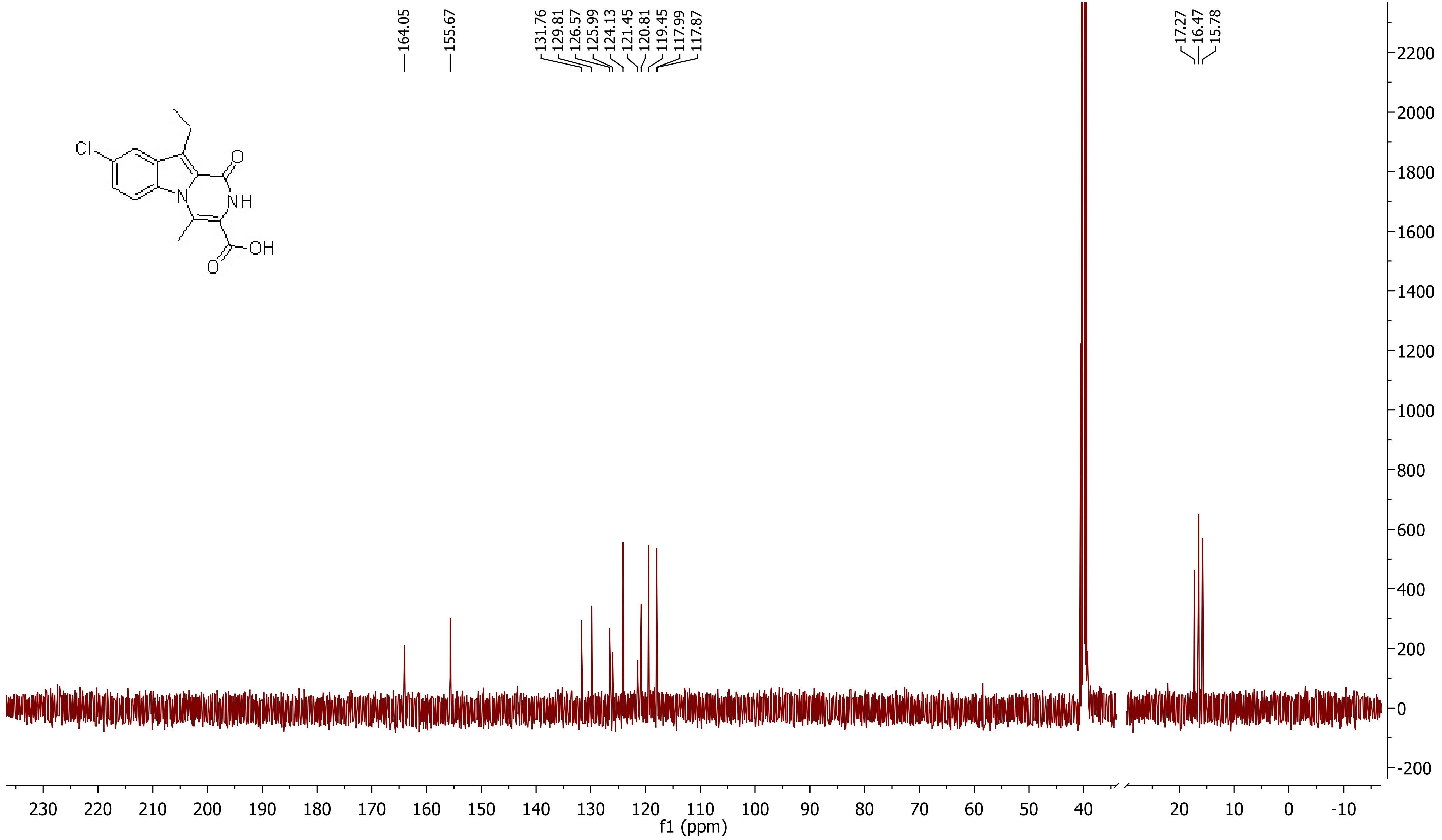

^{13}C NMR (101 MHz, dmso) δ 164.05, 155.67, 131.76, 129.81, 126.57, 125.99, 124.13, 121.45, 120.81, 119.45, 117.99, 117.87, 17.27, 16.47, 15.78.

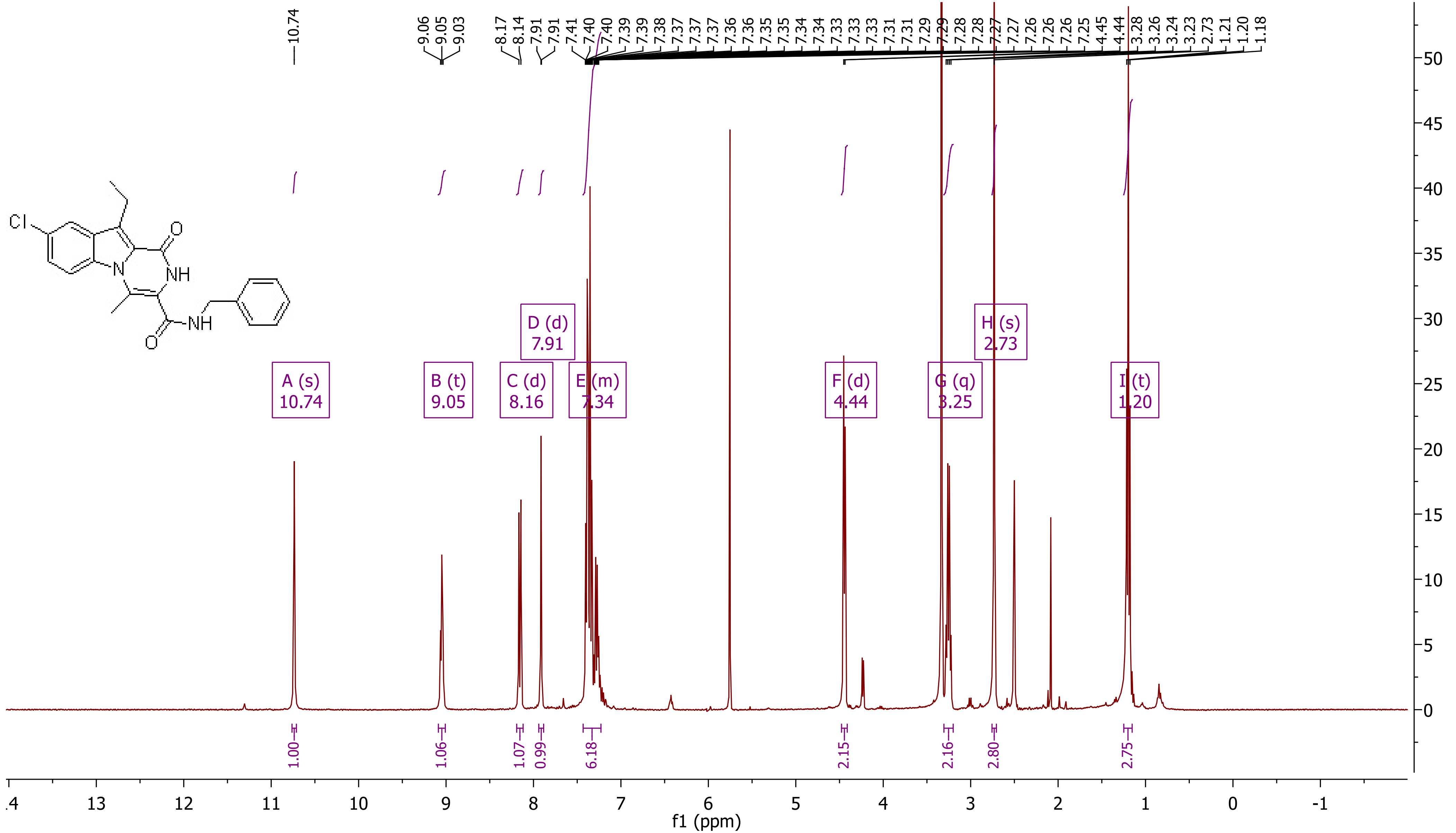

^{1}H NMR (400 MHz, DMSO-d_6) δ 10.74 (s, 1H), 9.05 (t, J = 5.9 Hz, 1H), 8.16 (d, J = 9.2 Hz, 1H), 7.91 (d, J = 2.2 Hz, 1H), 7.43 – 7.23 (m, 6H), 4.44 (d, J = 5.8 Hz, 2H), 3.25 (q, J = 7.4 Hz, 2H), 2.73 (s, 3H), 1.20 (t, J = 7.4 Hz, 3H).

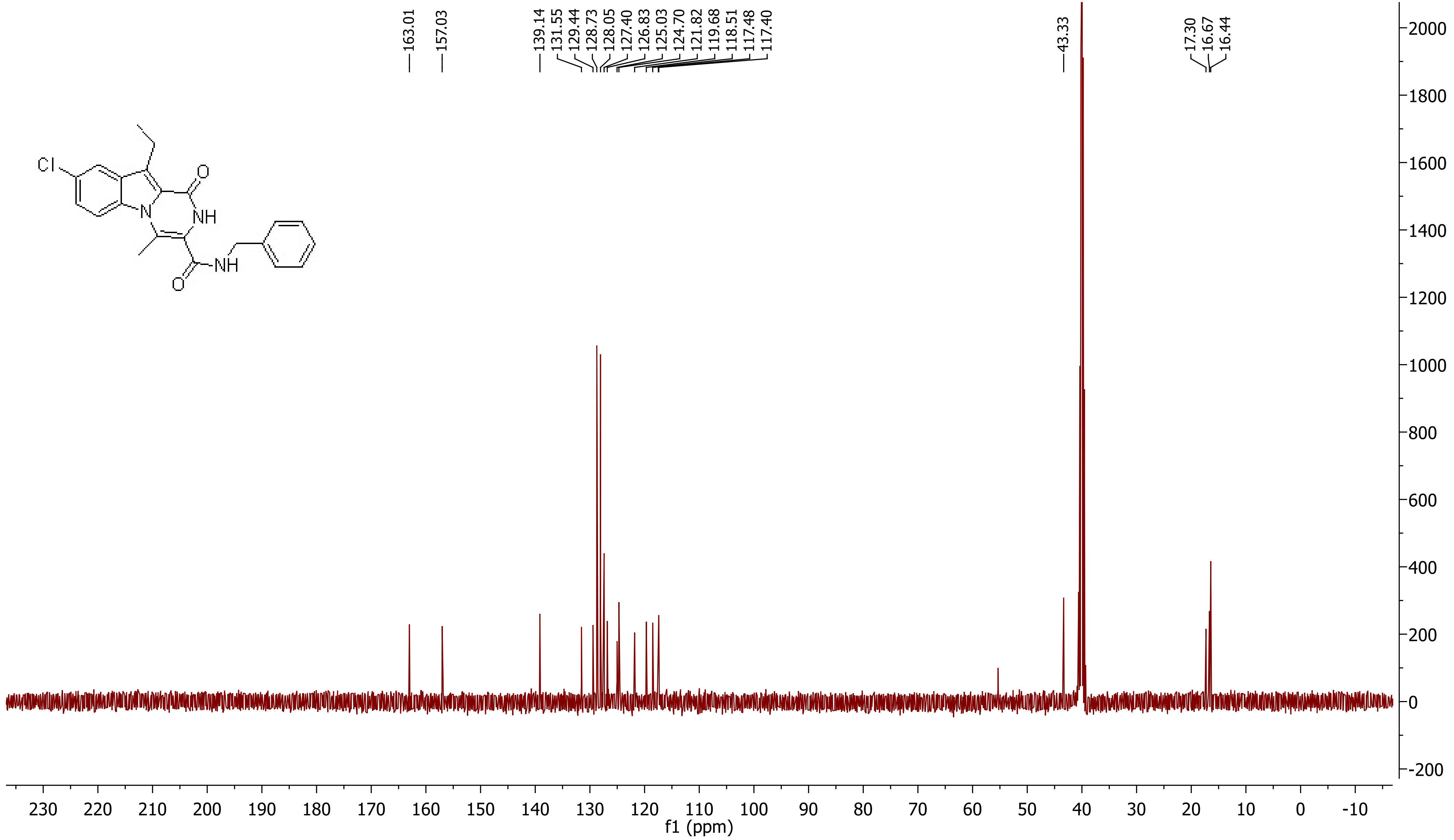

^{13}C NMR (101 MHz, dmso) δ 163.01, 157.03, 139.14, 131.55, 129.44, 128.73, 128.05, 127.40, 126.83, 125.03, 124.70, 121.82, 119.68, 118.51, 117.48, 117.40, 43.33, 17.30, 16.67, 16.44.

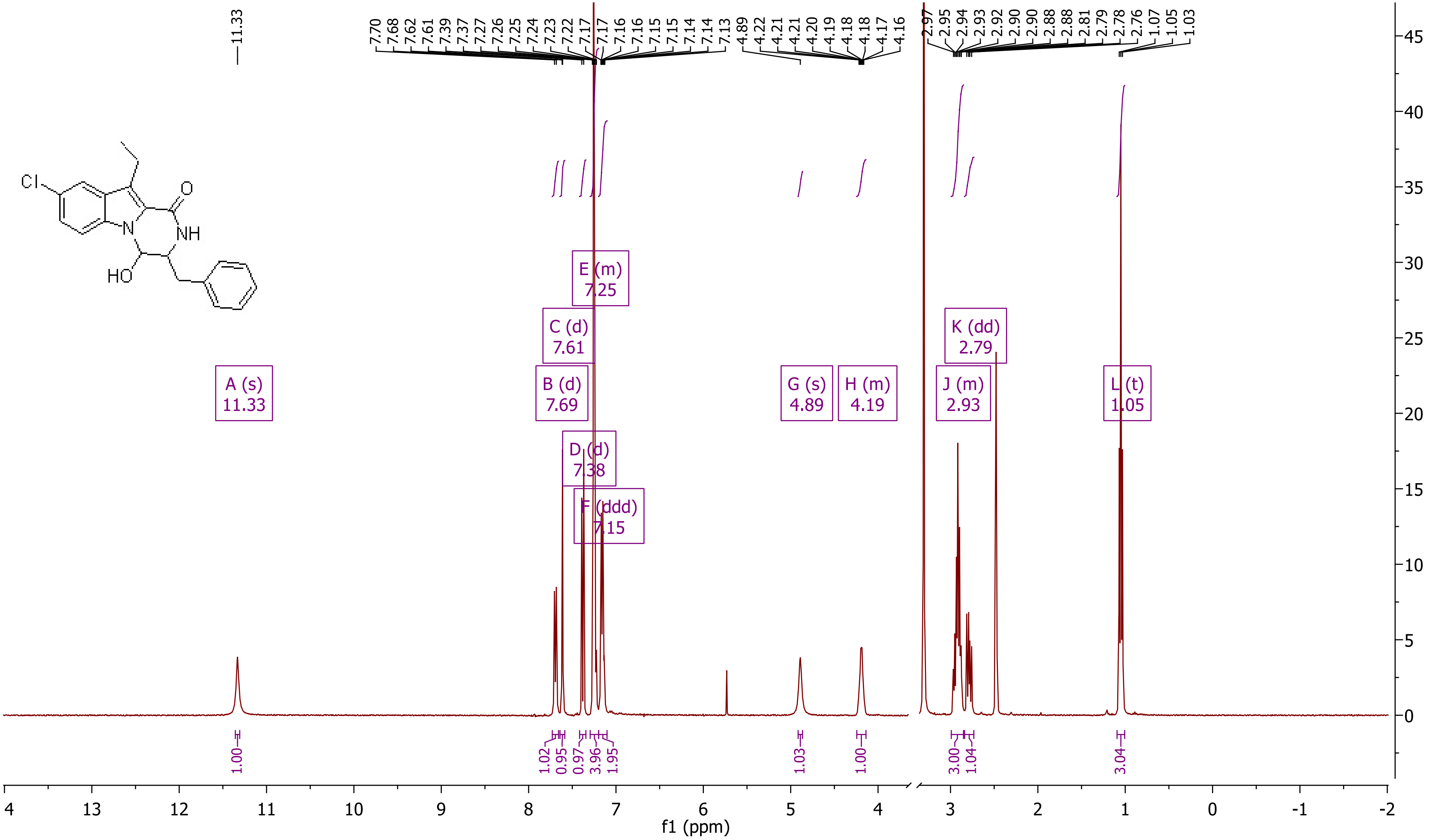

^{1}H NMR (400 MHz, DMSO-d_6) δ 11.33 (s, 1H), 7.69 (d, J = 8.3 Hz, 1H), 7.61 (d, J = 2.1 Hz, 1H), 7.38 (d, J = 8.7 Hz, 1H), 7.30 – 7.20 (m, 4H), 7.15 (dd, J = 8.4, 2.2 Hz, 2H), 4.89 (s, 1H), 4.24 – 4.14 (m, 1H), 2.99 – 2.85 (m, 3H), 2.79 (dd, J = 13.8, 8.4 Hz, 1H), 1.05 (t, J = 7.4 Hz, 3H).

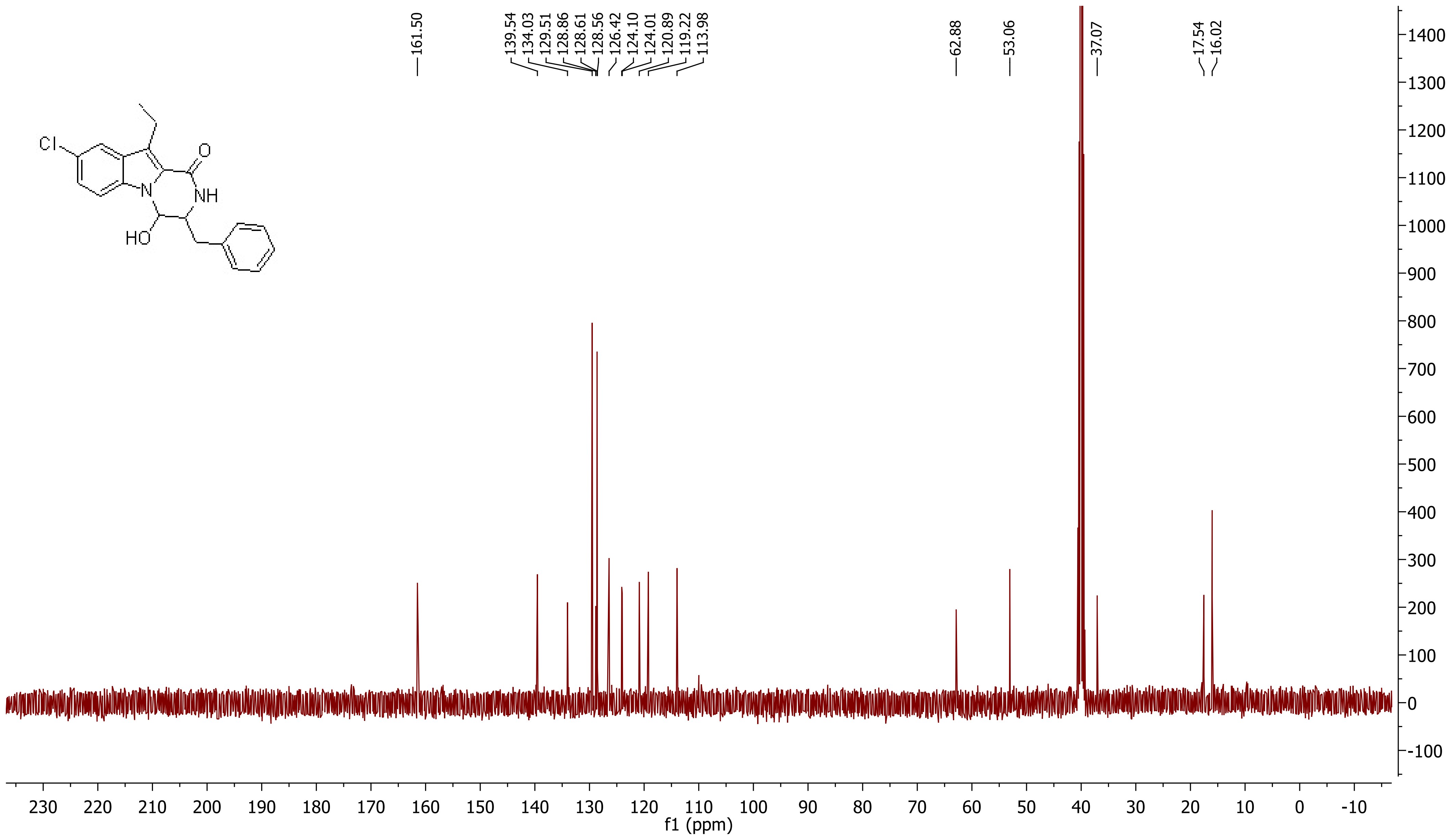

^{13}C NMR (101 MHz, dmso) δ 161.50, 139.54, 134.03, 129.51, 128.86, 128.61, 128.56, 126.42, 124.10, 124.01, 120.89, 119.22, 113.98, 62.88, 53.06, 37.07, 17.54, 16.02.

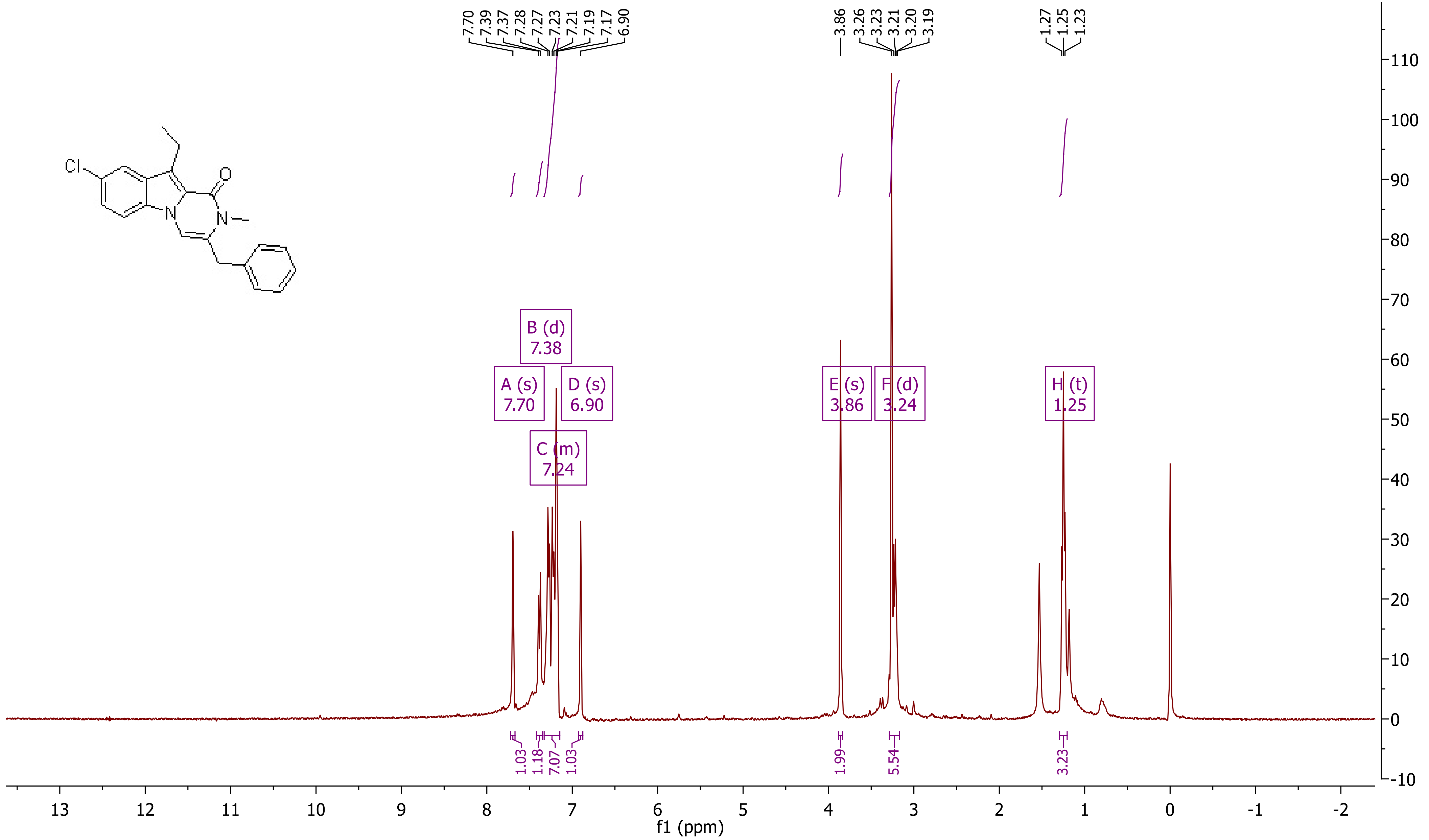

^{1}H NMR (400 MHz, Chloroform-*d*) δ 7.70 (s, 1H), 7.38 (d, *J* = 9.0 Hz, 1H), 7.33 – 7.15 (m, 6H), 6.90 (s, 1H), 3.86 (s, 2H), 3.26 - 3.19 (m, 5H), 1.25 (t, *J* = 7.6 Hz, 3H).

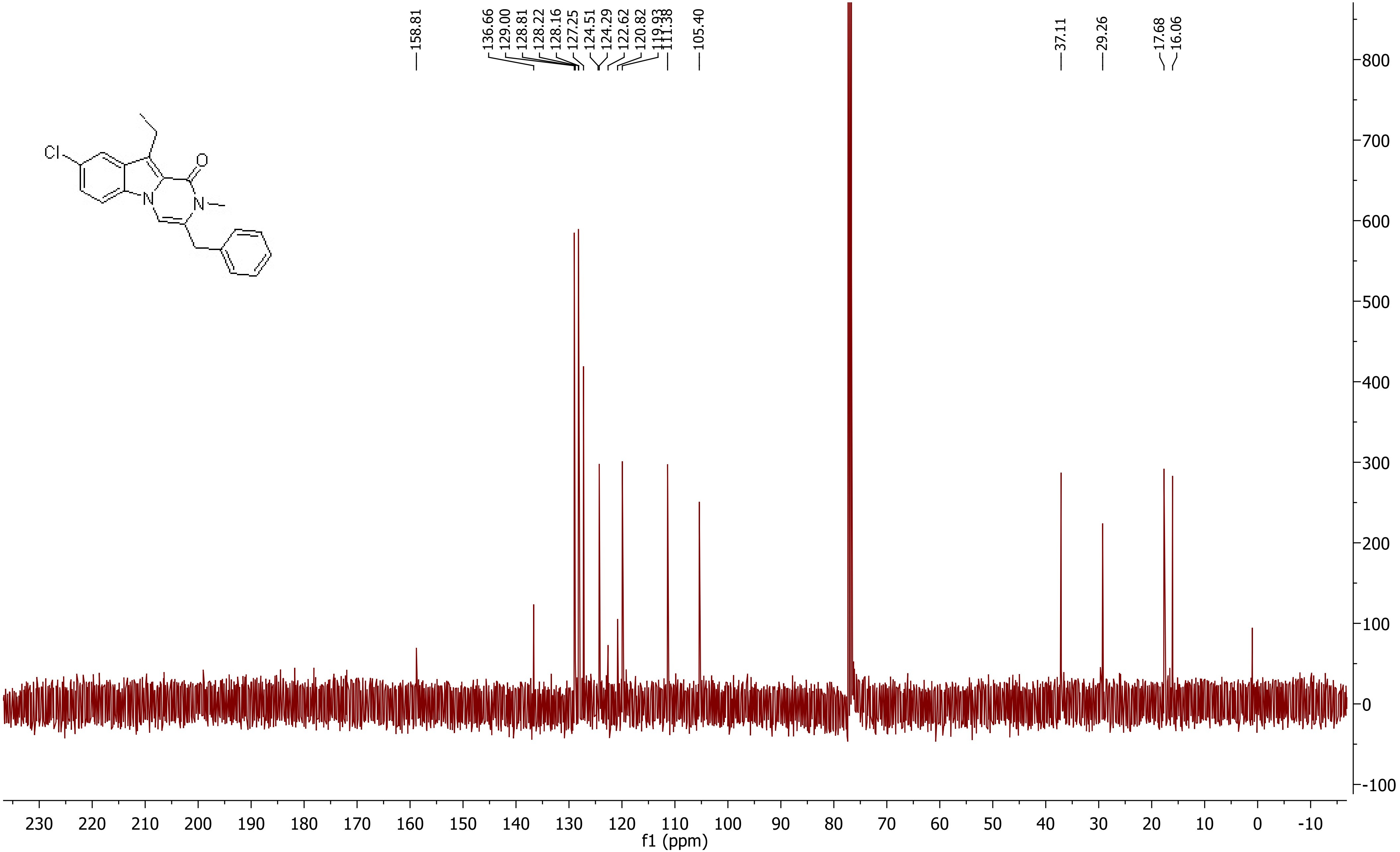

^{13}C NMR (101 MHz, cdcl$_3$) δ 158.81, 136.66, 129.00, 128.81, 128.22, 128.16, 127.25, 124.51, 124.29, 122.62, 120.82, 119.93, 111.38, 105.40, 37.11, 29.26, 17.68, 16.06.

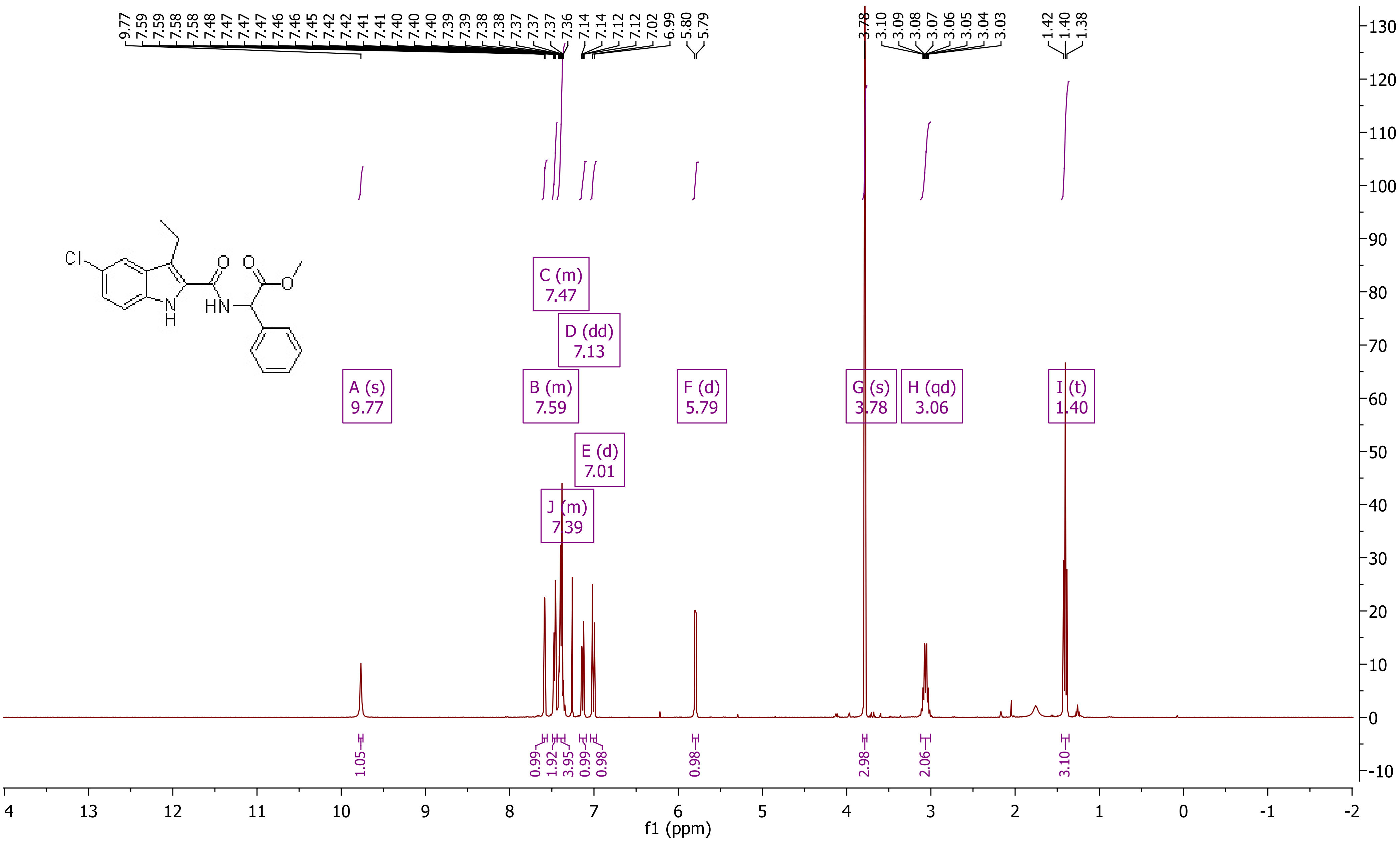

^{1}H NMR (400 MHz, Chloroform-*d*) δ 9.77 (s, 1H), 7.59 (d, *J* = 2.0 Hz, 1H), 7.49 – 7.44 (m, 2H), 7.44 – 7.34 (m, 4H), 7.13 (dd, *J* = 8.7, 2.0 Hz, 1H), 7.01 (d, *J* = 8.7 Hz, 1H), 5.79 (d, *J* = 6.4 Hz, 1H), 3.78 (s, 3H), 3.06 (q, *J* = 7.6 Hz, 2H), 1.40 (t, *J* = 7.6 Hz, 3H).

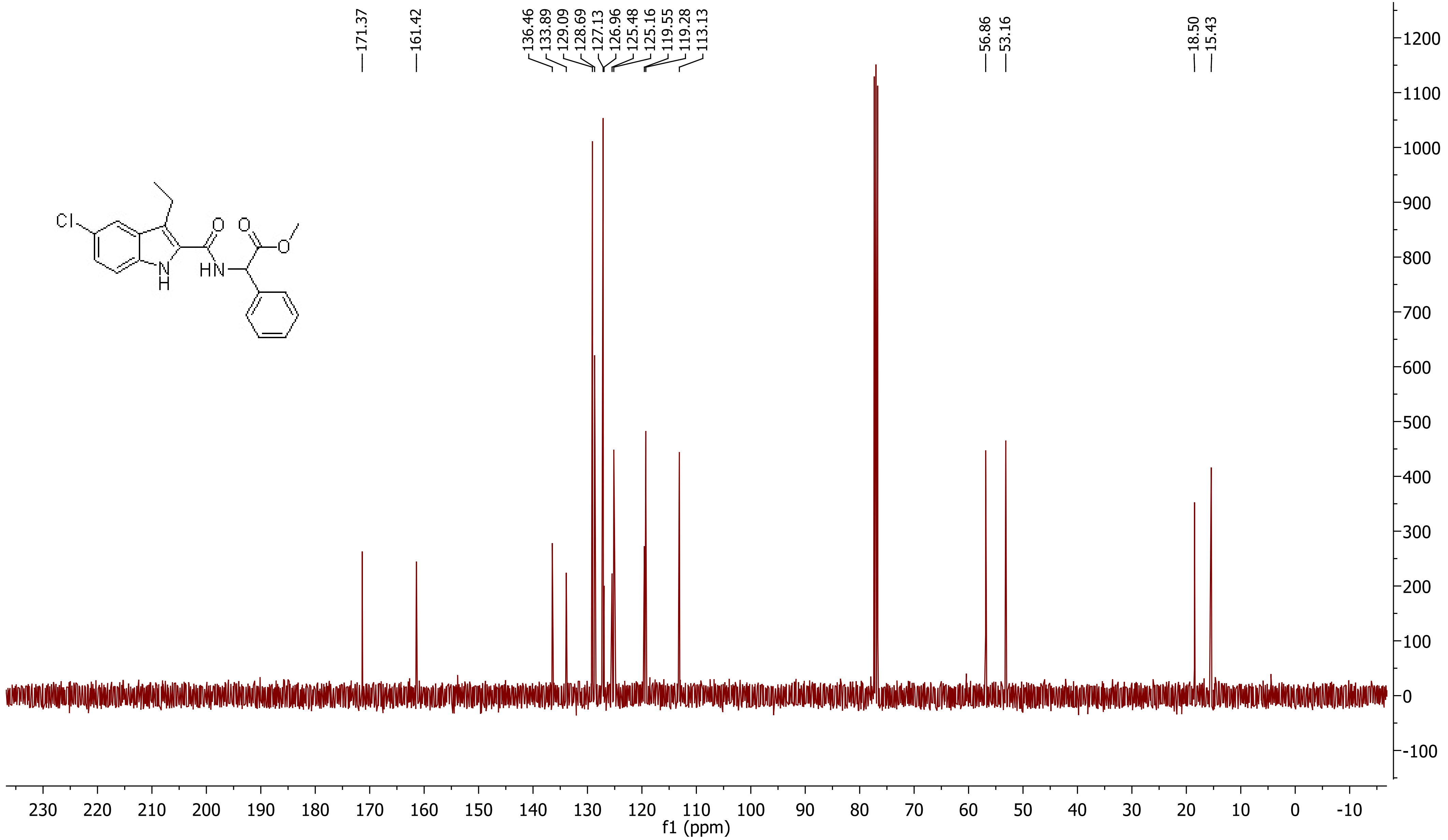

^{13}C NMR (101 MHz, cdcl$_3$) δ 171.37, 161.42, 136.46, 133.89, 129.09, 128.69, 127.13, 126.96, 125.48, 125.16, 119.55, 119.28, 113.13, 56.86, 53.16, 18.50, 15.43.

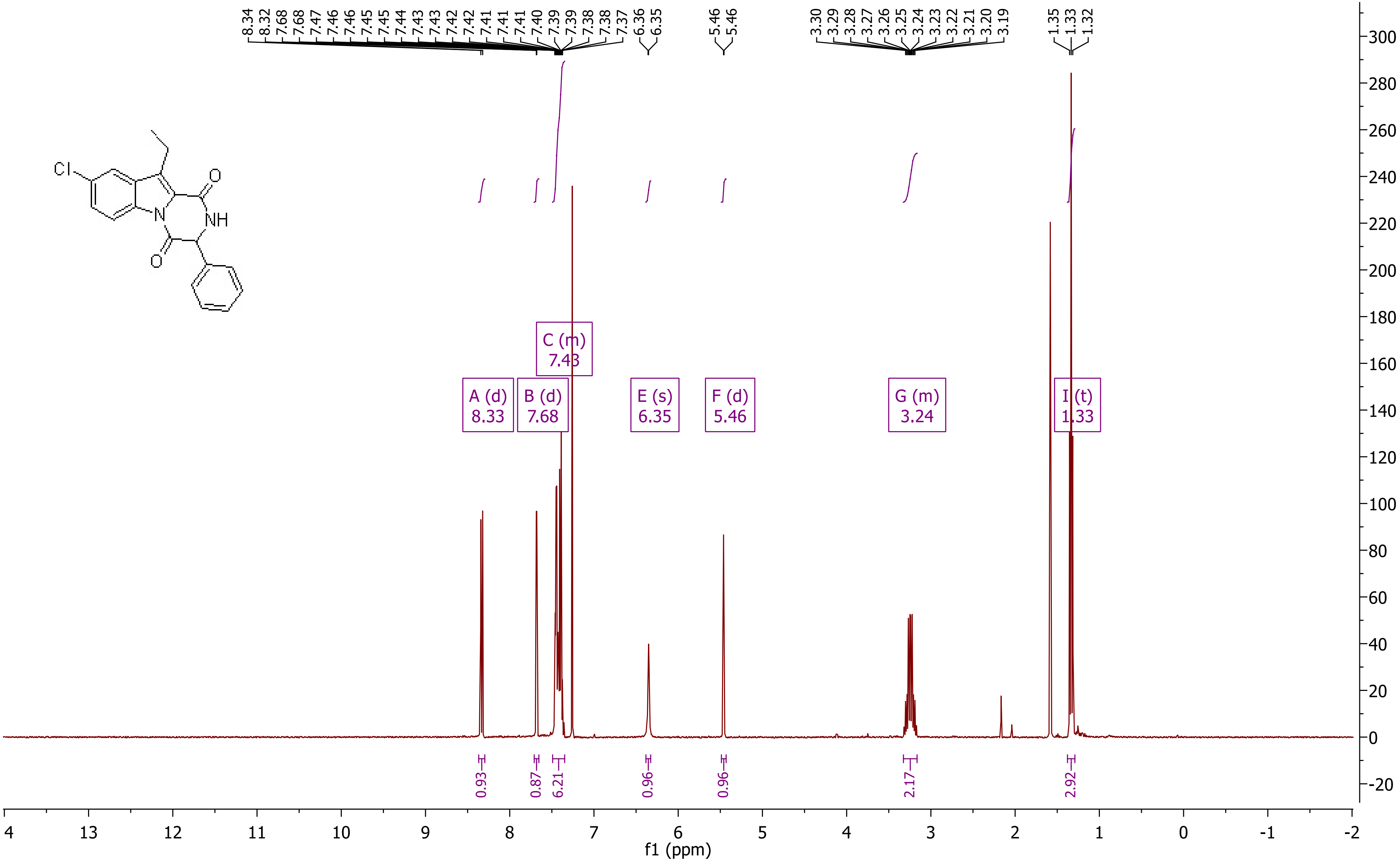

^{1}H NMR (400 MHz, Chloroform-*d*) δ 8.33 (d, *J* = 8.8 Hz, 1H), 7.68 (d, *J* = 2.0 Hz, 1H), 7.49 – 7.35 (m, 6H), 6.35 (s, 1H), 5.46 (d, *J* = 2.2 Hz, 1H), 3.24 (q, *J* = 7.4 Hz, 2H), 1.33 (t, *J* = 7.5 Hz, 3H).

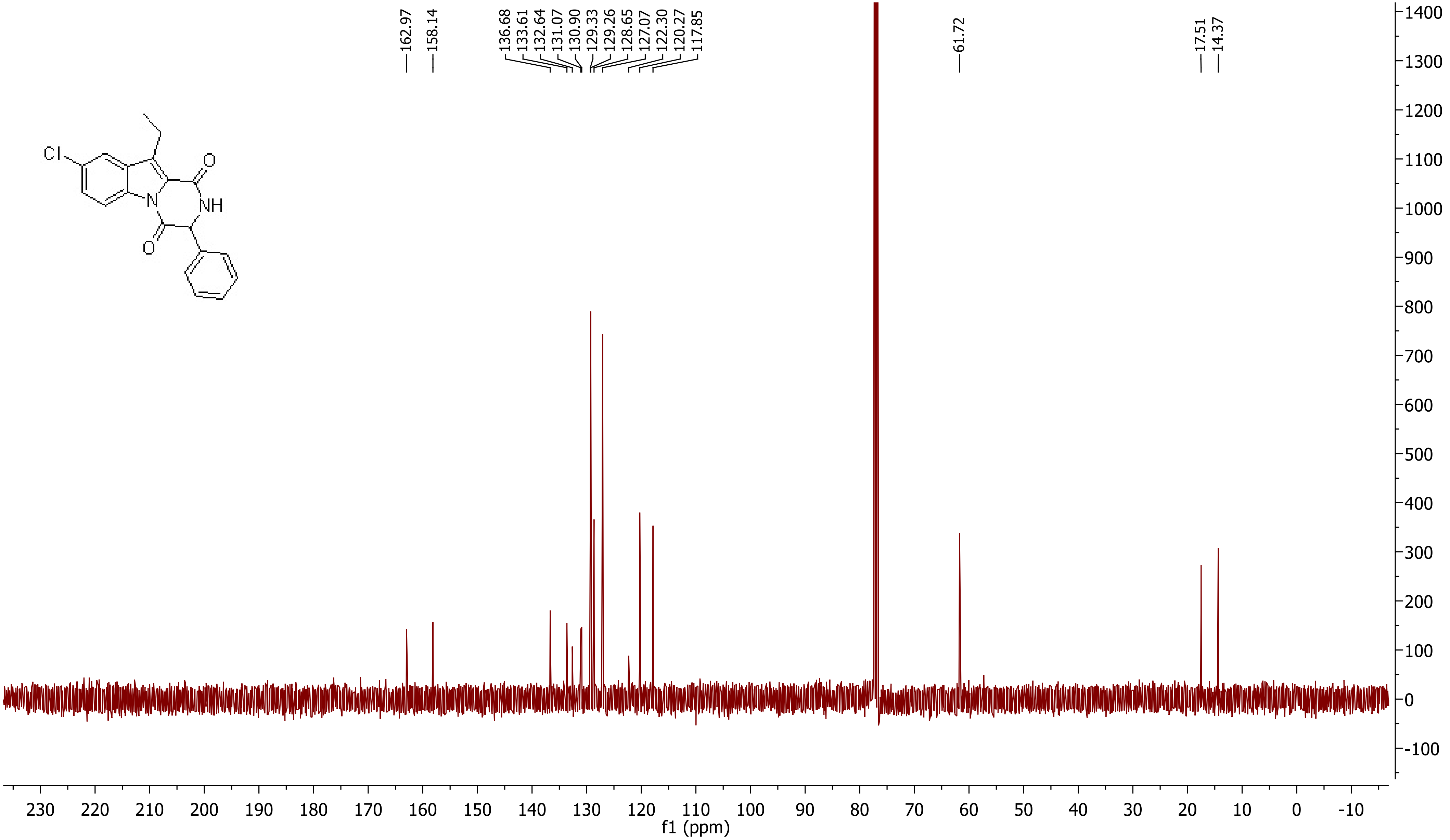

^{13}C NMR (101 MHz, cdcl$_3$) δ 162.97, 158.14, 136.68, 133.61, 132.64, 131.07, 130.90, 129.33, 129.26, 128.65, 127.07, 122.30, 120.27, 117.85, 61.72, 17.51, 14.37.

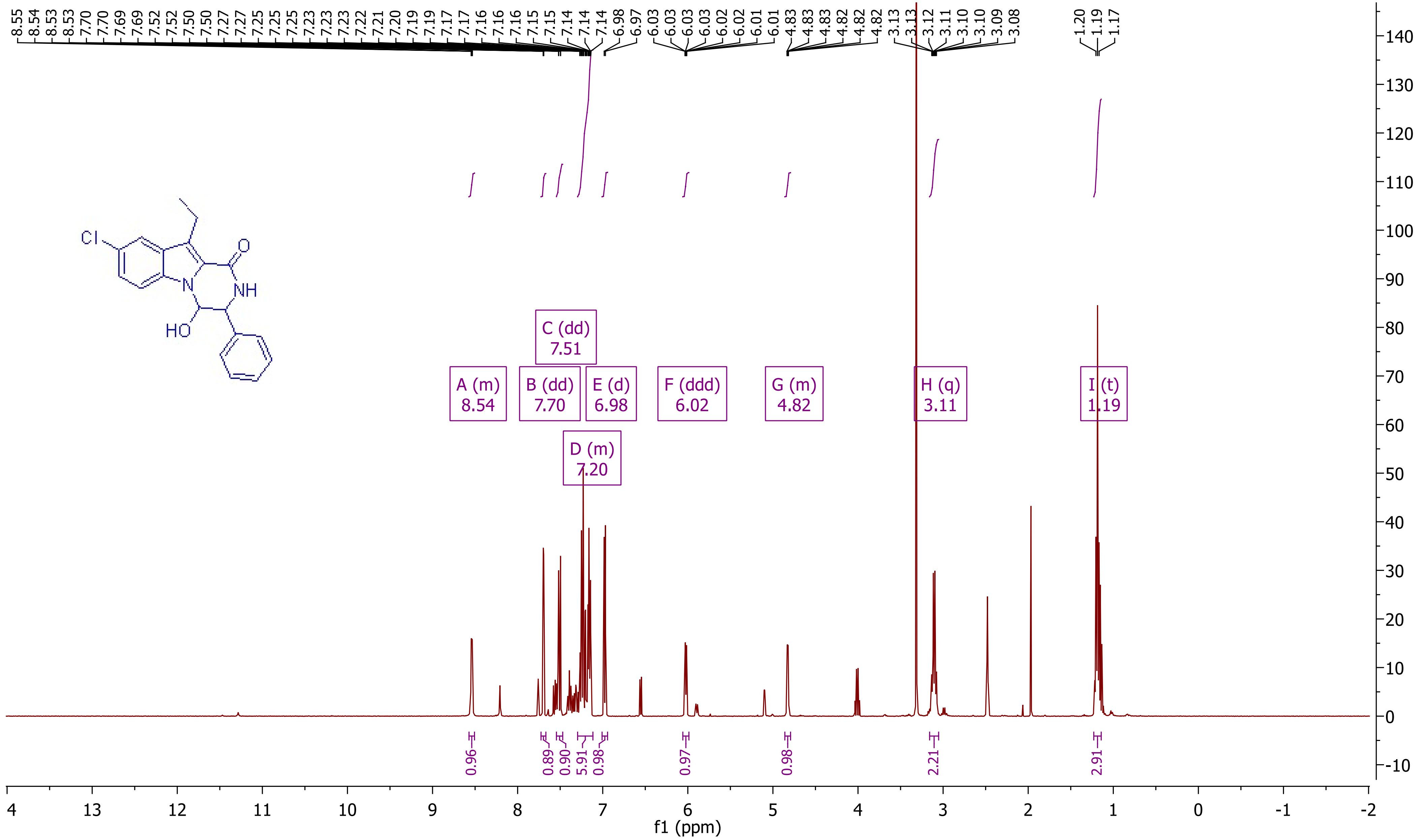

^{1}H NMR (400 MHz, DMSO-d_6) δ 8.54 (d, J = 4.7 Hz, 1H), 7.70 (d, J = 2.1 Hz, 1H), 7.51 (d, J = 8.9 Hz, 1H), 7.29 – 7.12 (m, 6H), 6.98 (d, J = 5.7 Hz, 1H), 6.02 (d, J = 5.8 Hz, 1H), 4.86 – 4.79 (m, 1H), 3.11 (q, J = 7.5 Hz, 2H), 1.19 (t, J = 7.4 Hz, 3H).

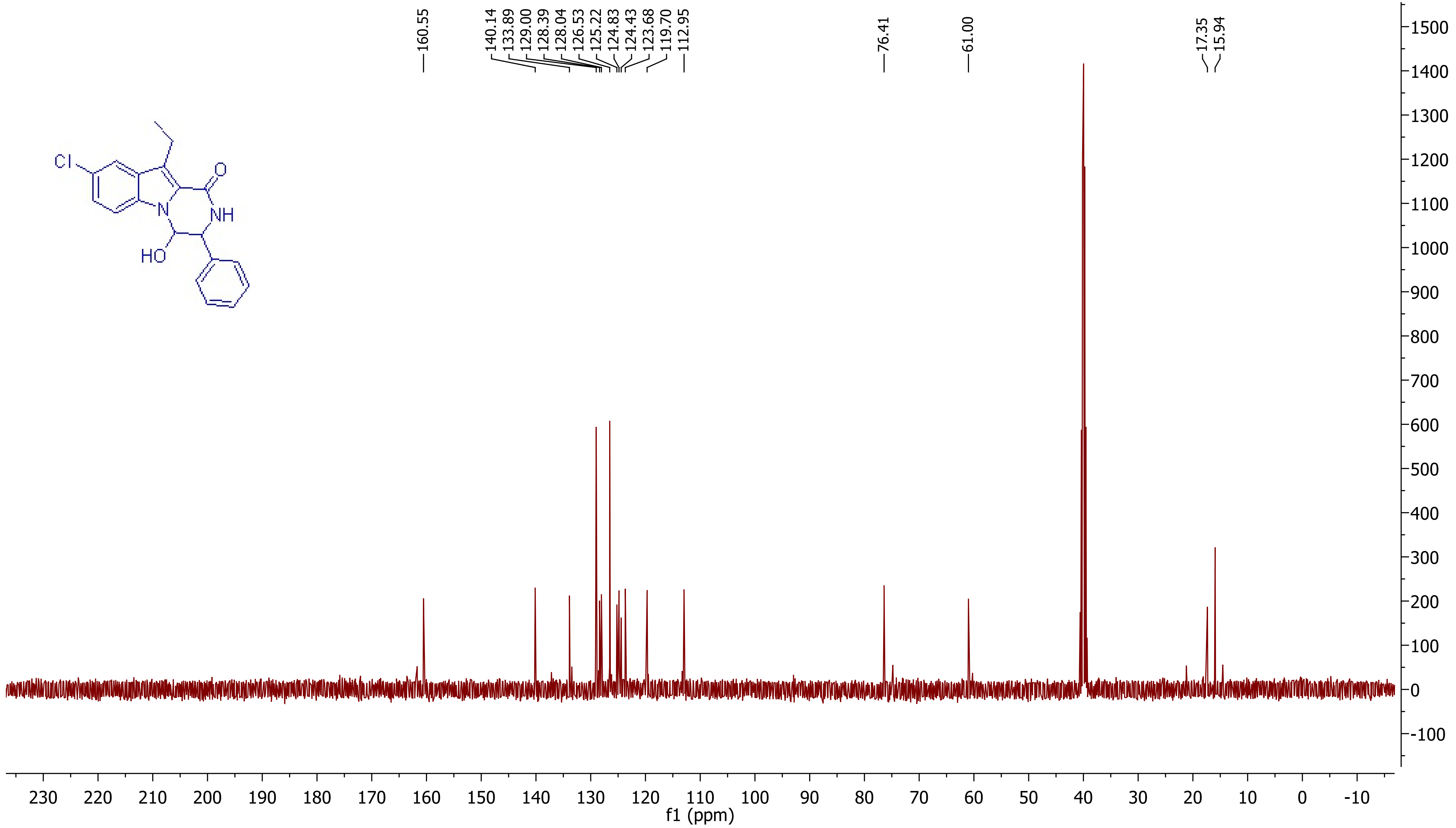

^{13}C NMR (101 MHz, dmso) δ 160.55, 140.14, 133.89, 129.00, 128.39, 128.04, 126.53, 125.22, 124.83, 124.43, 123.68, 119.70, 112.95, 76.41, 61.00, 17.35, 15.94.

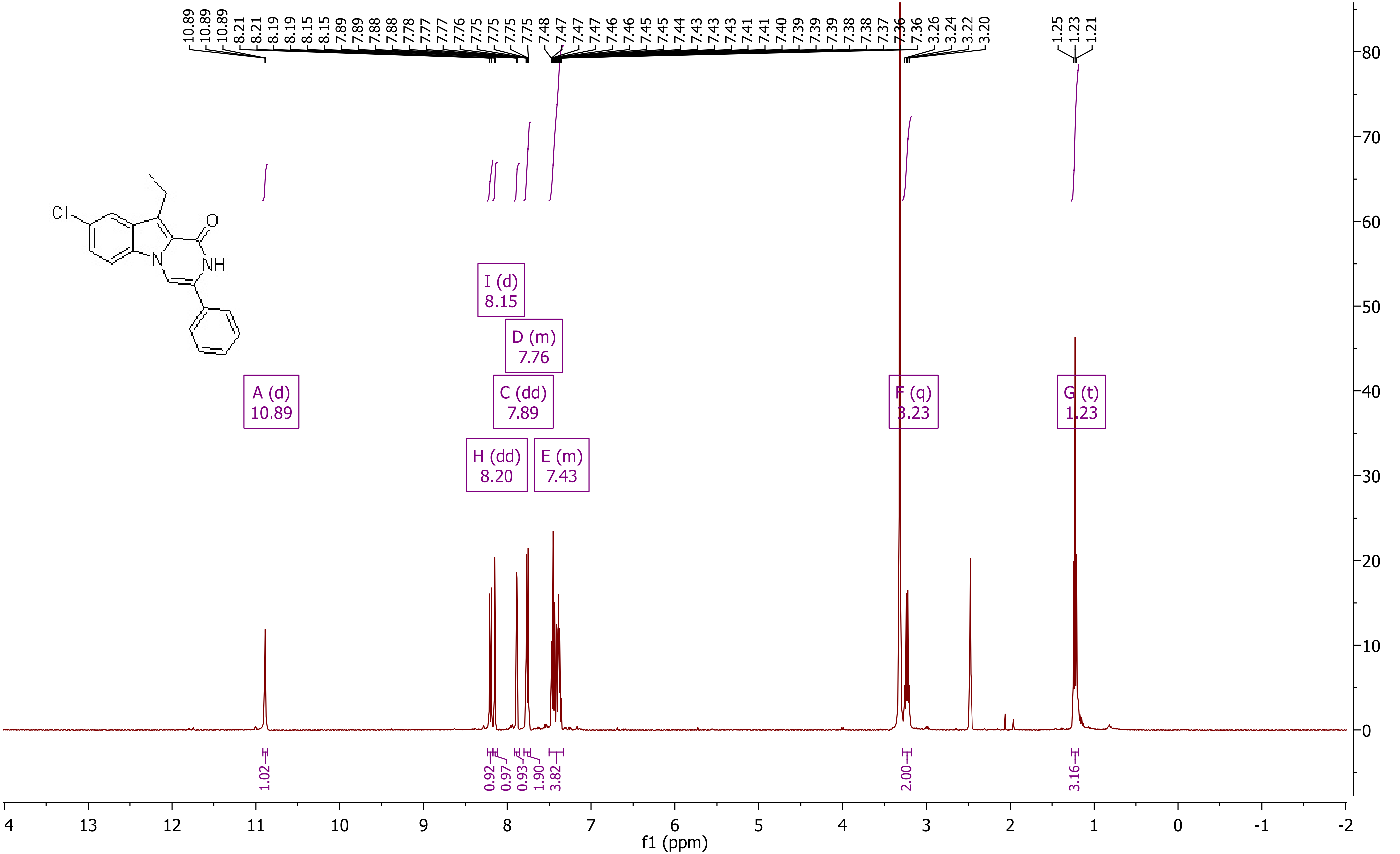

^{1}H NMR (400 MHz, DMSO-d_6) δ 10.89 (s, 1H), 8.20 (d, J = 8.9 Hz, 1H), 8.15 (d, J = 1.6 Hz, 1H), 7.89 (d, J = 2.1 Hz, 1H), 7.80 – 7.72 (m, 2H), 7.50 – 7.33 (m, 4H), 3.23 (q, J = 7.4 Hz, 2H), 1.23 (t, J = 7.4 Hz, 3H).

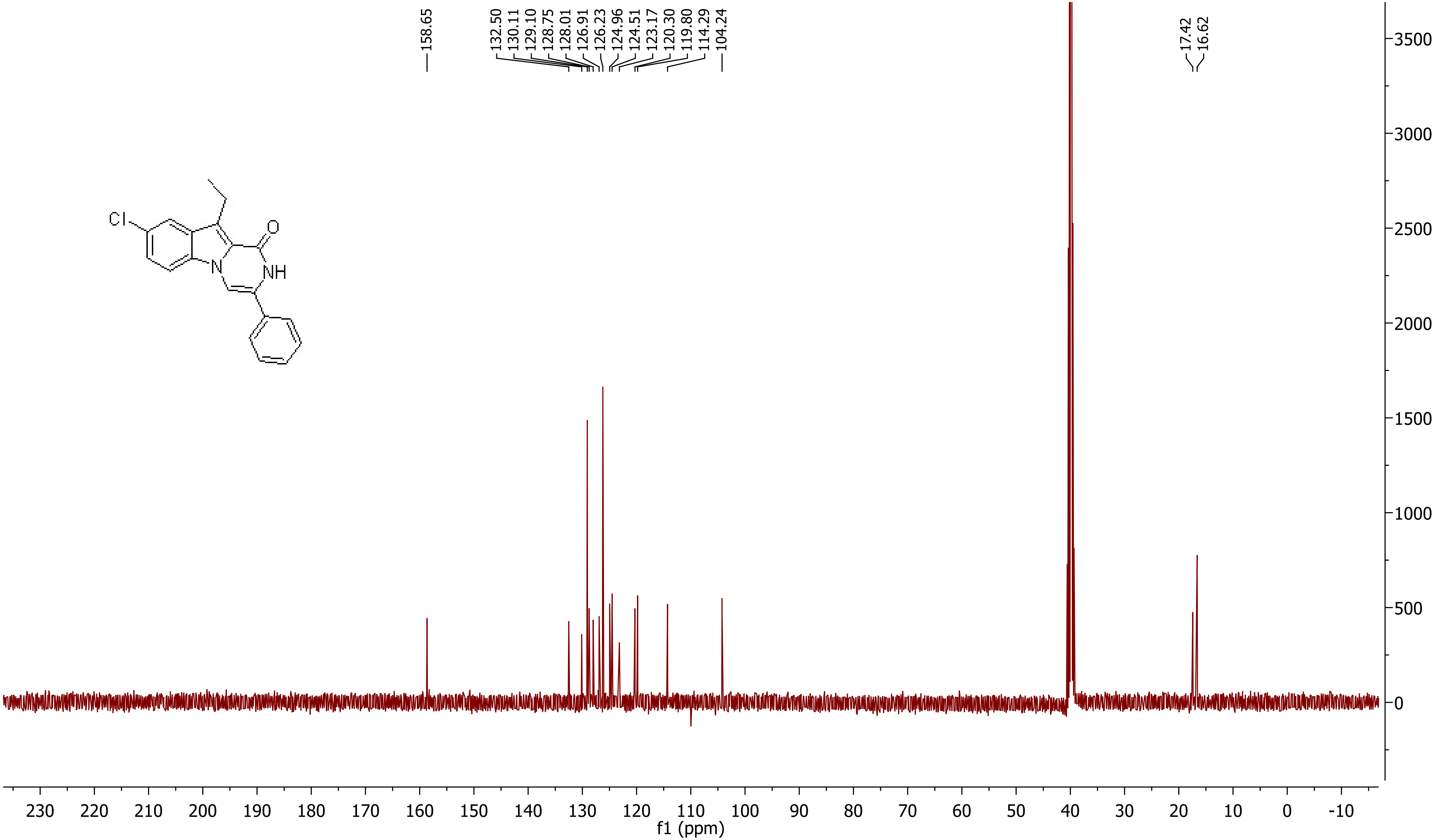

^{13}C NMR (101 MHz, dmso) δ 158.65, 132.50, 130.11, 129.10, 128.75, 128.01, 126.91, 126.23, 124.96, 124.51, 123.17, 120.30, 119.80, 114.29, 104.24, 17.42, 16.62.

Appendix A

4. EXPERIMENTAL

4.1. Chemistry

General Details

All the chemicals used were of analytical grade and purified by standard methods prior to use. Silica gel column chromatography was carried out using kieselgel 60 (Merck). TLC analysis was performed on aluminium-backed plates coated with silica gel 60 F_{254} (Merck). Melting points were determined using a Gallen Kamp melting point apparatus and are uncorrected. Components were visualized using potassium permanganate solution and UV light. NMR Spectra were taken using a Varian Unity INOVA 400 MHz and Bruker AC250 MHz spectrometers for proton and carbon at university of Aberdeen. All numbers referring to NMR data obtained are in parts per million (ppm).

4.2. Biological evaluation

4.2.1 Cell Viability assay (MTT assay)

MTT assay was performed to investigate the effect of the synthesized compounds on mammary epithelial cells (MCF-10A). The cells were propagated in medium consisting of Ham's F-12 medium/ Dulbecco's modified Eagle's medium (DMEM) (1:1) supplemented with 10% foetal calf serum, 2 mM glutamine, insulin (10 µg/mL), hydrocortisone (500 ng/mL) and epidermal growth factor (20 ng/mL). Trypsin ethylenediamine tetra acetic acid (EDTA) was used to passage the cells after every 2-3 days. 96-well flat-bottomed cell culture plates were used to seed the cells at a density of 104 cells mL^{-1}. The medium was aspirated from all the wells of culture plates after 24 h followed by the addition of synthesized compounds (in 200 µL medium to yield a final concentration of 0.1% (v/v) dimethyl sulfoxide) into individual wells of the plates. Four wells were designated to a single compound. The plates were allowed to incubate at 37°C for 96 h. Afterwards, the medium was aspirated and 3-[4,5-dimethylthiazol-2-yl]-2,5-diphenyltetrazolium bromide (MTT) (0.4 mg/mL) in medium was added to each well and subsequently incubated for 3 h. The medium was aspirated and 150 µL dimethyl sulfoxide (DMSO) was added to each well. The plates were vortexed followed by the measurement of absorbance at 540 nm on a microplate reader. The results were presented as inhibition (%) of proliferation in contrast to controls comprising 0.1% DMSO.

4.2.2. Assay for antiproliferative effect

To explore the antiproliferative potential of compounds propidium iodide fluorescence assay was performed using different cell lines such as Panc-1 (pancreas cancer cell line), MCF-7 (breast cancer cell line), HT-29 (colon cancer cell line) and A-549 (epithelial cancer cell line), respectively. To calculate the total nuclear DNA, a fluorescent dye (propidium iodide, PI) is used which can attach to the DNA, thus offering a quick and precise technique. PI cannot pass through the cell membrane and its signal intensity can be considered as directly proportional to quantity of cellular DNA. Cells whose cell membranes are damaged or have changed permeability are counted as dead ones. The assay was performed by seeding the cells of different cell lines at a density of 3000-7500 cells/well (in200µl medium) in culture plates followed by incubation for 24h at 37 °C in humidified 5%CO2/95% air atmospheric conditions. The medium was removed; the compounds were added to the plates at 10 µM concentrations (in 0.1% DMSO) in triplicates, followed by incubation for 48h. DMSO (0.1%) was used as control. After incubation, medium was removed followed by the addition of PI (25 µl, 50µg/mL in water/medium) to each well of the plates. At -80 °C, the plates were allowed to freeze for 24 h, followed by thawing at 25 ^{o}C. A fluorometer (Polar-Star BMG Tech) was used to record the readings at excitation and emission wavelengths of 530 and 620 nm for each well. The percentage cytotoxicity of compounds was calculated using the following formula:

$$\% \text{ Cytotoxicity} = \frac{A_C - A_{TC}}{A_C} \times 100$$

Where A*TC*= Absorbance of treated cells and AC= Absorbance of control. Erlotinib was used as positive control in the assay.

4.2.3. Aromatase inhibitory assay

The aromatase inhibitory effect was performed using the method reported by Stressor et al.[35]. This method was carried out according to the Gentest kit using CYP19 enzyme and DBF as a fluorometric substrate. DBF was dealkylated by aromatase and then hydrolyzed to give the fluorescein product. Briefly, 100 µL of cofactor, containing 78.4 µL of 50 mM phosphate buffer (pH 7.4); 20 µL of 20x NADPH-generating system (26 mM $NADP^+$, 66 mM glucose-6-phosphate, and 66 mM $MgCl_2$); and 1.6 µL of 100 U/mL glucose-6-phosphate dehydrogenase, was pipetted into a 96-well plate and preincubated in 37 ^oC (water bath) for 10 min. The reaction was initiated by addition of 100 µL of enzyme/substrate mixture containing 77.3 µL of 50 mM
phosphate buffer (pH 7.4); 12.5 µL of 16 pmol/mL CYP19; 0.2 µL of 0.2 mM DBF, and 10 µL of 0.25 mM diluted tested sample or 10% DMSO as a negative control or letrozole as a positive control. Fluorescence signal was recorded using an excitation wavelength of 490 nm and emission wavelength of 530 nm with cutoff 515 nm. Percentage of inhibition (%inhibition) was calculated as shown in Equation (1). Samples with % inhibition greater than 50 were further diluted and assayed in triplicate. Finally, IC_{50} values were determined by plot of concentrations versus % inhibition.

$$\%\,inhibition = 100 - [(sample - blank)/(DMSO - blank) \times 100]$$

4.2.4. NOS inhibition assay

Recombinant human iNOS was purchased from Enzo Life Sciences, Inc. (New York, USA). Recombinant bovine eNOS was purchased from Cayman Chemical (Ann Arbor, USA). To measure iNOS activity, 10 μL of enzyme stock solution were added to 80 μL of 2-[4-(2-hydroxyethyl) piperazin-1-yl]ethanesulfonic acid (HEPES) buffer pH = 7.4, 100 mM, containing 0.1 mM $CaCl_2$, 1 mM D,L-dithiothreitol (DTT), 0.5 mg/mL BSA, 10 μM flavin mononucleotide (FMN), 10 μM flavin adenine dinucleotide (FAD), 30 μM tetrahydrobiopterin (BH4), 10 μg/mL calmodulin (CaM), 10 μM L-Arg. For the eNOS activity evaluation, 25 μL of the enzyme stock solution were added to 65 μL of HEPES buffer containing 2 mM $CaCl_2$ and the same cofactors cocktail used for the iNOS assay Then, 10 μL of the test compound solution or **1400W** (0.1–100 μM) were added to the enzyme assay solution, followed by pre-incubation of 15 min at 37 ◦C. Each reaction was initiated by the addition of 10 μL of NADPH 7.5 mM, carried out at 37 ◦C for 30 min, and stopped by adding 500 μL of ice-cold CH_3CN. The mixture was brought to dryness under *vacuum* and eventually stored at – 20 ◦C, before the HPLC analysis.

4.2.5. Caspase-3 activation assay

Allow all reagents to reach room temperature before use. Gently mix all liquid reagents prior to use. Determine the number of 8-well strips needed for the assay. Insert these in the frame(s) for current use. Add 100 µl of the *Standard Diluent Buffer* to the zero standard wells. Well(s) reserved for chromogen blank should be left empty. Add 100 µl of standards and controls or diluted samples to the appropriate microtiter wells. The sample dilution chosen should be optimized for each experimental system. Tap gently on side of plate to mix. Cover wells with *plate cover* and incubate for 2 hours at room temperature. Thoroughly aspirate or decant solution from wells and discard the liquid, Wash wells 4 times. Pipette 100 µl of *Caspase-3 (Active) Detection Antibod*y solution

into each well except the chromogen blank(s). Tap gently on the side of the plate to mix. Cover plate with *plate cover* and incubate for 1 hour at room temperature. Thoroughly aspirate or decant solution from wells and discard the liquid, Wash wells 4 times. Add 100 µl Anti-Rabbit IgG HRP Working Solution to each well except the chromogen blank(s). Prepare the working dilution as described in Preparing IgG HRP. Cover wells with the *plate cover* and incubate for 30 minutes at room temperature. Thoroughly aspirate or decant solution from wells and discard the liquid. Wash wells 4 times. Add 100 µl of *Stabilized Chromogen* to each well. The liquid in the wells will begin to turn blue. Incubate for 30 minutes at room temperature and in the dark. The incubation time for chromogen substrate is often determined by the microtiter plate reader used. Many plate readers have the capacity to record a maximum optical density (O.D.) of 2.0. The O.D. values should be monitored, and the substrate reaction stopped before the O.D. of the positive wells exceeds the limits of the instrument. The O.D. values at 450 nm can only be read after the *Stop Solution* has been added to each well. If using a reader that records only to 2.0 O.D., stopping the assay after 20 to 25 minutes is suggested. Add 100 µl of *Stop Solution* to each well. Tap side of plate gently to mix. The solution in the wells should change from blue to yellow. Read the absorbance of each well at 450 nm having blanked the plate reader against a chromogen blank composed of 100 µl each of *Stabilized Chromogen* and *Stop Solution*. Read the plate within 2 hours after adding the *Stop Solution*. Use a curve fitting software to generate the standard curve. A four-parameter algorithm provides the best standard curve fit. Read the concentrations for unknown samples and controls from the standard curve. Multiply value(s) obtained for sample(s) by the appropriate dilution factor to correct for the dilution in step 3. Samples producing signals greater than that of the highest standard should be diluted in *Standard Diluent Buffer* and reanalyzed.

4.2.6. Caspase-8 activation assay

Cells were obtained from American Type Culture Collection, cells were grown in RPMI 1640 containing 10% fetal bovine serum at 37°C, stimulated with the compounds to be tested for caspase8, and lysed with Cell Extraction Buffer. This lysate was diluted in Standard Diluent Buffer over the range of the assay and measured for human active caspase-8 content. (*Cells are Plated in a density of 1.2 – 1.8 × 10,000 cells/well in a volume of 100µl complete growth medium + 100 ul of the tested compound per well in a 96-well plate for 24 hours before the enzyme assay for Tubulin.*). The absorbance of each microwell was read on a spectro-photometer at 450 nm. A standard curve is prepared from 7human Caspase-8 standard dilutions and human Caspase-8 concentration determined.

4.2.7. Bax activation assay

Bring all reagents, except the human Bax-α Standard, to room temperature for at least 30 minutes prior to opening. The human Bax-α Standard solution should not be left at room temperature for more than 10 minutes. All standards, controls and samples should be run in duplicate. Refer to the Assay Layout Sheet to determine the number of wells to be used and put any remaining wells with the desiccant back into the pouch and seal the ziploc. Store unused wells at 4 °C. Pipet 100 µL of Assay Buffer into the S0 (0 pg/mL standard) wells. Pipet 100 µL of Standards #1 through #6 into the appropriate wells. Pipet 100 µL of the Samples into the appropriate wells. Tap the plate gently to mix the contents. Seal the plate and incubate at room temperature on a plate shaker for 1 hour at ~500 rpm. Empty the contents of the wells and wash by adding 400 µL of wash solution to every well. Repeat the wash 4 more times for a total of **5 washes**. After the final wash, empty or aspirate the wells and firmly tap the plate on a lint free paper towel to remove any remaining wash buffer. Pipet 100 µL of yellow Antibody into each well, except the Blank. Seal the plate and incubate at

room temperature on a plate shaker for 1 hour at ~500 rpm. Empty the contents of the wells and wash by adding 400 μL of wash solution to every well. Repeat the wash 4 more times for a total of **5** washes. After the final wash, empty or aspirate the wells and firmly tap the plate on a lint free paper towel to remove any remaining wash buffer. Add 100 μL of blue Conjugate to each well, except the Blank. Seal the plate and incubate at room temperature on a plate shaker for 30 minutes at ~500 rpm. Empty the contents of the wells and wash by adding 400 μL of wash solution to every well. Repeat the wash 4 more times for a total of **5 washes**. After the final wash, empty or aspirate the wells and firmly tap the plate on a lint free paper towel to remove any remaining wash buffer. Pipet 100 μL of Substrate Solution into each well. Incubate for 30 minutes at room temperature on a plate shaker at ~500 rpm. Pipet 100 μL Stop Solution to each well. Blank the plate reader against the Blank wells, read the optical density at 450 nm. Calculate the average net Optical Density (OD) bound for each standard and sample by subtracting the average Blank OD from the average OD for each standard and sample. Using linear graph paper, plot the Average Net OD for each standard versus Bax concentration in each standard. Approximate a straight line through the points. The concentration of Bax in the unknowns can be determined by interpolation.

4.2.8. Bcl-2 inhibition assay

Mix all reagents thoroughly without foaming before use. Wash the microwells twice with approximately 300 μL Wash Buffer per well with thorough aspiration of microwell contents between washes. Take caution not to scratch the surface of the microwells. After the last wash, empty the wells and tap microwell strips on absorbent pad or paper towel to remove excess Wash Buffer. Use the microwell strips immediately after washing or place upside down on a wet absorbent paper for not longer than 15 minutes. Do not allow wells to dry. Add 100 μL of Sample Diluent in duplicate to all standard wells and to the blank wells. Prepare standard (1:2 dilution) in

duplicate ranging from 32 ng/mL to 0.5 ng/mL. Add 100 µL of Sample Diluent, in duplicate, to the blank wells. Add 80 µL of Sample Diluent, in duplicate, to the sample wells. Add 20 µL of each Sample, in duplicate, to the designated wells. Add 50 µL of diluted biotin-conjugate to all wells, including the blank wells. Cover with a plate cover and incubate at room temperature, on a microplate shaker at 100 rpm if available, for 2 hours. Remove plate cover and empty the wells. Wash microwell strips 3 times as described in step 2. Add 100 µL of diluted Streptavidin-HRP to all wells, including the blank wells. Cover with a plate cover and incubate at room temperature, on a microplate shaker at 100 rpm if available, for 1 hour. Remove plate cover and empty the wells. Wash microwell strips 3 times as described in step 2. Proceed to the next step. Pipette 100 µl of mixed TMB Substrate Solution to all wells, including the blanks. Incubate the microwell strips at room temperature (18° to 25°C) for about 15 minutes, if available on a rotator set at 100 rpm. Avoid direct exposure to intense light. The point, at which the substrate reaction is stopped, is often determined by the ELISA reader. Many ELISA readers record absorbance only up to 2.0 O.D. Therefore, the color development within individual microwells must be watched by the person running the assay and the substrate reaction stopped before positive wells are no longer properly detectable. Stop the enzyme reaction by quickly pipetting 100 µL of Stop Solution into each well, including the blank wells. It is important that the Stop Solution is spread quickly and uniformly throughout the microwells to completely inactivate the enzyme. Results must be read immediately after the Stop Solution is added or within one hour if the microwell strips are stored at 2 - 8°C in the dark. Read absorbance of each microwell on a spectrophotometer using 450 nm as the primary wavelength.

4.3. Statistical analysis

Computerized Prism 5 program was used to statistically analyzed data using one-way ANOVA test followed by Tukey's as post ANOVA for multiple comparison at $P \leq .05$. Data were presented as mean ± SEM.
